# Gene-educational attainment interactions in a multi-population genome-wide meta-analysis identify novel lipid loci

**DOI:** 10.3389/fgene.2023.1235337

**Published:** 2023-11-02

**Authors:** Lisa de las Fuentes, Karen L. Schwander, Michael R. Brown, Amy R. Bentley, Thomas W. Winkler, Yun Ju Sung, Patricia B. Munroe, Clint L. Miller, Hugo Aschard, Stella Aslibekyan, Traci M. Bartz, Lawrence F. Bielak, Jin Fang Chai, Ching-Yu Cheng, Rajkumar Dorajoo, Mary F. Feitosa, Xiuqing Guo, Fernando P. Hartwig, Andrea Horimoto, Ivana Kolčić, Elise Lim, Yongmei Liu, Alisa K. Manning, Jonathan Marten, Solomon K. Musani, Raymond Noordam, Sandosh Padmanabhan, Tuomo Rankinen, Melissa A. Richard, Paul M. Ridker, Albert V. Smith, Dina Vojinovic, Alan B. Zonderman, Maris Alver, Mathilde Boissel, Kaare Christensen, Barry I. Freedman, Chuan Gao, Franco Giulianini, Sarah E. Harris, Meian He, Fang-Chi Hsu, Brigitte Kühnel, Federica Laguzzi, Xiaoyin Li, Leo-Pekka Lyytikäinen, Ilja M. Nolte, Alaitz Poveda, Rainer Rauramaa, Muhammad Riaz, Antonietta Robino, Tamar Sofer, Fumihiko Takeuchi, Bamidele O. Tayo, Peter J. van der Most, Niek Verweij, Erin B. Ware, Stefan Weiss, Wanqing Wen, Lisa R. Yanek, Yiqiang Zhan, Najaf Amin, Dan E. Arking, Christie Ballantyne, Eric Boerwinkle, Jennifer A. Brody, Ulrich Broeckel, Archie Campbell, Mickaël Canouil, Xiaoran Chai, Yii-Der Ida Chen, Xu Chen, Kumaraswamy Naidu Chitrala, Maria Pina Concas, Ulf de Faire, Renée de Mutsert, H. Janaka de Silva, Paul S. de Vries, Ahn Do, Jessica D. Faul, Virginia Fisher, James S. Floyd, Terrence Forrester, Yechiel Friedlander, Giorgia Girotto, C. Charles Gu, Göran Hallmans, Sami Heikkinen, Chew-Kiat Heng, Georg Homuth, Steven Hunt, M. Arfan Ikram, David R. Jacobs, Maryam Kavousi, Chiea Chuen Khor, Tuomas O. Kilpeläinen, Woon-Puay Koh, Pirjo Komulainen, Carl D. Langefeld, Jingjing Liang, Kiang Liu, Jianjun Liu, Kurt Lohman, Reedik Mägi, Ani W. Manichaikul, Colin A. McKenzie, Thomas Meitinger, Yuri Milaneschi, Matthias Nauck, Christopher P. Nelson, Jeffrey R. O’Connell, Nicholette D. Palmer, Alexandre C. Pereira, Thomas Perls, Annette Peters, Ozren Polašek, Olli T. Raitakari, Kenneth Rice, Treva K. Rice, Stephen S. Rich, Charumathi Sabanayagam, Pamela J. Schreiner, Xiao-Ou Shu, Stephen Sidney, Mario Sims, Jennifer A. Smith, John M. Starr, Konstantin Strauch, E. Shyong Tai, Kent D. Taylor, Michael Y. Tsai, André G. Uitterlinden, Diana van Heemst, Melanie Waldenberger, Ya-Xing Wang, Wen-Bin Wei, Gregory Wilson, Deng Xuan, Jie Yao, Caizheng Yu, Jian-Min Yuan, Wei Zhao, Diane M. Becker, Amélie Bonnefond, Donald W. Bowden, Richard S. Cooper, Ian J. Deary, Jasmin Divers, Tõnu Esko, Paul W. Franks, Philippe Froguel, Christian Gieger, Jost B. Jonas, Norihiro Kato, Timo A. Lakka, Karin Leander, Terho Lehtimäki, Patrik K. E. Magnusson, Kari E. North, Ioanna Ntalla, Brenda Penninx, Nilesh J. Samani, Harold Snieder, Beatrice Spedicati, Pim van der Harst, Henry Völzke, Lynne E. Wagenknecht, David R. Weir, Mary K. Wojczynski, Tangchun Wu, Wei Zheng, Xiaofeng Zhu, Claude Bouchard, Daniel I. Chasman, Michele K. Evans, Ervin R. Fox, Vilmundur Gudnason, Caroline Hayward, Bernardo L. Horta, Sharon L. R. Kardia, Jose Eduardo Krieger, Dennis O. Mook-Kanamori, Patricia A. Peyser, Michael M. Province, Bruce M. Psaty, Igor Rudan, Xueling Sim, Blair H. Smith, Rob M. van Dam, Cornelia M. van Duijn, Tien Yin Wong, Donna K. Arnett, Dabeeru C. Rao, James Gauderman, Ching-Ti Liu, Alanna C. Morrison, Jerome I. Rotter, Myriam Fornage

**Affiliations:** ^1^ Cardiovascular Division, Department of Medicine, Washington University School of Medicine, St. Louis, MO, United States; ^2^ Division of Biostatistics, Washington University School of Medicine, St. Louis, MO, United States; ^3^ Division of Statistical Genomics, Department of Genetics, Washington University School of Medicine, St. Louis, MO, United States; ^4^ Human Genetics Center, Department of Epidemiology, Human Genetics and Environmental Sciences, School of Public Health, The University of Texas Health Science Center at Houston, Houston, TX, United States; ^5^ Center for Research on Genomics and Global Health, National Human Genome Research Institute, National Institutes of Health, Bethesda, MD, United States; ^6^ Department of Genetic Epidemiology, University of Regensburg, Regensburg, Germany; ^7^ Department of Psychiatry, Washington University School of Medicine, St. Louis, MO, United States; ^8^ Clinical Pharmacology, Queen Mary University of London, London, United Kingdom; ^9^ National Institute for Health Research Barts Cardiovascular Biomedical Research Unit, Queen Mary University of London, London, United Kingdom; ^10^ Center for Public Health Genomics, Department of Public Health Sciences, University of Virginia, Charlottesville, VA, United States; ^11^ Biochemistry and Molecular Genetics, Department of Public Health Sciences, University of Virginia, Charlottesville, VA, United States; ^12^ Department of Biochemistry and Molecular Genetics, University of Virginia, Charlottesville, VA, United States; ^13^ Department of Epidemiology, Harvard School of Public Health, Boston, MA, United States; ^14^ Département de Génomes et Génétique, Institut Pasteur de Lille, Université de Lille, Lille, France; ^15^ School of Public Health, Epidemiology, University of Alabama at Birmingham, Birmingham, AL, United States; ^16^ Cardiovascular Health Research Unit, University of Washington, Seattle, WA, United States; ^17^ Department of Biostatistics, University of Washington, Seattle, WA, United States; ^18^ Department of Epidemiology, School of Public Health, University of Michigan, Ann Arbor, MI, United States; ^19^ Saw Swee Hock School of Public Health, National University of Singapore and National University Health System, Singapore, Singapore; ^20^ Ocular Epidemiology, Singapore Eye Research Institute, Singapore National Eye Centre, Singapore, Singapore; ^21^ Ophthalmology and Visual Sciences Academic Clinical Program, Medical School, Duke-National University of Singapore, Singapore, Singapore; ^22^ Genome Institute of Singapore, Agency for Science, Technology and Research, Singapore, Singapore; ^23^ Department of Pediatrics, The Institute for Translational Genomics and Population Sciences, The Lundquist Institute for Biomedical Innovation at Harbor-UCLA Medical Center, Los Angeles, CA, United States; ^24^ Postgraduate Programme in Epidemiology, Faculty of Medicine, Federal University of Pelotas, Pelotas, RS, Brazil; ^25^ Medical Research Council Integrative Epidemiology Unit, University of Bristol, Bristol, United Kingdom; ^26^ Laboratory of Genetics and Molecular Cardiology, Heart Institute, University of Sao Paulo Medical School, Sao Paulo, SP, Brazil; ^27^ University of Split School of Medicine, Split, Croatia; ^28^ Algebra University College, Zagreb, Croatia; ^29^ Department of Biostatistics, Boston University School of Public Health, Boston, MA, United States; ^30^ Division of Cardiology, Department of Medicine, Duke Molecular Physiology Institute, Duke University School of Medicine, Durham, NC, United States; ^31^ Clinical and Translational Epidemiology Unit, Massachusetts General Hospital, Boston, MA, United States; ^32^ Department of Medicine, Harvard Medical School, Boston, MA, United States; ^33^ Medical Research Council Human Genetics Unit, Institute of Genetics and Cancer, University of Edinburgh, Edinburgh, United Kingdom; ^34^ Jackson Heart Study, Department of Medicine, University of Mississippi Medical Center, Jackson, MS, United States; ^35^ Section of Gerontology and Geriatrics, Department of Internal Medicine, Leiden University Medical Center, Leiden, Netherlands; ^36^ Institute of Cardiovascular and Medical Sciences, University of Glasgow, Glasgow, United Kingdom; ^37^ Human Genomics Laboratory, Pennington Biomedical Research Center, Baton Rouge, LA, United States; ^38^ Brown Foundation Institute of Molecular Medicine, The University of Texas Health Science Center at Houston, Houston, TX, United States; ^39^ Division of Preventive Medicine, Brigham and Women’s Hospital, Boston, MA, United States; ^40^ Harvard Medical School, Boston, MA, United States; ^41^ Department of Biostatistics, School of Public Health, University of Michigan, Ann Arbor, MI, United States; ^42^ Icelandic Heart Association, Kopavogur, Iceland; ^43^ Department of Epidemiology, Erasmus MC, University Medical Center, Rotterdam, Netherlands; ^44^ Molecular Epidemiology, Department of Biomedical Data Sciences, Leiden University Medical Center, Leiden, Netherlands; ^45^ Laboratory of Epidemiology and Population Sciences, National Institute on Aging, National Institutes of Health, Baltimore, MD, United States; ^46^ National Institutes of Health, Baltimore, MD, United States; ^47^ Estonian Genome Center, Insititute of Genomics, University of Tartu, Tartu, Estonia; ^48^ European Genomic Institute for Diabetes, Institut Pasteur de Lille, Lille, France; ^49^ University of Lille, Lille University Hospital, Lille, France; ^50^ Unit of Epidemiology, Biostatistics and Biodemography, Department of Public Health, University of Southern Denmark, Odense, Denmark; ^51^ Nephrology Division, Department of Internal Medicine, Wake Forest School of Medicine, Winston-Salem, NC, United States; ^52^ Molecular Genetics and Genomics Program, Wake Forest School of Medicine, Winston-Salem, NC, United States; ^53^ Department of Psychology, The University of Edinburgh, Edinburgh, United Kingdom; ^54^ Centre for Cognitive Ageing and Cognitive Epidemiology, The University of Edinburgh, Edinburgh, United Kingdom; ^55^ Department of Occupational and Environmental Health and State Key Laboratory of Environmental Health for Incubating, School of Public Health, Tongji Medical College, Huazhong University of Science and Technology, Wuhan, China; ^56^ Department of Biostatistics and Data Science, Division of Public Health Sciences, Wake Forest University School of Medicine, Winston-Salem, NC, United States; ^57^ Research Unit of Molecular Epidemiology, Helmholtz Zentrum München, German Research Center for Environmental Health, Neuherberg, Germany; ^58^ Institute of Epidemiology, Helmholtz Zentrum München, German Research Center for Environmental Health, Neuherberg, Germany; ^59^ Cardiovascular and Nutritional Epidemiology, Institute of Environmental Medicine, Karolinska Institutet, Stockholm, Sweden; ^60^ Department of Population and Quantitative Health Sciences, Cleveland, OH, United States; ^61^ Department of Mathematics and Statistics, St. Cloud State University, St. Cloud, MN, United States; ^62^ Department of Clinical Chemistry, University of Tampere, Tampere, Finland; ^63^ Finnish Cardiovascular Research Center, University of Tampere, Tampere, Finland; ^64^ Unit of Genetic Epidemiology and Bioinformatics, Department of Epidemiology, University Medical Center Groningen, University of Groningen, Groningen, Netherlands; ^65^ Genetic and Molecular Epidemiology Unit, Department of Clinical Sciences, Skåne University Hospital, Lund University, Malmö, Sweden; ^66^ Kuopio Research Institute of Exercise Medicine, Kuopio, Finland; ^67^ Department of Cardiovascular Sciences, University of Leicester, Leicester, United Kingdom; ^68^ NIHR Leicester Biomedical Research Centre, Glenfield Hospital, Leicester, United Kingdom; ^69^ Institute for Maternal and Child Health-IRCCS Burlo Garofolo, Trieste, Italy; ^70^ Biostatistics, Department of Medicine, Brigham and Women’s Hospital, Harvard University, Boston, MA, United States; ^71^ Department of Gene Diagnostics and Therapeutics, Research Institute, National Center for Global Health and Medicine, Tokyo, Japan; ^72^ Department of Public Health Sciences, Loyola University Chicago, Maywood, IL, United States; ^73^ Department of Epidemiology, University Medical Center Groningen, University of Groningen, Groningen, Netherlands; ^74^ Department of Cardiology, University Medical Center Groningen, University of Groningen, Groningen, Netherlands; ^75^ Survey Research Center, Institute for Social Research, University of Michigan, Ann Arbor, MI, United States; ^76^ Interfaculty Institute for Genetics and Functional Genomics, University Medicine Greifswald and University of Greifswald, Greifswald, Germany; ^77^ German Center for Cardiovascular Research, Greifswald, Germany; ^78^ Division of Epidemiology, Department of Medicine, Vanderbilt University Medical Center, Nashville, TN, United States; ^79^ Division of General Internal Medicine, Department of Medicine, Johns Hopkins University School of Medicine, Baltimore, MD, United States; ^80^ Department of Medical Epidemiology and Biostatistics, Karolinska Institutet, Stockholm, Sweden; ^81^ Department of Genetic Medicine, McKusick-Nathans Institute, Johns Hopkins University School of Medicine, Baltimore, MD, United States; ^82^ Section of Cardiovascular Research, Baylor College of Medicine, Houston, TX, United States; ^83^ Houston Methodist Debakey Heart and Vascular Center, Houston, TX, United States; ^84^ Human Genome Sequencing Center, Baylor College of Medicine, Houston, TX, United States; ^85^ Section on Genomic Pediatrics, Department of Pediatrics, Medical College of Wisconsin, Milwaukee, WI, United States; ^86^ Centre for Genomic and Experimental Medicine, Institute of Genetics & Cancer, University of Edinburgh, Edinburgh, United Kingdom; ^87^ Usher Institute for Population Health Sciences and Informatics, University of Edinburgh, Edinburgh, United Kingdom; ^88^ Data Science Unit, Singapore Eye Research Institute, Singapore National Eye Centre, Singapore, Singapore; ^89^ Department of Clinical Epidemiology, Leiden University Medical Center, Leiden, Netherlands; ^90^ Department of Medicine, Faculty of Medicine, University of Kelaniya, Ragama, Sri Lanka; ^91^ Tropical Medicine Research Institute, University of the West Indies, Mona, Jamaica; ^92^ Braun School of Public Health, Hadassah Medical Center, Hebrew University, Jerusalem, Israel; ^93^ Section for Nutritional Research, Department of Public Health and Clinical Medicine, Umeå University, Umeå, Sweden; ^94^ Institute of Biomedicine, School of Medicine, University of Eastern Finland, Kuopio, Finland; ^95^ Department of Paediatrics, Yong Loo Lin School of Medicine, National University of Singapore, Singapore, Singapore; ^96^ Khoo Teck Puat National University Children’s Medical Institute, National University Health System, Singapore, Singapore; ^97^ Department of Internal Medicine, University of Utah, Salt Lake City, UT, United States; ^98^ Department of Genetic Medicine, Weill Cornell Medicine in Qatar, Doha, Qatar; ^99^ Division of Epidemiology and Community Health, School of Public Health, University of Minnesota, Minneapolis, MN, United States; ^100^ Novo Nordisk Foundation Center for Basic Metabolic Research, Faculty of Health and Medical Sciences, University of Copenhagen, Copenhagen, Denmark; ^101^ Department of Environmental Medicine and Public Health, The Icahn School of Medicine at Mount Sinai, New York, NY, United States; ^102^ Healthy Longevity Translational Research Programme, Yong Loo Lin School of Medicine, National University of Singapore, Singapore, Singapore; ^103^ Singapore Institute for Clinical Sciences, Agency for Science Technology and Research (A*STAR), Singapore, Singapore; ^104^ Epidemiology, Department of Preventive Medicine, Northwestern University Feinberg School of Medicine, Chicago, IL, United States; ^105^ Institute of Human Genetics, Helmholtz Zentrum München, German Research Center for Environmental Health, Neuherberg, Germany; ^106^ Institute of Human Genetics, Technische Universität München, Munich, Germany; ^107^ Psychiatry, VU Medisch Centrum, Amsterdam, Netherlands; ^108^ Institute of Clinical Chemistry and Laboratory Medicine, University Medicine Greifswald, Greifswald, Germany; ^109^ Division of Endocrinology, Diabetes, and Nutrition, University of Maryland School of Medicine, Baltimore, MD, United States; ^110^ Program for Personalized and Genomic Medicine, University of Maryland School of Medicine, Baltimore, MD, United States; ^111^ Department of Biochemistry, Wake Forest School of Medicine, Winston-Salem, NC, United States; ^112^ Geriatrics Section, Department of Medicine, Boston University School of Medicine, Boston, MA, United States; ^113^ German Center for Cardiovascular Research, Neuherberg, Germany; ^114^ Centre for Population Health Research, University of Turku and Turku University Hospital, Turku, Finland; ^115^ Research Centre of Applied and Preventive Cardiovascular Medicine, University of Turku, Turku, Finland; ^116^ Department of Clinical Physiology and Nuclear Medicine, Turku University Hospital, Turku, Finland; ^117^ Division of Research, Kaiser Permanente of Northern California, Oakland, CA, United States; ^118^ Jackson Heart Study, Department of Medicine, University of Mississippi Medical Center, Jackson, MS, United States; ^119^ Alzheimer Scotland Dementia Research Centre, The University of Edinburgh, Edinburgh, United Kingdom; ^120^ German Research Center for Environmental Health, Helmholtz Zentrum München, Institute of Genetic Epidemiology, Neuherberg, Germany; ^121^ Institute of Medical Informatics Biometry and Epidemiology, Ludwig-Maximilians-Universität München, Munich, Germany; ^122^ Yong Loo Lin School of Medicine, National University of Singapore and National University Health System, Singapore, Singapore; ^123^ Duke-National University of Singapore Medical School, Singapore, Singapore; ^124^ Department of Laboratory Medicine and Pathology, Minneapolis, MN, United States; ^125^ Department of Internal Medicine, Erasmus MC, University Medical Center, Rotterdam, Netherlands; ^126^ German Center for Cardiovascular Research (DZHK), Partner Site Munich Heart Alliance, Munich, Germany; ^127^ Beijing Ophthalmology and Visual Science Key Lab, Beijing Tongren Eye Center, Beijing Tongren Hospital, Beijing Institute of Ophthalmology, Capital Medical University, Beijing, China; ^128^ Jackson Heart Study Graduate Training Center, School of Public, Jackson State University, Jackson, MS, United States; ^129^ Department of Epidemiology, School of Public Health, University of Pittsburgh, Pittsburgh, PA, United States; ^130^ Division of Cancer Control and Population Sciences, University of Pittsburgh Medical Center (UPMC) Hillman Cancer Center, Pittsburgh, PA, United States; ^131^ Department of Metabolism, Digestion and Reproduction, Imperial College London, London, United Kingdom; ^132^ Broad Institute, Massachusetts Institute of Technology and Harvard University, Boston, MA, United States; ^133^ Department of Public Health and Clinical Medicine, Umeå University, Umeå, Sweden; ^134^ Department of Nutrition, Harvard Chan School of Public Health, Boston, MA, United States; ^135^ German Center for Diabetes Research, Neuherberg, Germany; ^136^ Department of Ophthalmology, Medical Faculty Mannheim, University Heidelberg, Mannheim, Germany; ^137^ Institute of Molecular and Clinical Ophthalmology, Basel, Switzerland; ^138^ Department of Clinical Physiology and Nuclear Medicine, Kuopio University Hospital, Kuopio, Finland; ^139^ Department of Epidemiology, University of North Carolina at Chapel Hill, Chapel Hill, NC, United States; ^140^ Celgene, Bristol Myers Squibb, Mississauga, ON, Canada; ^141^ Department of Medicine, Surgery and Health Sciences, University of Trieste, Trieste, Italy; ^142^ Division Heart and Lungs, Department of Cardiology, University Medical Center Utrecht, University of Utrecht, Utrecht, Netherlands; ^143^ Institute for Community Medicine, University Medicine Greifswald, Greifswald, Germany; ^144^ National Institute on Aging, National Institutes of Health, Bethesda, MD, United States; ^145^ Division of Cardiology, Department of Medicine, University of Mississippi Medical Center, Jackson, MS, United States; ^146^ Faculty of Medicine, University of Iceland, Reykjavik, Iceland; ^147^ Department of Public Health and Primary Care, Leiden University Medical Center, Leiden, Netherlands; ^148^ Department of Epidemiology, University of Washington, Seattle, WA, United States; ^149^ Department of Health Systems and Population Health, University of Washington, Seattle, WA, United States; ^150^ Centre for Global Health, The Usher Institute, The University of Edinburgh, Edinburgh, United Kingdom; ^151^ Division of Population Health and Genomics, Ninewells Hospital and Medical School, University of Dundee, Dundee, United Kingdom; ^152^ Department of Exercise and Nutrition Sciences, Milken Institute School of Public Health, The George Washington University, Washington, DC, United States; ^153^ Nuffield Department of Population Health, University of Oxford, Oxford, United Kingdom; ^154^ College of Public Health, Dean’s Office, University of Kentucky, Lexington, KY, United States; ^155^ Division of Biostatistics, Population and Public Health Sciences, University of Southern California, Los Angeles, CA, United States

**Keywords:** educational attainment, lipids, cholesterol, triglycerides, genome-wide association study, meta-analysis

## Abstract

**Introduction:** Educational attainment, widely used in epidemiologic studies as a surrogate for socioeconomic status, is a predictor of cardiovascular health outcomes.

**Methods:** A two-stage genome-wide meta-analysis of low-density lipoprotein cholesterol (LDL), high-density lipoprotein cholesterol (HDL), and triglyceride (TG) levels was performed while accounting for gene-educational attainment interactions in up to 226,315 individuals from five population groups. We considered two educational attainment variables: “Some College” (yes/no, for any education beyond high school) and “Graduated College” (yes/no, for completing a 4-year college degree). Genome-wide significant (*p* < 5 × 10^−8^) and suggestive (*p* < 1 × 10^−6^) variants were identified in Stage 1 (in up to 108,784 individuals) through genome-wide analysis, and those variants were followed up in Stage 2 studies (in up to 117,531 individuals).

**Results:** In combined analysis of Stages 1 and 2, we identified 18 novel lipid loci (nine for LDL, seven for HDL, and two for TG) by two degree-of-freedom (2 DF) joint tests of main and interaction effects. Four loci showed significant interaction with educational attainment. Two loci were significant only in cross-population analyses. Several loci include genes with known or suggested roles in adipose (*FOXP1, MBOAT4, SKP2, STIM1, STX4*), brain (*BRI3, FILIP1, FOXP1, LINC00290, LMTK2, MBOAT4, MYO6, SENP6, SRGAP3, STIM1, TMEM167A, TMEM30A*), and liver (*BRI3, FOXP1*) biology, highlighting the potential importance of brain-adipose-liver communication in the regulation of lipid metabolism. An investigation of the potential druggability of genes in identified loci resulted in five gene targets shown to interact with drugs approved by the Food and Drug Administration, including genes with roles in adipose and brain tissue.

**Discussion:** Genome-wide interaction analysis of educational attainment identified novel lipid loci not previously detected by analyses limited to main genetic effects.

## 1 Introduction

Educational attainment is widely used in epidemiologic studies as an index of socioeconomic status (SES) (Kaplan and Keil, 1993). Many studies have identified educational level and other indices of SES as predictors of health outcomes (Hamad et al., 2019), coronary heart disease (CHD) risk factors (Hamad et al., 2019), and lifestyle choices such as consumption of an atherogenic diet (Shea et al., 1993). Although educational level may not capture a holistic representation of SES (Braveman et al., 2005), higher educational attainment has been shown to have a positive impact on all-cause mortality (Kaplan and Keil, 1993) and cardiovascular risk traits (Leino et al., 1999) such as blood pressure and hypertension (Leng et al., 2015), coronary artery disease (Matthews et al., 1989), coronary calcification (Gallo et al., 2001), metabolic syndrome (Matthews et al., 1989), and lipid levels (Matthews et al., 1989; Metcalf et al., 1998). However, the mitigating effects of higher education on health outcomes are often attenuated in minoritized groups (Braveman et al., 2005; Assari and Bazargan, 2019), even after controlling for other indices of SES (Metcalf et al., 1998). This differential effect raises the possibility that interactions between educational attainment and genetics contribute to the association with health outcomes.

There has been relatively little focus on genetic interactions with educational attainment as determinants of health outcomes, particularly cardiovascular health, although genetic influences on education level itself (Okbay et al., 2016) have been explored. We have previously reported novel blood pressure loci by genome-wide association studies (GWAS) that explicitly modeled genetic interactions with educational attainment (Basson et al., 2014; de las Fuentes et al., 2020). Other studies have identified evidence of gene-environment interactions for a variety of disease traits including neuropsychiatric disorders (Assary et al., 2018; Werme et al., 2021), systemic lupus erythematosus, (Woo et al., 2022), and lung function (Melbourne et al., 2022).

There has been no comprehensive assessment of interactions between genetic variation and educational attainment on lipid levels. Dyslipidemia, a leading contributor to cardiovascular morbidity and mortality, exhibits significant disparity among population groups. Consideration of educational attainment as a genetic modifier may allow identification of novel lipid loci and offer insights into the biological mechanisms that may serve to identify new therapeutic targets. Here, by combining cohorts available in the Cohorts for Heart and Aging Research in Genomic Epidemiology (CHARGE) Gene-Lifestyle Interactions Working Group (Rao et al., 2017), we performed genome-wide meta-analysis of low-density lipoprotein cholesterol (LDL), high-density lipoprotein cholesterol (HDL), and triglyceride (TG) levels while accounting for gene-educational attainment interactions, used as a surrogate for socioeconomic status.

## 2 Materials and methods

### 2.1 Participating studies

We performed analyses in two stages (Supplementary Figure S1). A total of 41 cohorts including 108,784 men and women (aged 17–80 years) from European (EUR), African (AFR), East Asian (EAS), Hispanic admixed (HIS), and Brazilian admixed (BRZ) populations contributed to Stage 1 genome-wide interaction analyses (Supplementary Table S1); populations were defined by individual cohorts. An additional 42 cohorts (Supplementary Table S2) including 117,531 individuals contributed to Stage 2 analyses of most promising genetic variants [mostly single nucleotide variants (SNVs), also including a small number of insertions and deletions (indels)] selected from Stage 1. Participating studies are described in the Supplementary Material. Each study obtained informed consent from participants and approval from the appropriate institutional and/or ethical review boards.

### 2.2 Lipid and educational attainment variables

Both longitudinal and cross-sectional studies were included. In longitudinal cohorts that had multiple clinic visits for each subject, a single visit was chosen that maximized the sample size. Three lipid traits were considered for analyses: low-density lipoprotein cholesterol (LDL), high-density lipoprotein cholesterol (HDL), and triglyceride (TG) (all mg/dL). LDL was directly assayed or calculated via the Friedewald equation (LDL = TC−HDL−[TG/5]) for those with fasting TG ≤ 400 mg/dL (Friedewald et al., 1972). If fasting TG > 400 mg/dL or if TG is non-fasting, LDL was set to missing unless directly assayed. LDL concentrations were adjusted for statin use as described elsewhere (Peloso et al., 2014). Either fasting or non-fasting HDL was acceptable for analysis. Non-fasting TG levels were set to missing. HDL and TG concentrations were natural log-transformed for analysis. Descriptive statistics for these lipid traits are presented in Supplementary Tables S3, S4. For educational attainment, two dichotomous variables were defined in a way that made it possible to harmonize the variable in most cohorts, thereby maximizing the sample size. The first variable, “Some College” (SomeCol), was coded as 1 if the subject received any education beyond high school (i.e., 12 years of combined primary and secondary education), including vocational school, and as 0 if no education beyond high school. The second variable, “Graduated College” (GradCol), was coded as 1 if the subject completed at least a 4-year college degree (i.e., post-secondary or tertiary education, at least 16 years of formal education), and as 0 for any education less than a 4-year degree. Subjects with missing data for lipid levels, educational attainment, or any covariates were excluded from analysis.

### 2.3 Genotype data

Genotyping was performed by each participating study using Illumina (San Diego, CA, United States) or Affymetrix (Santa Clara, CA, United States) genotyping arrays. Imputation was performed using the 1000 Genomes Project (1000 Genomes Project Consortium et al., 2012) Phase I Integrated Release Version 3 Haplotypes (2010-11 data freeze, 2012-03-14 haplotypes) as a reference panel by most cohorts. Information on genotype platform and imputation for each study is presented in Supplementary Tables S5, S6 and as described in the Supplementary Material.

### 2.4 Analysis methods

Each study performed population-specific association analyses using the following Model 1 (joint model) that includes the effects of G, educational attainment, and their interaction (see below):

$$E\left(Y\right)=\beta_{0}+\beta_{G}\;G+\beta_{E}\text{ Education}+\beta_{\text{GE}}\;G\times \text{Education}+\beta_{C}\;C$$ (1)

where Y is the lipid variable (LDL, HDL, or TG), “Education” is the educational variable (SomeCol or GradCol), and G is the dosage of the imputed variant coded additively from 0 to 2. The vector of adjustment covariates (**C**) includes age, sex, indicators of field center (for multi-center studies), and principal components (as many as deemed necessary by study personnel to adjust for population stratification). In addition, studies in Stage 1 performed association analysis using the following Model 2 that includes the effects of G and educational attainment (but not their interaction):

$$E\left(Y\right)=\beta_{0}+\beta_{G}\;G+\beta_{E}\text{ Education}+\beta_{C}\;C$$ (2)

For model 1, each study provided the estimated variant effect (β_G_), estimated variant-educational attainment interaction effect (β_GE_), their robust standard errors, and a robust estimate of the covariance between β_G_ and β_GE_. We considered the 1 degree of freedom (DF) test of the interaction effect (β_GE_) and 2 DF joint test of both variant (β_G_) and interaction effects (β_GE_) (Kraft et al., 2007). Population-specific and cross-population inverse-variance weighted meta-analysis was performed for the 1 DF test and joint 2 DF test (Manning et al., 2011), both using METAL (Willer et al., 2010). In Stage 1, EUR, AFR, EAS meta-analyses, variants were included if they were available in more than 5,000 samples or at least 3 cohorts (these filters were not applied to BRZ or HIS because of the limited number/size of the available cohorts included in these meta-analyses). We applied genomic control correction (Devlin and Roeder, 1999) twice in Stage 1, first for study-specific GWAS results and again for meta-analysis results. Genome-wide significant (*p* < 5 × 10^−8^) and suggestive (*p* < 1 × 10^−6^) variants in Stage 1 were taken forward into Stage 2 analysis. Genomic control correction was not applied to the Stage 2 results as association testing was performed for only selected variants. Results presented reflect meta-analyses combining Stages 1 and 2. Loci were defined by physical distance (±1 Mb around the lead variant of the respective locus).

Extensive quality control was performed, as described in the Supplementary Material. For Stages 1 and 2, to remove unstable study-specific results that reflected small sample size, low minor allele count (MAC), or low imputation quality, we excluded variants for which the “approximate DF” (defined as the minimum of [MAC0, MAC1] × imputation quality) < 20, where MAC0 and MAC1 are the MAC in the two educational attainment strata per exposure variable.

### 2.5 Characterization of functional roles

Loci were characterized as known (previously reported, as defined in the Supplementary Methods) or novel. A suite of tools implemented in Functional Mapping and Annotation (FUMA) of Genome-Wide Association Studies (Watanabe et al., 2017) (version 1.3.5; described in detail in the Supplementary Material) were used to identify functional roles for the lead variants and nearby variants in linkage disequilibrium (LD; r^2^ ≥ 0.2) in each of the novel lipid loci. LD information was obtained from the 1000 Genomes Project Phase 3 reference genome for the population with the most significant population-specific association. If the most significant association was in cross-population analyses (CPA), the reference genome for “1000G Phase 3 ALL” was used (Ward and Kellis, 2012). Two lead insertion/deletion loci were not identified in the reference genomes by FUMA and therefore not detailed. Nearest gene annotations were limited to protein coding, long non-coding RNAs (lncRNAs), and non-coding RNAs (ncRNAs) within 10 kb of lead variants and variants in LD (r^2^ ≥ 0.2) with the lead variant (Wang et al., 2010).

For the lead and LD variants, we used FUMA to report the RegulomeDB score, Combined Annotation Dependent Depletion (CADD) scores, the 15-core chromatin state (ChromHMM), and expression quantitative trait loci (eQTLs). Using nearest-gene annotations, FUMA was used to generate tissue-specific gene expression data (GTEx V8 dataset, 53 tissue types).

## 3 Results

### 3.1 Overview

We performed a two-stage meta-analysis of gene-educational attainment interactions on lipid traits considering two educational attainment variables, as previously described (Supplementary Figure S1) (de las Fuentes et al., 2020) Herein, we report our findings based on up to 227,850 individuals from five populations. In Stage 1, we pursued genome-wide interrogation in 108,784 individuals of European (EUR; n = 80,379), African (AFR; n = 12,295), East Asian (EAS; n = 11,002), Hispanic/Latino admixed (HIS; n = 1,455), and Brazilian admixed (BRZ; n = 3,653) populations (Supplementary Table S1). We performed genome-wide meta-analyses of approximately 18.8 million SNVs and indels variants imputed using the 1000 Genomes Project reference panel (QQ plots, Supplementary Figures S2A–E). Through the 1 DF test of the interaction effect and the 2 DF joint test of the SNV and interaction effects, we identified 13,851 genome-wide significant (*p* < 5 × 10^−8^) and 6,835 suggestive (*p* < 1 × 10^−6^ and ≥5 × 10^−8^) variants in known or novel loci that were associated with any lipid trait in any population or educational attainment analysis. These were followed-up in 117,531 additional individuals of EUR (n = 92,690), AFR (n = 6,630), EAS (n = 6,589), and HIS (n = 11,622) populations in Stage 2 (Supplementary Table S2).

**FIGURE 1:**
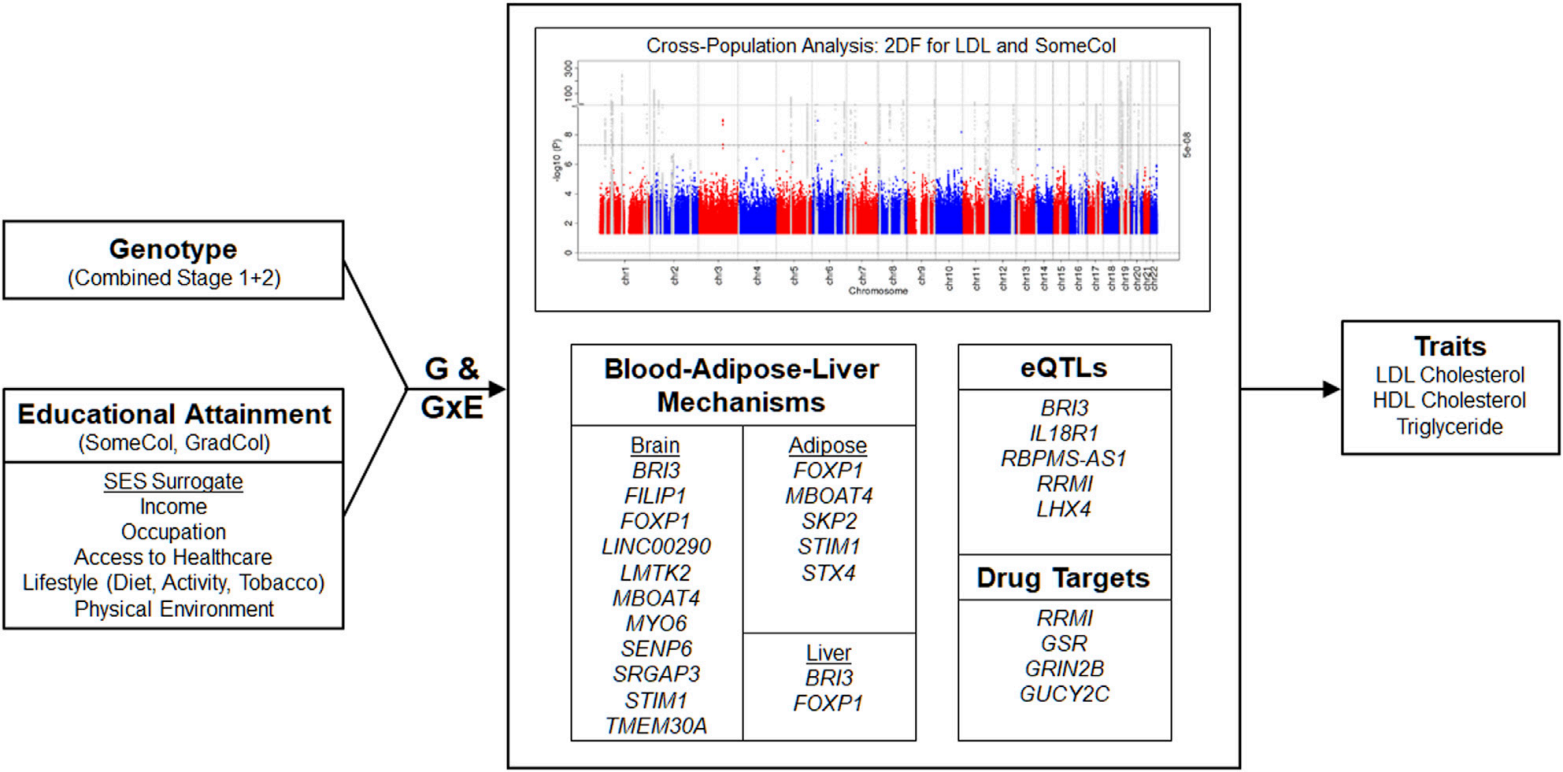
Overview. A two-stage meta-analysis of gene-educational attainment interactions on lipid traits considering two educational attainment (a surrogate for socioeconomic status) was performed. Subsequently, a meta-analysis combining results of Stages 1 and 2 was performed to identify known and novel loci for lipid traits. Identified loci include genes with known or suggested roles in brain, adipose, and liver biology. Functional annotation, expression quantitative trait loci (eQTLs), and potential druggability of targets was explored. In the Manhattan plot, known and novel loci are depicted in gray and red/blue, respectively. GxE, gene-environment interaction; GradCol, graduated college; SES, socioeconomic status; SomeCol, some college.

We then performed meta-analyses combining Stages 1 and 2 (Figure 1; Manhattan Plots; Supplementary Figures S3A–F) and identified 128 significant loci (*p* < 5 × 10^−8^): 18 were novel loci and 110 loci were known (previously reported) loci, although the specific index variant often varied based on population (Supplementary Table S7). The majority of the associations with known loci were detected for EUR (99 loci) and cross-population analyses (CPA; 107 loci), reflecting the European-centric composition of prior studies, although known loci were also detected for the other populations: AFR (20 loci), EAS (15 loci), BRZ (3 loci), and HIS (22 loci). Four LDL and five HDL known loci were only identified by CPA. Eight of the 110 known loci were significantly associated with two lipid traits.

**FIGURE 2:**
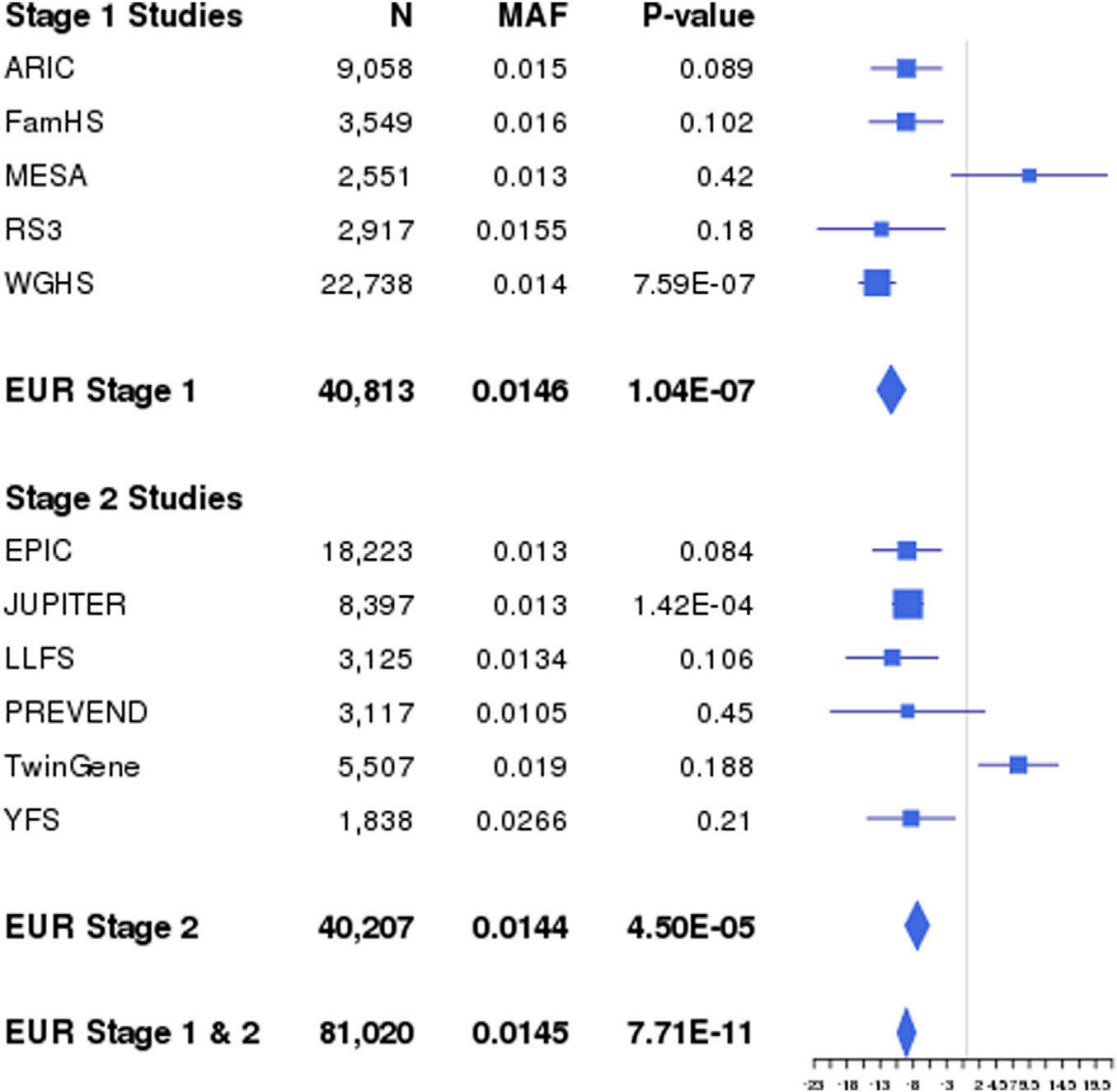
Interaction effects of Locus 15 (rs35287906; *STIM1*) identified through combined Stage 1 and Stage 2 interaction effects with GradCol for LDL in EUR and CPA. Forest plots show β values (95% confidence intervals) and *p*-values (1 DF) for the rs35287906 × GradCol interaction term in linear regression models of LDL adjusted for age, sex, field center (for multi-center studies), and principal components. Results shown are for each EUR study, as well as the population-specific combined Stage 1 and 2 meta-analysis results. The interaction effect β_G_ Educ corresponds to the difference in genetic effects between higher (β_G1_ = −5.25 mg/dL per minor allele) and lower education (β_G0_ = 3.41 mg/dL per minor allele), for a combined interaction effect of −8.66 mg/dL. AF, coded allele frequency; N, sample size.

Across the lipid fractions, educational exposures, and populations, we identified 18 novel (*p* < 5 × 10^−8^) loci located at least 1 Mb away from any known lipid loci (Table 1) using the 1 DF and/or the 2 DF tests. Of these 18 loci, seven were identified by Stage 1 analyses only as Stage 2 analyses did not meet the filter threshold of an “approximate DF” ≥ 20. Among the 18 loci, one was identified only through 1 DF interaction-effect analyses (locus 6), 14 through 2 DF analyses, and three through both 2 DF and 1 DF interaction-effect only analyses (loci 4, 12, and 15). The four loci with significant 1 DF interaction effects include lead variants in or adjacent to forkhead box P1 (*FOXP1*), long intergenic non-protein coding RNA 290 (*LINC00290*), general transcription factor IIE subunit 2 (*GTF2E2*)*-*membrane bound O-acyltransferase domain containing 4 (*MBOAT4*), and stromal interaction molecule 1 (*STIM1*). For example, at *STIM1* (locus 15), we observed an opposite genetic effect between higher and lower education: the minor allele C was associated with a 0.14 mmol/L lower LDL in higher education (GradCol = 1), whereas it was associated with a 0.09 mmol/L higher LDL in lower education (GradCol = 0), for a combined interaction effect of −0.22 mmol/L (Figure 2).

**TABLE 1:** Summary of novel loci.

Locus	rsID^a^	Chr	Pos (hg19)	Coded allele	Coded allele Freq^b^	Beta_0_ ^c^	Beta_1_ ^d^	2 DF P^e^	1 DF G × E P^f^	N	Genes^g^	RefPop-trait-Exp^h^
1	rs139845754	1	180,258 167	A	0.052	−0.101	−0.06	3.40E-08	2.00E-01	10,913	*LHX4; RP5-11 80C10.2;* ***ACBD6*** *; XPR1; KIAA1614; STX6*	AFR-TG-SomeCol
2^i^	rs7567133	2	103,239 356	C	0.031	0.102	0.021	1.72E-08	1.16E-03	6,361	*IL1RL1; IL18R1; IL18RAP; SLC9A4;* ***SLC9A2;*** *MFSD9; TMEM182*	AFR-HDL-GradCol
3	rs79367750	3	9,096 107	C	0.096	1.251	−10.279	3.99E-08	1.94E-04	8,855	***SRGAP3***	EAS-LDL-SomeCol
4^i^	rs147731578	3	70,605 665	ATTATT	0.018	−0.145	0.034	1.39E-09	3.51E-09	15,584	*MITF; FOXP1*	EUR-HDL-SomeCol
5^i^	rs74620279	4	179,620 483	C	0.025	−12.24	11.59	4.67E-08	1.39E-06	5,669	*SNORD65*	AFR-LDL-SomeCol
6	rs77249395	4	181,181 245	G	0.035	0.006	−0.026	3.17E-06	2.41E-08	134,413	*LINC00290*	TA-HDL-GradCol EUR-HDL-GradCol
7^i^	rs11132093	4	182,664 815	A	0.061	−1.65	15.84	5.04E-09	4.10E-07	2,809	*RP11-540E16.2; TENM3*	EAS-LDL-GradCol
8	rs190502162	5	37,160 808	C	0.013	9.266	−0.715	3.35E-08	4.04E-07	48,467	*SKP2; NADK2; RANBP3L; SLC1A3; NIPBL;* ***CPLANE1*** *; NUP155; WDR70; GNDF*	EUR-LDL-SomeCol
9	rs192718305	5	82,514 005	G	0.025	3.481	19.901	2.05E-08	1.60E-03	5,664	*RP11-343L5.2; TMEM167A;* ***XRCC4***	AFR-LDL-SomeCol
10^i^	rs147892694	6	76,693 587	G	0.034	−0.037	−0.087	8.61E-09	1.20E-01	8,599	*TMEM30A; FILIP1; SENP6; MYO6;* ***IMPG1***	AFR-HDL-SomeCol
11	rs7015	7	97,920 623	A	0.203	−1.142	−0.526	4.56E-08	6.18E-02	157,929	*LMTK2; TECPR1;* ***BRI3*** *; BAIAP2L1; NPTX2*	CPA-LDL-SomeCol
12^j^	rs77655002	8	30,437 023	C	0.042	−9.97	10.73	5.52E-09	7.65E-08	7,166	*MBOAT4; RBPMS-AS1; RBPMS;* ***GTF2E2*** *; GSR; TEX15*	AFR-LDL-GradCol CPA-LDL-GradCol
13	rs144190766	10	108,927 076	C	0.03	−0.015	−0.103	2.52E-08	5.64E-03	9,677	*SORCS1; RNA5SP326*	AFR-HDL-SomeCol
14	rs116562538	10	129,845 329	A	0.028	−4.573	−9.049	6.79E-09	4.82E-02	20,939	***PTPRE***	CPA-LDL-SomeCol
15	rs35287906	11	4,041 010	C	0.014	3.405	−5.247	1.53E-09	7.71E-11	81,020	*PGAP2; RHOG;* ***STIM1*** *; RRM1; OR55B1P; RP11-23F23.3*	EUR-LDL-GradCol
16	rs11230661	11	55,451 313	A	0.185	0.01	0.007	3.15E-10	3.90E-01	151,320	*TRIM48; TRIM51; 87* Olfactory Receptor Genes; *APLNR*	EUR-HDL-GradCol EUR-HDL-SomeCol
17^i^	rs148063115	12	14,221 135	T	0.026	0.022	0.222	4.86E-08	4.35E-03	6,168	*GRIN2B; RP11-72J9.1; RP11-298E10.1; RN7SL676P; GUCY2C*	AFR-TG-GradCol
18^i^	rs190746034	13	101,418 063	G	0.022	−0.002	−0.158	1.26E-08	1.06E-05	6,361	*RP11-151A6.4; TMTC4;* ***NALCN-AS1*** *; ARF4P3*	AFR-HDL-GradCol

Notes:

^a^ rsID, based on dbSNP, build 146.

^b^ Coded allele frequency.

^c^ Effect size (beta) of the unexposed group.

^d^ Effect size (beta) of the exposed group.

^e^ GWAS, 2 DF *p*-value of the significant lead SNP, for this locus.

^f^ GWAS, 1 DF, genetic-educational attainment interaction *p*-value of the significant lead SNP, for this locus.

^g^ Nearest gene of all SNVs, in LD (r2>0.2) with lead variant; if SNV, not in FUMA, gene or nearest flanking coding genes noted. Bolded genes reflect intragenic lead SNV.

^h^ The reference panel used in FUMA, to obtain functional annotations-trait-exposure; if more than one RefPop-Trait-Exp listed, the data provided is for the more significant association which is listed first.

^i^ Only significant in Stage I analyses.

^j^ 2DF *p* = 1.83E-08, 1 DF G×E *p* = 4.14E-08 in CPA-LDL-GradCol analyses.

AFR, african population; EAS, east asian population; EUR, european population; GradCol, graduated college; HDL, high-density lipoprotein cholesterol; LDL, low-density lipoprotein cholesterol; CPA, cross-population analyses; TG, triglyceride.

Among the 18 novel loci, nine were found in LDL analyses, seven in HDL analyses, and two in TG analyses; none of the novel loci reached genome-wide significance for more than one lipid trait. Considering the 17 novel loci that were significant in 2 DF tests, six loci were identified considering “Graduated College” (GradCol), 10 were identified considering “Some College” (SomeCol), and one locus was significant for both “Graduated College” and “Some College” (locus 16). Examining the 17 loci for evidence of support for the non-significant educational attainment exposure (Supplementary Table S8), nine loci had at least nominal significance (*p* < 0.05; loci 1, 2, 3, 4, 7, 8, 11, 14, and 17), four did not have at least nominal significance (*p* ≥ 0.05; loci 10, 12, 15, and 18), and three did not have data available for the other educational attainment exposure trait (loci 5, 9, and 13) because of failure to meet filter thresholds (i.e., ≥20 copies of the minor allele in the exposed group).

The LocusZoom plots of these novel loci are presented in Supplementary Figure S4.

### 3.2 Ancestry-specific and cross-population analyses

Novel loci were identified through separate analyses of AFR (nine loci), EUR (three loci), EAS (two loci), CPA (two loci), and in both EUR and CPA (two loci). Among the 18 novel loci, two loci were identified only through CPA, as none of the population-specific analyses reached genome-wide significance. For example, the SNV (rs7015, locus 11) was only nominally associated with LDL in EUR (*p* = 3.04 × 10^−4^), HIS (*p* = 9.96 × 10^−3^), EAS (*p* = 1.99 × 10^−2^), and AFR (*p* = 2.81 × 10^−2^). However, in cross-population analysis combining these four populations, the association reached genome-wide significance (*p* = 4.56 × 10^−8^). This SNV resides in the 3’ untranslated region (UTR) of an alternatively expressed transcript of brain protein I3 (*BRI3*).

### 3.3 Functional annotation and eQTL evidence

To obtain functional annotations for the lead variants and nearby variants in linkage disequilibrium (LD; r^2^ ≥ 0.2), we used FUMA (Watanabe et al., 2017). Among the 18 lead variants representing our novel loci, eight variants were intronic to coding genes, one variant was exonic to a non-coding RNA (ncRNA), one variant was intronic-ncRNA, one variant was in a 3′UTR, and five variants were intergenic; two additional variants were indels without available annotation in FUMA. Of the 1,733 annotated variants that include both the lead variants and variants in LD (r^2^ > 0.2) with available FUMA functional annotation, the majority were intergenic (64%). Among those variants annotated to gene regions (n = 619), 72% were intronic. 8.2% were exonic, and the remaining variants were in UTR and flanking regions (Supplementary Table S9).

Of the 1,769 LD variants, 25 had RegulomeDB scores better than or equal to 3a (17 in AFR, three in EUR, and five in CPA loci), suggesting at least moderate evidence for involvement in transcription regulation (Supplementary Table S9). Sixty-five variants have CADD scores ≥10, representing the top 10% of predicted deleteriousness for SNVs genome-wide (35 in AFR, 22 in EUR, three in EAS, and five in CPA loci). Eight variants in the Chromosome 11 locus including an olfactory receptor cluster have CADD scores ranging 21.2–37.0, placing them in the top 1% of predicted deleteriousness. Two additional variants are notable for high CADD scores, an exonic variant in glutathione-disulfide reductase (*GSR*; CADD score 25.5) and a variant in the *BRI3* 3′UTR region (CADD score 21.4).

The 15-core chromatin state (ChromHMM) was assessed for 127 epigenomes in the 16 lead variants available in FUMA (Supplementary Table S9). Of the lead variants, two had histone chromatin markers consistent with active or flanking active transcription start sites, and three were in regions associated with strong transcription in relevant tissues including brain, adipose tissue, and liver. Among all 1,769 LD variants, 91 had histone chromatin markers characteristic of active or flanking active transcription start sites, 218 had markers consistent with strong transcription, and 57 were in enhancer regions. Among the LD variants, those in five loci were identified as being highly significant *cis*-acting expression trait loci (eQTLs) in the GTEx V8 database: 65 variants in *BRI3* (including index rs7015 SNV in locus 11) expressed in liver [false discovery rate (FDR) *p*-values 2.27 × 10^−24^], subcutaneous adipose tissue (FDR *p*-values 9.46 × 10^−50^), and brain (FDR *p*-values range 3.75 × 10^−18^ to 5.82 × 10^−19^); eight SNVs in interleukin 18 receptor 1 (*IL18R1;* including index SNV rs7567133 in locus 2) expressed in brain (FDR *p*-values range 8.30 × 10^−20^ to 5.89 × 10^−36^) and subcutaneous adipose tissue (FDR *p*-values 4.48 × 10^−7^); one SNV in RBPMS antisense RNA 1 (*RBPMS-AS1;* locus 12) expressed in subcutaneous adipose tissue (FDR *p*-value 5.71 × 10^−28^); seven SNVs in ribonucleotide reductase catalytic subunit M1 (*RRM1;* locus 15) expressed in subcutaneous adipose tissue (FDR *p*-values 1.36 × 10^−44^), and three SNVs in LIM homeobox 4 (*LHX4;* locus 1) expressed in brain (FDR *p*-values 8.73 × 10^−10^) and subcutaneous adipose tissue (FDR *p*-values range 1.36 × 10^−9^ to 4.50 × 10^−13^).

### 3.4 Druggability targets

The potential druggability of the identified gene targets was investigated using an integrative approach as previously described (Kavousi et al., 2022). We queried high- and medium-priority candidate gene targets using the Drug-Gene Interaction database (DGIdb), which identified 17 genes annotated as clinically actionable or members of the druggable genome (Supplementary Table S10). We identified eight genes with reported drug interactions and an additional four genes with active ligand interactions in the ChEMBL database. Among these, five gene targets were shown to interact with drugs approved by the Food and Drug Administration (FDA) that have been evaluated in late-stage clinical trials using DrugBank and ClinicalTrials.gov databases (Supplementary Table S11). Among drug targets identified, *RRM1* and *GSR* are both involved in glutathione metabolism and are targets of drugs used to treat various neoplasms; glutamate ionotropic receptor NMDA type subunit 2B (*GRIN2B*) is involved in long-term neuronal potentiation; and guanylate cyclase 2C (*GUCY2C*) modulates gut cyclic GMP signaling. *GRIN2B*, which encodes a *N*-methyl *D*-aspartate (NMDA) receptor GluN2B subunit, is a target of memantine, used to treat moderate to severe dementia in patients with Alzheimer’s disease. These results suggest that there are potential drug repurposing opportunities as novel therapies for lipid management.

## 4 Discussion

### 4.1 Overview

This study reports a genome-wide meta-analysis of data from up to 226,315 individuals from five population groups. In this study, educational attainment was used as a multidimensional surrogate of SES reflective of a variety of environmental factors such as occupation, wealth, access to quality healthcare, diet, lifestyle, and physical activity. We identified 18 novel loci for LDL, HDL, and TG at genome-wide significance when accounting for gene-educational attainment interactions. The majority of novel loci (nine of 18 loci) were identified in AFR, likely reflecting a lack of population diversity in prior large-scale genome-wide studies. Many of these novel loci include genes with biologic roles in adipose, brain, and hepatic tissue.

Adipose tissue serves a critical role in sequestering circulating free fatty acids as inert triglycerides lipid droplets. Processes that limit the differentiation or subsequent function of adipocytes may contribute to abnormal lipid metabolism. Adipose tissue is also an active endocrine organ that elaborates a variety of adipokines (Ahima, 2006), such as tissue necrosis factor-alpha (TNFα), interleukins (IL)-6, IL-1, leptin, adiponectin, and others. Dysfunctional adipose tissue and pro-inflammatory adipokines can trigger ectopic deposition of fatty acids in other tissues, such as skeletal muscle and liver (Jung and Choi, 2014; Shulman, 2014), which can lead to a variety of metabolic disorders such as insulin resistance, type 2 diabetes, non-alcohol fatty liver disease, and dyslipidemia (Franssen et al., 2011; Jung and Choi, 2014). Increased hepatic fatty acid uptake stimulates synthesis of TG-rich very low-density lipoprotein (VLDL) cholesterol particles that are converted in the bloodstream to small-dense LDL particles through a process that also lowers circulating HDL (Bays et al., 2013). Whereas most of the LDL cholesterol is taken up again by the liver, a small fraction is removed from circulation by endocytosis via LDL receptors located in extrahepatic tissues, including the brain. Brain-adipose-liver communication pathways help maintain homeostasis by integrating peripheral metabolic signals; (Franssen et al., 2011; Gliozzi et al., 2021); miscommunication leads to central dysregulation and metabolic disorders (Yi and Tschop, 2012). For example, in rodents, insulin acts in the brain to enhance hepatic TG secretion via VLDL synthesis (Scherer et al., 2016). There is additional evidence to suggest that circulating plasma cholesterol concentration may play a role in neurodegeneration in susceptible individuals (Dietschy and Turley, 2001). A high-fat, high-cholesterol diet has also been associated with impaired cognition and memory (Ledreux et al., 2016) through mechanisms that may involve brain inflammation (Pistell et al., 2010).

### 4.2 Novel lipid loci include genes expressed in adipose tissue

Given the important role played by adipose tissue in regulating lipid metabolism, it is notable that two novel loci were identified that include genes with known roles in adipocyte differentiation and/or function. For example, syntaxin 6 (*STX6*; Table 1, locus 1, TG locus in AFR) has been shown to play a role in mediating insulin-stimulated translocation of the glucose transporter-4 (Glut4) in adipose tissue (Perera et al., 2003). After feeding, transgenic mice that overexpress *Glut4* in adipose tissue show reduced activity of lipoprotein lipase (Gnudi et al., 1996), the rate-limiting step for clearing plasma TG (Wang and Eckel, 2009). S-phase kinase associated protein 2 (*SKP2*; locus 8, LDL locus in EUR) plays a role in adipocyte differentiation (Okada et al., 2009); transgenic *Skp2* knock-out mice have a 50% reduction in both subcutaneous and visceral adipocyte numbers (Cooke et al., 2007).

### 4.3 Novel lipid loci include genes expressed in the brain

Nine novel lipid loci have been identified that include genes responsible for vital functions in the central nervous system. For example, the gene products of lemur tyrosine kinase 2 (*LMTK2*; locus 11, LDL locus in CPA) and myosin VI (*MYO6*; locus 10, HDL locus in AFR) both bind with kinesin-1 light chain in neurons to mediate axonal transportation of a wide variety of cargo including mitochondria and neurotransmitter-containing vesicles, and participate in glutamate receptor endocytosis on the pre-synaptic membrane (Li et al., 2020). Genes with similar functions are often clustered along chromosomes where shared regulatory domains mediate coexpression (Semon and Duret, 2006). Such may be the case for the locus on chromosome 6 (locus 10, HDL locus in AFR) where a series of genes are expressed in the hippocampus of the brain [transmembrane protein 30A (*TMEM30A*) (Xu et al., 2012), filamin A interacting protein 1 (*FILIP1*), (LoTurco and Bai, 2006), SUMO specific peptidase 6 (*SENP6*), (Loriol et al., 2013), and *MYO6* (Tamaki et al., 2008)]. The hippocampus is responsible for consolidating short-term into long-term memory; modeling of interactions with educational attainment may have facilitated the detection of this novel locus. In murine models, knock-down of *TMEM30A* by small interfering RNAs (siRNAs) reduced neurite outgrowth in the hippocampus (Xu et al., 2012). The expression of *FILIP1*, which produces a negative regulator of filamin A, is required for appropriate neocortical cell migration (LoTurco and Bai, 2006). An LDL locus in EAS (locus 3) includes SLIT-ROBO Rho GTPase activating protein 3 (*SRGAP3*), a SLIT-ROBO activating protein that guides the growth of dendritic spines on cortical neurons (Blockus and Chedotal, 2014). Limited data from a candidate-gene association study also suggest that this locus is associated with total cholesterol, HDL, and apolipoprotein A1 in Maonan Chinese (Miao et al., 2017).

*GRIN2B* (locus 17, TG locus in AFR), which encodes a *N*-methyl *D*-aspartate (NMDA) receptor subunit, is highly expressed in the hippocampus where it plays critical roles in memory consolidation. Murine models of aging show that transgenic overexpression of *Grin2b* improves learning and memory function (Cao et al., 2007). Notably, individuals with missense mutations in *GRIN2B* develop rare autosomal dominant forms of encephalopathy characterize by intellectual disability, impaired learning, and behavior phenotypes (Swanger et al., 2016; Fedele et al., 2018). Further preclinical and translational studies are warranted to determine the mechanisms by which interactions with educational attainment may modulate lipid levels in humans.

Some novel loci include several genes that are differentially expressed under a variety of conditions that may relate to altered environmental exposures in humans. For example, *MYO6* (Tamaki et al., 2008) (locus 8, LDL locus in EUR) expression is upregulated in animal models of stress; and transmembrane protein 167A (*TMEM167A*; locus 9, LDL locus in AFR) is differentially expressed in the hippocampus of depressed murine models (Zhang et al., 2018). Of additional interest is the long non-coding RNA (lncRNA) *LINC00290* (locus 6, HDL in CPA) which has been proposed as a “human-accelerated element” contributing to primate evolutionary shifts that lead to higher-order human capabilities such as complex language, advanced learning, and long-term planning (Kamm et al., 2013). Given evidence for expression in brain tissues, it is notable that the *LINC00290* locus was only identified through interaction analyses with educational attainment.

### 4.4 Novel lipid loci include genes with roles in both adipose and brain tissues

There are four additional loci containing genes that have plausible biologic roles in both adipocyte function and in the brain. For example, in locus 12 (LDL locus in AFR), *MBOAT4* has been called a “master switch” for the ghrelin system (Romero et al., 2010). Ghrelin, which is secreted by gastric endocrine cells, is made biologically active when acylated by *MBOAT4*. In addition to playing critical roles in adipogenesis, lipogenesis, and glucose homeostasis, ghrelin also stimulates food intake through actions in the brain (Pradhan et al., 2013). In locus 15 (LDL locus in EUR), the expression of stromal interaction molecule 1 (*STIM1*) negatively regulates adipocyte differentiation, impairing their ability to accumulate lipids (Graham et al., 2009). *Stim1* is also a calcium sensor that plays a critical role in the formation of dendritic spines in developing murine hippocampal cells (Kushnireva et al., 2020). In transgenic mice, overexpression of *Stim1* leads to improved contextual learning and decreased depression- and anxiety-like behaviors (Majewski et al., 2017). solute carrier family 1 member 3 (*SLC1A3*; locus 8, LDL locus in EUR) encodes a high-affinity glutamate reuptake channel in brain astrocytes that terminates excitatory neurotransmission; *Slc1a3* knock-out mice have abnormal sociability (Zhou and Danbolt, 2014). *SLC1A3* is also expressed in adipocytes, although its role in this tissue is not well understood (Krycer et al., 2017).

### 4.5 Gene with roles in adipose, brain, and liver tissues

Two loci contain genes with plausible biologic roles in adipose, brain, and hepatic tissues. In the brain, *BRI3* (locus 11, LDL locus in CPA) has been implicated in neuronal survival following ischemia/reperfusion injury (Yang et al., 2015) and may be a protective regulator against Alzheimer disease (Matsuda et al., 2009). Several SNVs in this locus are significant eQTLs for *BRI3* expression in both the liver and subcutaneous fat. A variant in *BRI3* is notable for a CADD score predictive of being deleterious. *FOXP1* (locus 4, HDL locus in EUR) is involved in adipocyte differentiation (Liu et al., 2019). In the brain, the transcription factor, FOXP1, heterodimerizes with its paralog, forkhead box P2 (FOXP2), to form a transcription factor; rare mutations in *FOXP2* have been reported in multiple cases of intellectual disability and language impairment (Bacon and Rappold, 2012). In murine models of diabetes, hepatic *FOXP1* expression, a regulator of gluconeogenic gene expression, is downregulated (Zou et al., 2015).

### 4.6 Limitations

Several limitations are inherent in the design of large-scale multi-population genome-wide association studies such as this one. First, we were unable to validate seven of the 18 novel loci (one EUR, one EAS, and five AFR), largely due to the limited number of non-EUR cohorts available in Stage 2 and variants/cohorts failing to meet quality control thresholds; these loci need further validation. Second, 16 of the 18 novel loci were notable for having minor allele frequencies <0.10 which increases the possibility for type 1 and type 2 errors. Third, the interpretation of educational attainment as a proxy for SES may vary according to gender, population, region, country, and/or birth cohort (Tyroler, 1989; Sorel et al., 1992; Kaplan and Keil, 1993; Metcalf et al., 1998) and dichotomization may fail to capture more nuanced population differences, in particular for non-minoritized populations. For example, in developing countries where diets are becoming progressively westernized, men and women from higher SES strata are at higher risk for dyslipidemia (Espirito Santo et al., 2019). Fourth, the practice of adjusting LDL concentrations for statin use is based on a method derived from a meta-analysis of largely European-population cohorts (Baigent et al., 2005) which may not be appropriate for other populations. Finally, genome-wide association studies are largely hypothesis generating in scope; findings of association warrant validation in biological systems. While we attempted to enhance potential relevance by reporting of functional annotation and druggability of candidate gene targets, biologic plausibility was extrapolated primarily from animal and *in vitro* data that may not be relevant in human lipid metabolism.

Despite these limitations, this study has multiple strengths such as a sufficiently large sample size of cohorts inclusive across the lifespan and sex and representing diverse populations, the majority which were not selected for lipid abnormalities. Furthermore, consideration of educational attainment is a novel strategy designed to enhance discovery of novel lipid loci. Whereas GWAS studies traditionally identify variants that explain only a fraction of trait variability, even loci associated with modest changes in gene expression or protein function may lay the groundwork for identifying novel drug targets and/or repurposing of existing drugs for lipid management.

### 4.7 Conclusions

In conclusion, this multi-population meta-analysis of LDL, HDL, and TG identified 18 novel loci by consideration of gene-educational attainment interactions; one locus was identified only through evidence for interaction with educational attainment. Several of the loci include genes with known or suggested roles in adipocyte, brain, and/or liver biology. While findings of gene-environment interactions have generally not yet been translated to clinical practice, the results of this study may identify novel potential therapeutic targets for lipid management, especially those involving central control of lipid metabolism.

## Data Availability

The data analyzed in this study is subject to the following licenses/restrictions: 83 US and international cohorts participated in this study, each subject to different regulations regarding sharing of data. Requests to access these datasets should be directed to LF, lfuentes@wustl.edu.
