# Supplementary material for: Gene-educational attainment interactions in a multi-population genome-wide meta-analysis identify novel lipid loci
Source: Front Genet. 2023 Nov 2;14:1235337. doi: 10.3389/fgene.2023.1235337 (PMC10651736; doi:10.3389/fgene.2023.1235337)

Supplementary Material

Table of Contents

Supplementary Notes

Supplementary Methods

Stage 1 Study Descriptions

Stage 2 Study Descriptions

Stage 1 Study Acknowledgments

Stage 2 Study Acknowledgments

Supplementary Figures

Figure S1: Flow Chart of Study Design

Figure S2A: QQ Plots of African-Ancestry Cohorts.

Figure S2B: QQ Plots of Asian-Ancestry Cohorts.

Figure S2C: QQ Plots of Brazilian-Ancestry Cohorts.

Figure S2D: QQ Plots of European-Ancestry Cohorts.

Figure S2E: QQ Plots of Hispanic-Ancestry Cohorts.

Figure S3A: Manhattan plots of the combined analyses of Stages 1 and 2 using the 2DF for LDL and GradCol by ancestry.

Figure S3B: Manhattan plots of the combined analyses of Stages 1 and 2 using the 2DF for LDL and SomeCol by ancestry.

Figure S3C: Manhattan plots of the combined analyses of Stages 1 and 2 using the 2DF for HDL and GradCol by ancestry.

Figure S3D: Manhattan plots of the combined analyses of Stages 1 and 2 using the 2DF for HDL and SomeCol by ancestry.

Figure S3E: Manhattan plots of the combined analyses of Stages 1 and 2 using the 2DF for TG and GradCol by ancestry.

Figure S3F: Manhattan plots of the combined analyses of Stages 1 and 2 using the 2DF for TG and SomeCol by ancestry.

Figure S4: LocusZoom plots for 18 novel loci (Table 1).

Supplementary Methods

Human Subjects Research Statement

Informed consent was obtained from all subjects involved in the study by their individual cohorts. The study was conducted according to the guidelines of the Declaration of Helsinki, and approved by the Institutional Review Board (or Ethics Committee) of the institutions named in the Supplementary Information.

Genotype data

Imputation software packages used included MACH,[1] Minimac,[2] IMPUTE2,[3] or BEAGLE[4].

Quality control (QC)

Each participating cohort in Stage 1 excluded variants with minor allele frequency (MAF) < 1% or mapping to sex chromosomes or mitochondria. We performed extensive QC using the R package EasyQC[5] for all cohort-specific and meta-analysis results. For Stages 1 and 2, we excluded all variants with imputation quality measure < 0.5. In addition, to remove unstable study-specific results that reflected small sample size, low minor allele count (MAC), or low imputation quality, we excluded variants for which the "approximate DF" (defined as the minimum of [MAC0, MAC1] x imputation quality) < 20, where MAC0 and MAC1 are the MAC in the two educational attainment strata per exposure variable (either GradCol = 0 and GradCol = 1 or SomeCol = 0 and SomeCol = 1). The allele frequencies provided by each cohort were compared against those from the relevant ancestry-specific 1000 Genomes reference panel. Marker names were harmonized to ensure consistency across cohorts. In addition, we visually compared summary statistics (e.g., mean, median, standard deviation, interquartile range) of all effect estimates, standard errors (SEs), and p-values. We examined SE-N plots[5] and quantile-quantile (QQ) plots to reveal issues with trait transformation or other analytical problems. Any problems encountered during QC steps were resolved through communication with the analysts from the participating studies. More detailed information about the QC steps, including major QC problems encountered and how they were resolved, are described elsewhere.[6]

Characterization of functional roles

Loci were characterized as known (previously reported)[7-32] or novel. A suite of tools implemented in FUMA GWAS[33] (version 1.3.5) were used to identify functional roles for SNVs: RegulomeDB score,[34] which reflects a summary of annotations with known and predicted regulatory elements such as DNase hypersensitivity, binding sites of transcription factors, and promoter regions; Combined Annotation Dependent Depletion (CADD)[35] scores, which predict deleteriousness of variants; the 15-core chromatin state (ChromHMM)[36] for 129 epigenomes (labeled E001-E129) to identify histone modifications consistent with epigenetic regulation of gene expression; expression quantitative trait loci (eQTLs) using the GTEx V8 database;[37] and tissue-specific gene expression data (GTEx V8 dataset, 53 tissue types) with significance determined as a Benjamini-Hochberg false discovery rate (FDR) < 0.05.

Druggability analysis

First, the Drug-Gene Interaction database (DGIdb; v4.2.0) was used to query high or medium priority educational attainment-lipid interacting genes to determine the potentially druggability of the candidate gene targets. Genes were annotated for implicated pathways and functions using the Kyoto Encyclopedia of Genes and Genomes (KEGG) database. We annotated the druggability target categories and queried all interacting drugs reported in 43 databases (BaderLabGenes, CarisMolecularIntelligence, dGene, FoundationOneGenes, GO, HingoraniCasas, HopkinsGroom,

HumanProteinAtlas, IDG, MskImpact, Oncomine, Pharos, RussLampel, Tempus, CGI, CIViC, COSMIC, CancerCommons, ChemblDrugs, ChemblInteractions, ClearityFoundationBiomarkers, ClearityFoundationClinicalTrial, DTC, DoCM, DrugBank, Ensembl, Entrez, FDA, GuideToPharmacology, JACX-CKB, MyCancerGenome, MyCancerGenomeClinicalTrial, NCI, OncoKB, PharmGKB, TALC, TEND, TTD, TdgClinicalTrial, Wikidata). Protein targets were queried for available active ligands in ChEMBL. Gene targets were queried in the druggable genome using the most recent druggable genome list established from the NIH Illuminating the Druggable Genome Project (https://github.com/druggablegenome/IDGTargets) available through the Pharos web platform. Targets were also queried for FDA-approved drugs, late-stage clinical trials, and disease indications in the DrugBank, ChEMBL, ClinicalTrials.gov databases and results provided for the top MESH and DrugBank indications and clinical trials.

References for Supplementary Methods

1. Li Y, Willer CJ, Ding J, Scheet P, Abecasis GR. MaCH: using sequence and genotype data to estimate haplotypes and unobserved genotypes. *Genet Epidemiol* 2010; **34**(8)**:** 816-834.

2. Howie B, Fuchsberger C, Stephens M, Marchini J, Abecasis GR. Fast and accurate genotype imputation in genome-wide association studies through pre-phasing. *Nat Genet* 2012; **44**(8)**:** 955-959.

3. Howie BN, Donnelly P, Marchini J. A flexible and accurate genotype imputation method for the next generation of genome-wide association studies. *PLoS Genet* 2009; **5**(6)**:** e1000529.

4. Browning BL, Browning SR. A unified approach to genotype imputation and haplotype-phase inference for large data sets of trios and unrelated individuals. *Am J Hum Genet* 2009; **84**(2)**:** 210-223.

5. Winkler TW, Day FR, Croteau-Chonka DC, Wood AR, Locke AE, Magi R *et al.* Quality control and conduct of genome-wide association meta-analyses. *Nat Protoc* 2014; **9**(5)**:** 1192-1212.

6. de las Fuentes L, Sung YJ, Noordam R, Winkler T, Feitosa MF, Schwander K *et al.* Gene-educational attainment interactions in a multi-ancestry genome-wide meta-analysis identify novel blood pressure loci. *Mol Psychiatry* 2020.

7. Aulchenko YS, Ripatti S, Lindqvist I, Boomsma D, Heid IM, Pramstaller PP *et al.* Loci influencing lipid levels and coronary heart disease risk in 16 European population cohorts. *Nat Genet* 2009; **41**(1)**:** 47-55.

8. Coram MA, Duan Q, Hoffmann TJ, Thornton T, Knowles JW, Johnson NA *et al.* Genome-wide characterization of shared and distinct genetic components that influence blood lipid levels in ethnically diverse human populations. *Am J Hum Genet* 2013; **92**(6)**:** 904-916.

9. Do R, Willer CJ, Schmidt EM, Sengupta S, Gao C, Peloso GM *et al.* Common variants associated with plasma triglycerides and risk for coronary artery disease. *Nat Genet* 2013; **45**(11)**:** 1345-1352.

10. Heid IM, Boes E, Muller M, Kollerits B, Lamina C, Coassin S *et al.* Genome-wide association analysis of high-density lipoprotein cholesterol in the population-based KORA study sheds new light on intergenic regions. *Circ Cardiovasc Genet* 2008; **1**(1)**:** 10-20.

11. Holmen OL, Zhang H, Fan Y, Hovelson DH, Schmidt EM, Zhou W *et al.* Systematic evaluation of coding variation identifies a candidate causal variant in TM6SF2 influencing total cholesterol and myocardial infarction risk. *Nat Genet* 2014; **46**(4)**:** 345-351.

12. Kathiresan S, Melander O, Guiducci C, Surti A, Burtt NP, Rieder MJ *et al.* Six new loci associated with blood low-density lipoprotein cholesterol, high-density lipoprotein cholesterol or triglycerides in humans. *Nat Genet* 2008; **40**(2)**:** 189-197.

13. Kathiresan S, Willer CJ, Peloso GM, Demissie S, Musunuru K, Schadt EE *et al.* Common variants at 30 loci contribute to polygenic dyslipidemia. *Nat Genet* 2009; **41**(1)**:** 56-65.

14. Kim YJ, Go MJ, Hu C, Hong CB, Kim YK, Lee JY *et al.* Large-scale genome-wide association studies in East Asians identify new genetic loci influencing metabolic traits. *Nat Genet* 2011; **43**(10)**:** 990-995.

15. Lanktree MB, Elbers CC, Li Y, Zhang G, Duan Q, Karczewski KJ *et al.* Genetic meta-analysis of 15,901 African Americans identifies variation in EXOC3L1 is associated with HDL concentration. *J Lipid Res* 2015; **56**(9)**:** 1781-1786.

16. Peloso GM, Auer PL, Bis JC, Voorman A, Morrison AC, Stitziel NO *et al.* Association of low-frequency and rare coding-sequence variants with blood lipids and coronary heart disease in 56,000 whites and blacks. *Am J Hum Genet* 2014; **94**(2)**:** 223-232.

17. Ridker PM, Pare G, Parker AN, Zee RY, Miletich JP, Chasman DI. Polymorphism in the CETP gene region, HDL cholesterol, and risk of future myocardial infarction: Genomewide analysis among 18 245 initially healthy women from the Women's Genome Health Study. *Circ Cardiovasc Genet* 2009; **2**(1)**:** 26-33.

18. Sabatti C, Service SK, Hartikainen AL, Pouta A, Ripatti S, Brodsky J *et al.* Genome-wide association analysis of metabolic traits in a birth cohort from a founder population. *Nat Genet* 2009; **41**(1)**:** 35-46.

19. Sandhu MS, Waterworth DM, Debenham SL, Wheeler E, Papadakis K, Zhao JH *et al.* LDL-cholesterol concentrations: a genome-wide association study. *Lancet* 2008; **371**(9611)**:** 483-491.

20. Saxena R, Voight BF, Lyssenko V, Burtt NP, de Bakker PI, Chen H *et al.* Genome-wide association analysis identifies loci for type 2 diabetes and triglyceride levels. *Science* 2007; **316**(5829)**:** 1331-1336.

21. Spracklen CN, Chen P, Kim YJ, Wang X, Cai H, Li S *et al.* Association analyses of East Asian individuals and trans-ancestry analyses with European individuals reveal new loci associated with cholesterol and triglyceride levels. *Hum Mol Genet* 2017; **26**(9)**:** 1770-1784.

22. Surakka I, Horikoshi M, Magi R, Sarin AP, Mahajan A, Lagou V *et al.* The impact of low-frequency and rare variants on lipid levels. *Nat Genet* 2015; **47**(6)**:** 589-597.

23. Tan A, Sun J, Xia N, Qin X, Hu Y, Zhang S *et al.* A genome-wide association and gene-environment interaction study for serum triglycerides levels in a healthy Chinese male population. *Hum Mol Genet* 2012; **21**(7)**:** 1658-1664.

24. Teslovich TM, Musunuru K, Smith AV, Edmondson AC, Stylianou IM, Koseki M *et al.* Biological, clinical and population relevance of 95 loci for blood lipids. *Nature* 2010; **466**(7307)**:** 707-713.

25. van Leeuwen EM, Karssen LC, Deelen J, Isaacs A, Medina-Gomez C, Mbarek H *et al.* Genome of The Netherlands population-specific imputations identify an ABCA6 variant associated with cholesterol levels. *Nat Commun* 2015; **6:** 6065.

26. Waterworth DM, Ricketts SL, Song K, Chen L, Zhao JH, Ripatti S *et al.* Genetic variants influencing circulating lipid levels and risk of coronary artery disease. *Arterioscler Thromb Vasc Biol* 2010; **30**(11)**:** 2264-2276.

27. Weissglas-Volkov D, Aguilar-Salinas CA, Nikkola E, Deere KA, Cruz-Bautista I, Arellano-Campos O *et al.* Genomic study in Mexicans identifies a new locus for triglycerides and refines European lipid loci. *J Med Genet* 2013; **50**(5)**:** 298-308.

28. Willer CJ, Sanna S, Jackson AU, Scuteri A, Bonnycastle LL, Clarke R *et al.* Newly identified loci that influence lipid concentrations and risk of coronary artery disease. *Nat Genet* 2008; **40**(2)**:** 161-169.

29. Willer CJ, Schmidt EM, Sengupta S, Peloso GM, Gustafsson S, Kanoni S *et al.* Discovery and refinement of loci associated with lipid levels. *Nat Genet* 2013; **45**(11)**:** 1274-1283.

30. Zubair N, Graff M, Luis Ambite J, Bush WS, Kichaev G, Lu Y *et al.* Fine-mapping of lipid regions in global populations discovers ethnic-specific signals and refines previously identified lipid loci. *Hum Mol Genet* 2016; **25**(24)**:** 5500-5512.

31. Klarin D, Damrauer SM, Cho K, Sun YV, Teslovich TM, Honerlaw J *et al.* Genetics of blood lipids among ~300,000 multi-ethnic participants of the Million Veteran Program. *Nat Genet* 2018; **50**(11)**:** 1514-1523.

32. Choudhury A, Brandenburg JT, Chikowore T, Sengupta D, Boua PR, Crowther NJ *et al.* Meta-analysis of sub-Saharan African studies provides insights into genetic architecture of lipid traits. *Nat Commun* 2022; **13**(1)**:** 2578.

33. Watanabe K, Taskesen E, van Bochoven A, Posthuma D. Functional mapping and annotation of genetic associations with FUMA. *Nat Commun* 2017; **8**(1)**:** 1826.

34. Boyle AP, Hong EL, Hariharan M, Cheng Y, Schaub MA, Kasowski M *et al.* Annotation of functional variation in personal genomes using RegulomeDB. *Genome Res* 2012; **22**(9)**:** 1790-1797.

35. Kircher M, Witten DM, Jain P, O'Roak BJ, Cooper GM, Shendure J. A general framework for estimating the relative pathogenicity of human genetic variants. *Nat Genet* 2014; **46**(3)**:** 310-315.

36. Roadmap Epigenomics Consortium, Kundaje A, Meuleman W, Ernst J, Bilenky M, Yen A *et al.* Integrative analysis of 111 reference human epigenomes. *Nature* 2015; **518**(7539)**:** 317-330.

37. GTEx Consortium. Genetic effects on gene expression across human tissues. *Nature* 2017; **550**(7675)**:** 204-213.

STAGE 1 STUDY DESCRIPTIONS

Brief descriptions are provided below for each of stage 1 studies, some of which are based outside the United States:

AGES (Age Gene/Environment Susceptibility Reykjavik Study): The AGES Reykjavik study originally comprised a random sample of 30,795 men and women born in 1907-1935 and living in Reykjavik in 1967. A total of 19,381 people attended, resulting in a 71% recruitment rate. The study sample was divided into six groups by birth year and birth date within month. One group was designated for longitudinal follow up and was examined in all stages; another was designated as a control group and was not included in examinations until 1991. Other groups were invited to participate in specific stages of the study. Between 2002 and 2006, the AGESReykjavik study re-examined 5,764 survivors of the original cohort who had participated before in the Reykjavik Study.

ARIC (Atherosclerosis Risk in Communities): The ARIC study is a population-based prospective cohort study of cardiovascular disease sponsored by the National Heart, Lung, and Blood Institute (NHLBI). ARIC included 15,792 individuals, predominantly European American and African American, aged 45-64 years at baseline (1987-89), chosen by probability sampling from four US communities. Cohort members completed three additional triennial follow-up examinations, a fifth exam in 2011-2013, a sixth exam in 2016-2017, and a seventh exam in 2018-2019. The ARIC study has been described in detail previously (PMC8667593; Wright JD, Folsom AR, Coresh J, et al. The ARIC (Atherosclerosis Risk In Communities) Study: JACC Focus Seminar 3/8. J Am Coll Cardiol. 2021 Jun 15;77(23):2939-2959).

Baependi Heart Study (Brazil): The Baependi Heart Study, is an ongoing family-based cohort conducted in a rural town of the state of Minas Gerais. The study has enrolled approximate 2,200 individuals (over 10% of the town's adult population) and 10-year follow up period of longitudinal data. Briefly, probands were selected at random across 11 out of the 12 census districts in Baependi. After enrolment, the proband's first-degree (parents, siblings, and offspring), second-degree (half-siblings, grandparents/grandchildren, uncles/aunts, nephews/nieces, and double cousins), and third-degree (first cousins, great uncles/aunts, and great nephews/nieces) relatives, and his/her respective spouse's relatives resident both within Baependi (municipal and rural area) and surrounding towns were invited to participate. Only individuals age 18 and older were eligible to participate in the study. The study is conducted from a clinic/office in an easily accessible sector of the town, where the questionnaires were completed. A broad range of phenotypes ranging from cardiovascular, neurocognitive, psychiatric, imaging, physiologic and several layers of endophenotypes like metabolomics and lipidomics have been collected throughout the years Details about follow-up visits and available data can be found in the cohort profile paper (PMID: 18430212). DNA samples were genotyped using the Affymetrix 6.0 genechip. After quality control, the data were prephased using SHAPEIT and imputed using IMPUTE2 based on 1000 Genomes haplotypes.

CARDIA (Coronary Artery Risk Development in Young Adults): CARDIA is a prospective multicenter study with 5,115 Caucasian and African American participants 18-30 years of age at baseline, recruited from four centers in 1985-1986. The recruitment was done from the total community in Birmingham, AL; from selected census tracts in Chicago, IL and Minneapolis, MN; and from the Kaiser Permanente health plan membership in Oakland, CA. The details of the study design for the CARDIA study have been previously published.[1] Nine examinations have been completed since initiation of the study, respectively in the years 0, 2, 5, 7, 10, 15, 20, 25, and 30. Written informed

consent was obtained from participants at each examination and all study protocols were approved by the institutional review boards of the participating institutions.

Age and race were self-reported using standardized questionnaires, as were use of cholesterol-lowering medication, and smoking status (current, former, or never). All participants were asked to fast for 12 hours before each clinic visit. Lipid measures were performed on plasma blood samples drawn from the antecubital vein and stored at −70°C until analyzed. Plasma total cholesterol, HDL-cholesterol, and triglyceride concentrations were measured using enzymatic methods;[2] HDL-cholesterol concentrations were measured after dextran-sulfate-magnesium precipitation of other lipoproteins.[3] LDL-cholesterol concentrations were estimated with the Friedewald equation for individuals with fasting triglyceride values less than 400 mg/dL.[4] The test-retest correlations for total cholesterol, HDL-cholesterol, LDL-cholesterol, and triglycerides were 0.98 to 0.99.[5]

1. Friedman GD, Cutter GR, Donahue RP, Hughes GH, Hully SB, Jacobs DR Jr., Liu K, Savage PJ. CARDIA: study design, recruitment, and some characteristics of the examined subjects. J Clin Epidemiol. 1988;41:1105–1116.

2. Warnick GR. Enzymatic methods for quantification of lipoprotein lipids. Methods Enzymol. 1986;129:101–123.

3. Warnick GR, Benderson J, Albers JJ. Dextran sulfate-Mg+2 precipitation procedure for quantitation of high-density lipoprotein cholesterol. Clin Chem. 1982;28:1379–1388.

4. Friedewald WT, Levy RI, Fredrickson DS. Estimation of the concentration of low-density lipoprotein cholesterol in plasma, without use of the preparative ultracentrifuge. Clin Chem. 1972;18:499–502.

5. Gross M, Steffes M, Jacobs DR Jr., Yu X, Lewis L, Lewis CE, Loria CM. Plasma F2-isoprostanes and coronary artery calcification: the CARDIA Study. Clin Chem. 2005;51:125–131.

CHS (Cardiovascular Health Study): CHS is a population-based cohort study of risk factors for cardiovascular disease in adults 65 years of age or older conducted across four field centers (1). The original predominantly European ancestry cohort of 5,201 persons was recruited in 1989-1990 from random samples of the Medicare eligibility lists and an additional predominately African-American cohort of 687 persons was enrolled in 1992-93 for a total sample of 5,888. Blood samples were drawn from all participants at their baseline examination and DNA was subsequently extracted from available samples. European ancestry participants were excluded from the GWAS study sample due to prevalent coronary heart disease, congestive heart failure, peripheral vascular disease, valvular heart disease, stroke, or transient ischemic attack at baseline. After QC, genotyping was successful for 3271 European ancestry and 823 African-American participants. CHS was approved by institutional review committees at each site and individuals in the present analysis gave informed consent including consent to use of genetic information for the study of cardiovascular disease.

1. Fried LP, Borhani NO, Enright P, Furberg CD, Gardin JM, Kronmal RA, et al. The Cardiovascular Health Study: design and rationale. Ann Epidemiol 1991; 1:263-76.

CROATIA-Korcula: The CROATIA-Korcula study is a family-based, cross-sectional study in the isolated island of Korcula that included 965 examinees aged 18-95. Blood samples were collected in 2007 along with many clinical and biochemical measures and lifestyle and health questionnaires.

CROATIA-Vis: The CROATIA-Vis study is a family-based, cross-sectional study in the isolated island of Vis that included 1,056 examinees aged 8-93. Blood samples were collected in 2003 and 2004 along with many clinical and biochemical measures and lifestyle and health questionnaires.

ERF (Erasmus Rucphen Family study): Erasmus Rucphen Family is a family based study that includes inhabitants of a genetically isolated community in the South-West of the Netherlands, studied as part of the Genetic Research in Isolated Population (GRIP) program.[1,2] The goal of the study is to identify the risk factors in the development of complex disorders. Study population includes approximately 3,000 individuals who are living descendants of 22 couples who lived in the isolate between 1850 and 1900 and had at least six children baptized in the community church. All data were collected between 2002 and 2005. All participants gave informed consent, and the Medical Ethics Committee of the Erasmus University Medical Centre approved the study.

1. Pardo, Luba M., et al. "The effect of genetic drift in a young genetically isolated population." Annals of human genetics 69.3 (2005): 288-295.

2. Aulchenko, Yurii S., et al. "Linkage disequilibrium in young genetically isolated Dutch population." European Journal of Human Genetics 12.7 (2004): 527.

FamHS (Family Heart Study): The NHLBI FamHS study design, collection of phenotypes and covariates as well as clinical examination have been previously described (https://dsgweb.wustl.edu/fhscc/; PMID: 8651220). In brief, the FamHS recruited 1,200 families (approximately 6,000 individuals), half randomly sampled, and half selected because of an excess of coronary heart disease (CHD) or risk factor abnormalities as compared with age- and sex-specific population rates. The participants were sampled from four population-based parent studies: the Framingham Heart Study, the Utah Family Tree Study, and two centers for the Atherosclerosis Risk in Communities study (ARIC: Minneapolis, and Forsyth County, NC). These individuals attended a clinic exam (1994-1996) and a broad range of phenotypes were assessed in the general domains of CHD, atherosclerosis, cardiac and vascular function, inflammation and hemostasis, lipids and lipoproteins, blood pressure, diabetes and insulin resistance, pulmonary function, diet, education, socioeconomic status, habitual behavior, physical activity, anthropometry, medical history and medication use. Approximately 8 years later, study participants belonging to the largest pedigrees were invited for a second clinical exam (2002-04). The most important CHD risk factors were measured again, including lipids, parameters of glucose metabolism, blood pressure, anthropometry, and several biochemical and hematologic markers. In addition, a computed tomography examination provided measures of coronary and aortic calcification, and abdominal and liver fat burden. Medical history and medication use was updated. A total of 2,756 European ancestry subjects in 510 extended random and high CHD risk families were studied. Also, 633 African ancestry subjects were recruited at ARIC field center at the University of Alabama in Birmingham. Informed consent was obtained from all participants.

FHS (Framingham Heart Study): FHS began in 1948 with the recruitment of an original cohort of 5,209 men and women (mean age 44 years; 55 percent women). In 1971 a second generation of study participants was enrolled; this cohort (mean age 37 years; 52% women) consisted of 5,124 children and spouses of children of the original cohort. A third generation cohort of 4,095 children of offspring cohort participants (mean age 40 years; 53 percent women) was enrolled in 2002-2005 and are seen every 4 to 8 years. Details of study designs for the three cohorts are summarized elsewhere. At each clinic visit, a medical history was obtained with a focus on cardiovascular content, and participants underwent a physical examination including measurement of height and weight from which BMI was

calculated. For this study, lipid measurements were used from the first exam of the 2nd generation (1971-1975) and the 3rd generation (2002-2005) cohorts. Fasting levels of total cholesterol, high-density lipoprotein cholesterol, and triglycerides were measured using standard enzymatic methods in accordance with LRC protocols. LDL cholesterol was calculated using the Friedewald formula.

GENOA (Genetic Epidemiology Network of Arteriopathy): GENOA is one of four networks in the NHLBI Family-Blood Pressure Program (FBPP). [The FBPP Investigators, 2002; Daniels et al., 2004] GENOA's long-term objective is to elucidate the genetics of target organ complications of hypertension, including both atherosclerotic and arteriosclerotic complications involving the heart, brain, kidneys, and peripheral arteries. The longitudinal GENOA Study recruited European-American and African-American sibships with at least 2 individuals with clinically diagnosed essential hypertension before age 60 years. All other members of the sibship were invited to participate regardless of their hypertension status. Participants were diagnosed with hypertension if they had either 1) a previous clinical diagnosis of hypertension by a physician with current anti-hypertensive treatment, or 2) an average systolic blood pressure ≥ 140 mm Hg or diastolic blood pressure ≥ 90 mm Hg based on the second and third readings at the time of their clinic visit. Exclusion criteria were secondary hypertension, alcoholism or drug abuse, pregnancy, insulin-dependent diabetes mellitus, or active malignancy. During the first exam (1995-2000), 1,583 European Americans from Rochester, MN and 1,854 African Americans from Jackson, MS were examined. Between 2000 and 2005, 1,241 of the European Americans and 1,482 of the African Americans returned for a second examination. Because African-American probands for GENOA were recruited through the Atherosclerosis Risk in Communities (ARIC) Jackson field center participants, we excluded ARIC participants from analyses.

1. The FBPP Investigators. Multi-center genetic study of hypertension: The Family Blood Pressure Program (FBPP). Hypertension 2002;39:3-9. PubMed PMID: 11799070.

2. Daniels PR, Kardia SL, Hanis CL, Brown CA, Hutchinson R, Boerwinkle E, Turner ST; Genetic Epidemiology Network of Arteriopathy study. Familial aggregation of hypertension treatment and control in the Genetic Epidemiology Network of Arteriopathy (GENOA) study. Am J Med. 2004;116:676-81. PubMed PMID: 15121494.

GS:SFHS (Generation Scotland: Scottish Family Health Study): The GS:SFHS (www.generationscotland.org) is a family-based genetic epidemiology cohort with DNA, other biological samples (serum, urine and cryopreserved whole blood) and socio-demographic and clinical data from approximately 24,000 volunteers, aged 18-98 years, in ~7,000 family groups. An important feature of GS:SFHS is the breadth of phenotype information, including detailed data on cognitive function, personality traits and mental health. Although data collection was cross-sectional, GS:SFHS becomes a longitudinal cohort as a result of the ability to link to routine NHS data, using the community health index (CHI) number.

GOLDN (Genetics of Diet and Lipid Lowering Network): GOLDN is a multi-center family pharmacogenetic study that is investigating gene- environment interactions on lipid profiles. 1,200 subjects in extended pedigrees were measured before and after two environmental exposures: 1) a dietary fat challenge to assess genetic regulators of fat uptake and clearance and 2) a 3-week clinical trial of fenofibrate to assess pharmacogenetic influences on response to treatment. The goals of the study are to identify and characterize genetic loci that predict the lipid profile treatment responses. https://dsgweb.wustl.edu/PROJECTS/MP5.html

HANDLS (Healthy Aging in Neighborhoods of Diversity across the Life Span): HANDLS is a community-based, longitudinal epidemiologic study examining the influences of race and socioeconomic status (SES) on the development of age-related health disparities among a sample of socioeconomically diverse African Americans and whites. This unique study will assess over a 20-year period physical parameters and also evaluate genetic, biologic, demographic, and psychosocial, parameters of African American and white participants in higher and lower SES to understand the driving factors behind persistent black-white health disparities in overall longevity, cardiovascular disease, and cognitive decline. The study recruited 3,722 participants from Baltimore, MD with a mean age of 47.7 years, 2,200 African Americans and 1,522 whites, with 41% reporting household incomes below the 125% poverty delimiter.

Genotyping was done on a subset of self-reporting African American participants by the Laboratory of Neurogenetics, National Institute on Aging, National Institutes of Health (NIH). A larger genotyping effort included a small subset of self-reporting European ancestry samples. This research was supported by the Intramural Research Program of the NIH, NIA and the National Center on Minority Health and Health Disparities.

Health ABC (Health, Aging, and Body Composition): Cohort description: The Health ABC study is a prospective cohort study investigating the associations between body composition, weight-related health conditions, and incident functional limitation in older adults. Health ABC enrolled well-functioning, community-dwelling black (n=1281) and white (n=1794) men and women aged 70-79 years between April 1997 and June 1998. Participants were recruited from a random sample of white and all black Medicare eligible residents in the Pittsburgh, PA, and Memphis, TN, metropolitan areas. Participants have undergone annual exams and semi-annual phone interviews. The current study sample consists of 1559 white participants who attended the second exam in 1998-1999 with available genotyping data.

Genotyping: Genotyping was performed by the Center for Inherited Disease Research (CIDR) using the Illumina Human1M-Duo BeadChip system. Samples were excluded from the dataset for the reasons of sample failure, genotypic sex mismatch, and first-degree relative of an included individual based on genotype data. Genotyping was successful in 1663 Caucasians. Analysis was restricted to SNPs with minor allele frequency $\geq$ 1%, call rate $\geq$97% and HWE p$\geq$10-6. Genotypes were available on 914,263 high quality SNPs for imputation based on the HapMap CEU (release 22, build 36) using the MACH software (version 1.0.16). A total of 2,543,888 imputed SNPs were analyzed for association with vitamin D levels.

Association analysis: Linear regression models were used to generate cohort-specific residuals of naturally log transformed vitamin D levels adjusted for age, sex, BMI and season defined as summer (June-August), fall (September-November), winter (December to February) and spring (March to May) standardized to have mean 0 and variance of 1. Association between the additively coded SNP genotypes and the vitamin D residuals standardized was assessed using linear regression models. For imputed SNPs, expected number of minor alleles (i.e. dosage) was used in assessing association with the vitamin D residuals.

HERITAGE (Health, Risk Factors, Exercise Training and Genetics): The HERITAGE is the only known family-based study of exercise intervention to evaluate the role of genes and sequence variants involved in the response to a physically active lifestyle. The current study is based on the data collected at baseline of the study from 99 White families (244 males, 255 females). All subjects were required

to be sedentary and free of chronic diseases at baseline. There are over 18 trait domains (e.g. dietary, lipids and lipoproteins, glucose and insulin metabolism [fasting and IVGTT], steroids, body composition and body fat distribution, cardiorespiratory fitness), for a grand total of over one thousand variables. Moreover, most of the outcome traits were measured twice on two separate days both at baseline and after exercise training was completed. Marker data include a genome-wide linkage scan and GWAS, in addition to a large number of candidate genes.

HyperGEN (Hypertension Genetic Epidemiology Network): HyperGEN is a family-based study that looks at the genetic causes of hypertension and related conditions in EA and AA subjects. HyperGEN recruited hypertensive sibships, along with their normotensive adult offspring, and an age-matched random sample. HyperGEN has collected data on 2,471 Caucasian-American subjects and 2,300 African-American subjects, from five field centers in Alabama, Massachusetts, Minnesota, North Carolina, and Utah.

JHS (Jackson Heart Study): The Jackson Heart Study is a longitudinal, community-based observational cohort study investigating the role of environmental and genetic factors in the development of cardiovascular disease in African Americans. Between 2000 and 2004, a total of 5,306 participants were recruited from a tri-county area (Hinds, Madison, and Rankin Counties) that encompasses Jackson, MS. Details of the design and recruitment for the Jackson Heart Study cohort has been previously published.1-3 Briefly, approximately 30% of participants were former members of the Atherosclerosis Risk in Communities (ARIC) study. The remainder were recruited by either 1) random selection from the Accudata list, 2) commercial listing, 3) a constrained volunteer sample, in which recruitment was distributed among defined demographic cells in proportions designed to mirror those in the overall population, or through the Jackson Heart Study Family Study.

1. Wyatt SB, Diekelmann N, Henderson F, Andrew ME, Billingsley G, Felder SH et al. A community-driven model of research participation: the Jackson Heart Study Participant Recruitment and Retention Study. Ethn Dis 2003; 13(4):438-455.

2. Taylor HA, Jr., Wilson JG, Jones DW, et al. Toward resolution of cardiovascular health disparities in African Americans: design and methods of the Jackson Heart Study. Ethn Dis 2005; 15:S6-17.

3. Fuqua SR, Wyatt SB, Andrew ME, et al. Recruiting African-American research participation in the Jackson Heart Study: methods, response rates, and sample description. Ethn Dis 2005; 15:S6-29.

MESA (Multi-Ethnic Study of Atherosclerosis): The Multi-Ethnic Study of Atherosclerosis (MESA) is a study of the characteristics of subclinical cardiovascular disease and the risk factors that predict progression to clinically overt cardiovascular disease or progression of the subclinical disease. MESA consisted of a diverse, population-based sample of an initial 6,814 asymptomatic men and women aged 45-84. 38 percent of the recruited participants were white, 28 percent African American, 22 percent Hispanic, and 12 percent Asian, predominantly of Chinese descent. Participants were recruited from six field centers across the United States: Wake Forest University, Columbia University, Johns Hopkins University, University of Minnesota, Northwestern University and University of California - Los Angeles. Participants are being followed for identification and characterization of cardiovascular disease events, including acute myocardial infarction and other forms of coronary heart disease (CHD), stroke, and congestive heart failure; for cardiovascular disease interventions; and for mortality. The first examination took place over two years, from July 2000 - July 2002. It was followed

by four examination periods that were 17-20 months in length. Participants have been contacted every 9 to 12 months throughout the study to assess clinical morbidity and mortality.

1. Bild DE, Bluemke DA, Burke GL, Detrano R, Diez Roux AV, Folsom AR, Greenland P, Jacob DR Jr, Kronmal R, Liu K, Nelson JC, O'Leary D, Saad MF, Shea S, Szklo M, Tracy RP. Multi-ethnic study of atherosclerosis: objectives and design. Am J Epidemiol. 2002 Nov 1;156(9):871-81. PubMed PMID: 12397006.

NEO (The Netherlands Epidemiology of Obesity study): The NEO was designed for extensive phenotyping to investigate pathways that lead to obesity-related diseases. The NEO study is a population-based, prospective cohort study that includes 6,671 individuals aged 45–65 years, with an oversampling of individuals with overweight or obesity. At baseline, information on demography, lifestyle, and medical history have been collected by questionnaires. In addition, samples of 24-h urine, fasting and postprandial blood plasma and serum, and DNA were collected. Genotyping was performed using the Illumina HumanCoreExome chip, which was subsequently imputed to the 1000 genome reference panel. Participants underwent an extensive physical examination, including anthropometry, electrocardiography, spirometry, and measurement of the carotid artery intima-media thickness by ultrasonography. In random subsamples of participants, magnetic resonance imaging of abdominal fat, pulse wave velocity of the aorta, heart, and brain, magnetic resonance spectroscopy of the liver, indirect calorimetry, dual energy X-ray absorptiometry, or accelerometry measurements were performed. The collection of data started in September 2008 and completed at the end of September 2012. Participants are currently being followed for the incidence of obesity-related diseases and mortality.

Pelotas Birth Cohort Study (The 1982 Pelotas Birth Cohort Study, Brazil): The maternity hospitals in Pelotas, a southern Brazilian city (current population ~330,000), were visited daily in the year of 1982. The 5,914 liveborns whose families lived in the urban area were examined and their mothers interviewed. Information was obtained for more than 99% of the livebirths. These subjects have been followed-up at the following mean ages: 11.3 months (all children born from January to Abril 1982; n=1457), 19.4 months (entire cohort; n=4934), 43.1 months (entire cohort; n=4742), 13.1 years (random subsample; n=715), 14.7 years (systematic subsample; n=1076); 18.2 (male cohorts attending to compulsory Army recruitment examination; n=2250), 18.9 (systematic subsample; n=1031), 22.8 years (entire cohort; n=4297) and 30.2 years (entire cohort; n=3701). Details about follow-up visits and available data can be found in the two Cohort Profile papers (PMID: 16373375 and 25733577). DNA samples (collected at the mean age of 22.8 years) were genotyped for ~2.5 million of SNPs using the Illumina HumanOmni2.5-8v1 array (which includes autosomal, X and Y chromosomes, and mitochondrial variants). After quality control, the data were prephased using SHAPEIT and imputed using IMPUTE2 based on 1000 Genomes haplotypes.

RS (Rotterdam Study): The Rotterdam Study is a prospective, population-based cohort study among individuals living in the well-defined Ommoord district in the city of Rotterdam in The Netherlands.[1] The aim of the study is to determine the occurrence of cardiovascular, neurological, ophthalmic, endocrine, hepatic, respiratory, and psychiatric diseases in elderly people. The cohort was initially defined in 1990 among 7,983 persons, aged 55 years and older, who underwent a home interview and extensive physical examination at the baseline and during follow-up rounds every 3-4 years (RS-I). Cohort was extended in 2000/2001 (RS-II, 3,011 individuals aged 55 years and older) and 2006/2008 (RS-III, 3,932 subjects, aged 45 and older; since 2016, the cohort was expanded to include persons aged 40 years and older).[1] The Rotterdam Study has been approved by the Medical Ethics Committee of the Erasmus MC (registration number MEC 02.1015) and by the Dutch Ministry of Health, Welfare

and Sport (Population Screening Act WBO, license number 1071272-159521-PG). The Rotterdam Study Personal Registration Data collection is filed with the Erasmus MC Data Protection Officer under registration number EMC1712001. The Rotterdam Study has been entered into the Netherlands National Trial Register (NTR; www.trialregister.nl) and into the WHO International Clinical Trials Registry Platform (ICTRP; www.who.int/ictrp/network/primary/en/) under shared catalogue number NTR6831. All participants provided written informed consent to participate in the study and to have their information obtained from treating physicians.

1. Ikram MA, Brusselle G, Ghanbari M, *et al*. Objectives, design and main findings until 2020 from the Rotterdam Study. Eur J Epidemiol 2020; 35: 483–517. https://doi.org/10.1007/s10654-020-00640-5

SCES (The Singapore Chinese Eye Study) is a population-based, cross-sectional study of Chinese adults residing in the southwestern part of Singapore, with a focus on studying major eye diseases, including diabetic retinopathy, age-related macular degeneration, glaucoma, refractive errors and cataract. The Ministry of Home Affairs provided an initial computer generated list ethnic Chinese names of adults aged 40–80+ years of age. A final sampling frame of 6350 ethnic Chinese residents was derived from this list using an age-stratified random sampling strategy similar to SiMES and SINDI. The ongoing SCES began in February 2009 with an aim to recruit 3300 (75% response rate) participants. PMID: 19995197

SCHS-CHD (Singapore Chinese Health Study - Coronary Heart Disease): SCHS-CHD is a case-control study of coronary heart disease that was nested within the Singapore Chinese Health Study (SCHS), a prospective cohort study of 63,257 Singaporean Chinese men and women aged 45-74 years living in Singapore. We selected cases and controls from participants that provided blood samples and were free of coronary heart disease and stroke at the time of blood collection (N=24,454). Cases (N=760) had acute myocardial infarction (AMI) or died of coronary heart disease. AMI was identified through the Singapore Myocardial Infarction Registry or through the nationwide hospital discharge database followed by confirmation of AMI by cardiologists' review of medical records using the Multi-Ethnic Study of Atherosclerosis criteria (available at: http://www.mesa-nhlbi.org/manuals.aspx). Coronary heart disease deaths were identified through the Singapore Registry of Births and Deaths (ICD9 410-414 as first stated cause of death). Matched controls (N=1,491) were selected using a risk-set sampling strategy. Controls were participants who were alive and free of coronary heart disease at the time of the diagnosis or death of the index cases and were matched for age, sex, dialect group, year of recruitment and date of blood collection. In-person interviews and phlebotomy were conducted before the onset of disease and non-fasting venous blood was stored at -80^{0}C for extraction of DNA and blood biochemistry.

SiMES (Singapore Malay Eye Study): SiMES is a population-based, cross-sectional study of 3280 Malay adults aged 40–79 years, with a focus on studying major eye diseases, including diabetic retinopathy, age-related macular degeneration, glaucoma, refractive errors and cataract. An age-stratified random sampling of all Malay adults, aged 40–80 years, residing in 15 residential districts in the southwestern part of Singapore was drawn from the computer-generated random list of 16069 Malay names provided by the Ministry of Home Affairs. A total of 1400 names from each decade of age (40–49, 50–59, 60–69 and 70–79 years), or 5600 names, were selected. Of these, 4168 individuals (74.4%) were determined to be eligible to participate. A person was considered ineligible if he or she had moved from the residential address, had not lived there in the past 6 months, was deceased, or was terminally ill. Of the 4168 eligible individuals, 3280 participants (78.7%) took part in the study. The study was conducted from August 2004 to June 2006. PMID: 17365815.

SINDI (Singapore Indian Eye Study): SINDI is a population-based, cross-sectional epidemiological study of ethnic Indian adults aged between 40 and 80+ years residing in Singapore, with a focus on studying major eye diseases, including diabetic retinopathy, age-related macular degeneration, glaucoma, refractive errors and cataract. The Ministry of Home Affairs provided an initial computer-generated list of Indian names derived from a simple random sampling of all ethnic Indian adults aged 40–80+ years of age residing in 15 residential districts in southwestern Singapore. From this list, a final sampling frame of 6350 ethnic Indian residents was derived using an age-stratified random sampling strategy similar to SiMES. SINDI was conducted from March 2007 to December 2009 and recruited 3400 (75% response rate) participants. PMID: 19995197

SP2-1M / SP2-610 (Singapore Prospective Study Program): The SP2 (Singapore Prospective Study Program) is a population-based study of diabetes and cardiovascular disease in Singapore. It first surveyed subjects (Chinese, Malay and Indian) from four cross-sectional studies that were conducted in Singapore between 1982 and 1998. Subjects were between the ages of 24-95 years and represented a random sample of the Singapore population. Subjects were re-visited between 2003 and 2007. Among the 10,747 individuals who were eligible, 5,157 subjects completed a questionnaire and the subsequent clinical examinations. Of the 5,517 subjects, 2,434 Chinese were genotyped on a combination of Illumina 610, 1M and 550 arrays. Fasting HDL-C, TC and TG were measured by an automated analyzer autoanalyzer (ADVIA 2400, Bayer Diagnostics). LDL-C was calculated from Friedewald formula. Data from this revisit were utilized for this study.

1. Nang EE, Khoo CM, Tai ES, Lim SC, Tavintharan S, Wong TY, Heng D, Lee J. Is there a clear threshold for fasting plasma glucose that differentiates between those with and without neuropathy and chronic kidney disease?: the Singapore Prospective Study Program. Am J Epidemiol. 2009 Jun 15;169(12):1454-62. doi: 10.1093/aje/kwp076. Epub 2009 Apr 30. PMID: 19406920.

2. Tan KHX, Tan LWL, Sim X, Tai ES, Lee JJ, Chia KS, van Dam RM. Cohort Profile: The Singapore Multi-Ethnic Cohort (MEC) study. Int J Epidemiol. 2018 Jun 1;47(3):699-699j. doi: 10.1093/ije/dyy014. PMID: 29452397.

WGHS (Women's Genome Health Study): WGHS is a prospective cohort of female North American health care professionals representing participants in the Women's Health Study (WHS) trial who provided a blood sample at baseline and consent for blood-based analyses [PMID: 18070814]. Participants in the WHS were 45 years or older at enrollment and free of cardiovascular disease, cancer or other major chronic illness. The current data are derived from 23,294 WGHS participants for whom whole genome genotype information was available at the time of analysis and for whom self-reported European ancestry could be confirmed by multidimensional scaling analysis of 1,443 ancestry informative markers in PLINK v. 1.06. At baseline, BP and lifestyle habits related to smoking, consumption of alcohol, and physical activity as well as other general clinical information were ascertained by a self-reported questionnaire, an approach that has been validated in the WGHS demographic, namely female health care professionals.

STAGE 2 STUDY DESCRIPTIONS

Brief descriptions are provided below for each of stage 2 studies/cohorts:

AA-DHS (African American Diabetes Heart Study): AA-DHS objectives are to improve understanding of ethnic differences in coronary artery calcification and calcified plaque in populations of African and European ancestry. The AA-DHS consists of self-reported African Americans with T2D recruited from two Wake Forest School of Medicine (WFSM) studies: the family-based Diabetes Heart Study (DHS) and unrelated individuals in the AA-DHS. DHS is a cross-sectional study of European American and African American families with siblings concordant for T2D. AA-DHS started after DHS and enrolled unrelated African Americans. The AA-DHS GWAS utilized the Illumina 5M chip with imputation to 1,000 Genomes.

BES (Beijing Eye Study): The Beijing Eye Study is a population-based study that assesses the associated and risk factors of ocular and general diseases in a Chinese population. The study was initialized in 2001 and collected data from 4439 subjects aged ≥ 40 years and living in seven communities in the Beijing area. Three of these communities were located in a rural district and four were located in an urban district. The BES was followed-up in 2006, with 3251 of the original subjects participating, and in 2011, with 2695 subjects returning for the follow-up examination. At the examinations in 2006 and 2011, trained research staffs asked the subjects questions from a standard questionnaire providing information on the family status, level of education, income, quality of life, psychic depression, physical activity, and known major systemic diseases. Fasting blood samples were taken for measurement of concentrations of substances such as blood lipids, glucose, and glycosylated hemoglobin. Individuals were classified as self-reported non-smokers or self-reported current smokers. Alcohol consumption habits based on number of drinks per day were collected. Physical activity was assessed in questions on the number of hours per day and number of days per week spent on intensively or moderately performed sport activities, spent on walking, on riding a bicycle, and spent on sitting. All variables used in analyses were taken from examinations in 2006 or in 2011. The BES subjects were genotyped on two arrays, Illumina Human610-Quad (N = 832) and Illumina OmniExpress (N = 814).

BRIGHT (British Genetics of Hypertension): Participants of the BRIGHT Study are recruited from the Medical Research Council General Practice Framework and other primary care practices in the UK. Each case had a history of hypertension diagnosed prior to 60 years of age with confirmed blood pressure recordings corresponding to seated levels >150/100mmHg (1 reading) or mean of 3 readings >145/95 mmHg. BRIGHT is focused on recruitment of hypertensive individuals with BMI<30. Sample selection for GWAS was based on DNA availability and quantity.

1. Caulfield M, Munroe P, Pembroke J, Samani N, Dominiczak A, Brown M, Benjamin N, Webster J, Ratcliffe P, O'Shea S, Papp J, Taylor E, Dobson R, Knight J, Newhouse S, Hooper J, Lee W, Brain N, Clayton D, Lathrop GM, Farrall M, Connell J, MRC British Genetics of Hypertension Study. Genome-wide mapping of human loci for essential hypertension. Lancet. 2003;361:2118-23.

DESIR (Data from an Epidemiological Study on the Insulin Resistance): The DESIR cohort study aims to: describe and understand the relations between the abnormalities of the syndrome, their evolution, according to age and sex; search for risk factors of insulin resistance, in particular factors associated with the environment, lifestyle and genetic markers; quantify the links between the

syndrome and both cardiovascular disease and diabetes; evaluate the frequency of the syndrome in terms of its consequences on public health.

This work was supported by grants from the French National Research Agency (ANR-10-LABX-46 (European Genomics Institute for Diabetes) and ANR-10-EQPX-07-01 (LIGAN-PM)), and from the National Center for Precision Diabetic Medicine - PreciDIAB, which is jointly supported by the French National Agency for Research (ANR-18-IBHU-0001), by the European Union (FEDER), by the Hauts-de-France Regional Council and by the European Metropolis of Lille (MEL).

DFTJ (Dongfeng-Tongji Cohort Study): The DFTJ-cohort study includes 27,009 retired employees from a state-owned automobile enterprise in China. This study was launched in 2008 and will be followed up every 5 years. In 2013 we conducted the first follow-up. By using semi-structural questionnaire and health examination, those having cancer or severe diseases were excluded. Fasting blood samples and detailed epidemiology data were collected. The main goal of the cohort was to identify the environmental and genetic risk factors and the gene-environment interactions on chronic diseases, and to find novel biomarkers for chronic disease and mortality prediction. Finally, 1,461 included in the present study with GWAS data. All of the participants wrote informed consent and the ethical committees in the Tongji Medical College approved this research project. Detailed information has been described in elsewhere(1).

<u>QC criteria and imputation methods:</u>

We did the GWAS scan on the DFTJ-cohort with Affymetrix Genome-Wide Human SNP Array 6.0 chips. In total, we genotyped 906,703 SNPs among 1,461 subjects. After stringent QC filtering, SNPs with MAF < 0.01, Hardy-Weinberg Equilibrium (HWE) < 0.0001, and SNP call rate < 95% were excluded. Individuals with call rates < 95% were also not included for further analysis. In total, we retained 1,452 subjects with 658,288 autosomal SNPs for statistical analyses, with an overall call rate of 99.68%. We used MACH 1.0 software to impute untyped SNPs using the LD information from the HapMap phase II database (CHB+JPT as a reference set (2007-08_rel22, released 2007-03-02). Imputed SNPs with high genotype information content (Rsq > 0.3 for MACH) were kept for the further association analysis.

1. Wang, F., Zhu, J., Yao, P., Li, X., He, M., Liu, Y., Yuan, J., Chen, W., Zhou, L., Min, X. et al. (2012) Cohort profile: The Dongfeng-Tongji cohort study of retired workers. International journal of epidemiology.

DHS (Diabetes Heart Study): The Diabetes Heart Study (DHS) is an ongoing family-based cohort study investigating the epidemiology and genetics of cardiovascular disease (CVD) in a population-based sample. The DHS recruited T2D-affected siblings without advanced renal insufficiency from 1998 through 2005 in western North Carolina. DHS has collected genetic data on 1,220 self-described European American (EA) individuals from 475 families. Genotyping was completed using an Affymetrix Genome-Wide Human SNP Array 5.0 with imputation of 1,000 Genomes project SNPs from this array using IMPUTE2 and the Phase I v2, cosmopolitan (integrated) reference panel, build 37.

DR's EXTRA (Dose-Responses to Exercise Training): The Dose-Responses to Exercise Training (DR's EXTRA) Study is a 4-year RCT on the effects of regular physical exercise and healthy diet on endothelial function, atherosclerosis and cognition in a randomly selected population sample (n=3000) of Eastern Finnish men and women, identified from the national population register, aged

55-74 years. Of the eligible sample, 1410 individuals were randomized into one of the 6 groups: aerobic exercise, resistance exercise, diet, combined aerobic exercise and diet, combined resistance exercise and diet, or reference group following baseline assessments. During the four year intervention the drop-out rate was 15%.

EGCUT (Estonian Genome Center - University of Tartu (Estonian Biobank)): The Estonian Biobank is the population-based biobank of the Estonian Genome Center at the University of Tartu (www.biobank.ee; EGCUT). The entire project is conducted according to the Estonian Gene Research Act and all of the participants have signed the broad informed consent. The cohort size is up to 51535 individuals from 18 years of age and up, which closely reflects the age, sex and geographical distribution of the Estonian population. All of the subjects are recruited randomly by general practitioners and physicians in hospitals. A Computer Assisted Personal interview is filled within 1-2 hours at a doctor's office, which includes personal, genealogical, educational, occupational history and lifestyle data. Anthropometric measurements, blood pressure and resting heart rate are measured and venous blood taken during the visit. Medical history and current health status is recorded according to ICD-10 codes.

EPIC (European Prospective Investigation into Cancer and Nutrition)-Norfolk: The European Prospective Investigation of Cancer (EPIC)-Norfolk study is a population-based cohort study established to study the links diet, lifestyle factors and cancer and other health outcomes. Participants are men and women who were aged between 40 and 79 when they joined the study and who lived in Norwich, UK and the surrounding towns and rural areas. They have been contributing information about their diet, lifestyle and health through questionnaires and health checks over two decades. The Norwich Local Research Ethics Committee granted ethical approval for the study. All participants gave written informed consent.

The EPIC-InterAct Case-Cohort Study: EPIC- InterAct is a type 2 diabetes case-cohort study nested within the European Prospective Investigation into Cancer and Nutrition (EPIC) study. EPIC was initiated in the late 1980s and involves collaboration between 23 research institutions across Europe in 10 countries. The majority of EPIC cohorts were recruited from the general population, with some exceptions. French cohorts included women who were members of a health insurance scheme for school and university employees; Turin and Ragusa (Italy) and the Spanish centres included some blood donors. Participants from Utrecht (Netherlands) and Florence (Italy) were recruited via a breast cancer screening program. The majority of participants recruited by the EPIC Oxford (UK) centre consisted of vegetarian and "health conscious" volunteers from England, Wales, Scotland, and Northern Ireland. EPIC-InterAct sampled a random sub-cohort and all individuals who subsequently developed incident T2DM over follow up from the full cohort of participants in EPIC who provided blood samples at baseline in Denmark, France, Germany, Italy, the Netherlands, Spain, Sweden and the UK. Smoking status was determined at baseline and lipid measurements at baseline were undertaken in a centralized laboratory on all participants in the case-cohort study.

GeneSTAR (Genetic Studies of Atherosclerosis Risk): GeneSTAR is a family-based prospective study of more than 4000 participants begun in 1983 to determine phenotypic and genetic causes of premature cardiovascular disease. Families were identified from 1983-2006 from probands with a premature coronary disease event prior to 60 years of age who were identified at the time of hospitalization in any of 10 hospitals in the Baltimore, Maryland area. Their apparently healthy 30-59 year old siblings without known coronary disease were recruited and screened between 1983 and 2006. From 2003-2006, adult offspring over 21 years of age of all participating siblings and probands, as well as the coparents of the offspring were recruited and screened. Genotyping was

performed in 3,232 participants on the Illumina 1Mv1_c platform. Demographic information including education level was self-reported by participants. Medical history including medication use was assessed by the study nurse or physician.

GLACIER (Gene x Lifestyle Interactions and Complex Traits Involved in Elevated Disease Risk): The Gene-Lifestyle interactions And Complex traits Involved in Elevated disease Risk (GLACIER) Study is nested within the Västerbotten Intervention Programme, which is part of the Northern Sweden Health and Disease Study, a population-based prospective cohort study from northern Sweden. Participants were genotyped with Illumina CardioMetaboChip array. This array contains ~200,000 variants, the majority being common variants. Systolic and diastolic blood pressures were measured once following a period of five minutes rest with the participant in the supine position using a mercury-gauge sphygmomanometer. Analysis of serum lipids (HDL-C, triglycerides and total cholesterol) were undertaken at the Department of Clinical Chemistry at Umeå University Hospital using routine methods. LDL-C was determined using the Friedewald formula. All participants completed a detailed, optically readable, health and lifestyle questionnaire including questions about smoking status and alcohol intake (FFQ). Cohort description - PMID: 25396097

GRAPHIC (Genetic Regulation of Arterial Pressure of Humans in the Community): The GRAPHIC Study comprises 2024 individuals from 520 nuclear families recruited from the general population in Leicestershire, UK between 2003-2005 for the purpose of investigating the genetic determinants of blood pressure and related cardiovascular traits. A detailed medical history was obtained from study subjects by standardized questionnaires and clinical examination was performed by research nurses following standard procedures. Measurements obtained included height, weight, waist-hip ratio, clinic and ambulatory blood pressure and a 12-lead ECG.

HCHS/SOL (Hispanic Community Health Study/ Study of Latinos): The HCHS/SOL is a community-based cohort study of 16,415 self-identified Hispanic/Latino persons aged 18–74 years and selected from households in predefined census-block groups across four US field centers (in Chicago, Miami, the Bronx, and San Diego). The census-block groups were chosen to provide diversity among cohort participants with regard to socioeconomic status and national origin or background. The HCHS/SOL cohort includes participants who self-identified as having a Hispanic/Latino background; the largest groups are Central American (n = 1,730), Cuban (n = 2,348), Dominican (n = 1,460), Mexican (n = 6,471), Puerto Rican (n = 2,728), and South American (n = 1,068). The HCHS/SOL baseline clinical examination occurred between 2008 and 2011 and included comprehensive biological, behavioral, and sociodemographic assessments. Consenting HCHS/SOL subjects were genotyped at Illumina on the HCHS/SOL custom 15041502 B3 array. The custom array comprised the Illumina Omni 2.5M array (HumanOmni2.5-8v.1-1) ancestry-informative markers, known GWAS hits and drug absorption, distribution, metabolism, and excretion (ADME) markers, and additional custom content including ~150,000 SNPs selected from the CLM (Colombian in Medellin, Colombia), MXL (Mexican Ancestry in Los Angeles, California), and PUR (Puerto Rican in Puerto Rico) samples in the 1000Genomes phase 1 data to capture a greater amount of Amerindian genetic variation. QA/QC procedures yielded a total of 12,803 unique study participants for imputation and downstream association analyses.

HRS (Health & Retirement Study): The Health and Retirement Study (HRS) is a longitudinal survey of a representative sample of Americans over the age of 50. The current sample is over 26,000 persons in 17,000 households. Respondents are interviewed every two years about income and wealth, health and use of health services, work and retirement, and family connections. DNA

was extracted from saliva collected during a face-to-face interview in the respondents' homes. These data represent respondents who provided DNA samples and signed consent forms in 2006, 2008, and 2010. Respondents were removed if they had missing genotype or phenotype data.

1. Juster, F. T., Suzman, R. (1995). An Overview of the Health and Retirement Study, Journal of Human Resources 30:Suppl: S7-S56.

2. Sonnega A, Faul JD, Ofstedal MB, Langa KM, Phillips JWR, Weir DR. Cohort Profile: the Health and Retirement Study (HRS). Int. J. Epidemiol. 2014; 43 (2): 576-585. PMID: 24671021.

3. Crimmins, E.M., Guyer H., Langa K.M., Ofstedal M.B., Wallace R.B., and Weir D.R. (2008).

    - Documentation of Physical Measures, Anthropometrics and Blood Pressure in the Health and Retirement Study. HRS Documentation Report DR-011. http://hrsonline.isr.umich.edu/sitedocs/userg/dr-011.pdf

HyperGEN-AXIOM (Hypertension Genetic Epidemiology Network): HyperGEN is a family-based study that investigates the genetic causes of hypertension and related conditions in EA and AA subjects. HyperGEN recruited hypertensive sibships, along with their normotensive adult offspring, and an age-matched random sample. HyperGEN has collected data on 2,471 Caucasian-American subjects and 2,300 African-American subjects, from five field centers in Alabama, Massachusetts, Minnesota, North Carolina, and Utah. HyperGEN participates as a discovery study using GWAS available in a large subset of the samples. The remaining AA subjects without GWAS data were genotyped on the Affymetrix Axiom chip as part of a HyperGEN admixture mapping ancillary study. After excluding subjects already included in the original HyperGEN (or with family members included), this subset of approximately 450 AA subjects are included in the HyperGEN-AXIOM study which participates in replications.

1. Shetty PB, Tang H, Tayo BO, Morrison AC, Hanis CL, Rao DC, Young JH, Fox ER, Boerwinkle E, Cooper RS, Risch NJ, Zhu X; Candidate Gene Association Resource (CARe) Consortium. Variants in CXADR and F2RL1 are associated with blood pressure and obesity in African-Americans in regions identified through admixture mapping. J Hypertens. 2012 Oct;30(10):1970-6. PMID:22914544

INGI-CARL (INGI-Carlantino) and INGI-FVG (INGI-Friuli Venezia Giulia): INGI-FVG and INGI-CARL studies include samples coming from isolated populations and belong to the ITALIAN NETWORK OF GENETIC ISOLATES (INGI). Carlantino is a small village in the Province of Foggia in southern Italy. Genetic analyses of chromosome Y haplotypes as well as mitochondrial DNA show that Carlantino is a genetically homogeneous population and not only a geographically isolated village (DOI: 10.1038/ejhg.2012.229; DOI: 10.1038/s41431-019-0551-x). Participants were randomly selected in the range of 15 – 90 years of age. Genotyping and phenotypic data are available for 630 individuals. People with age < 18 were excluded from analyses. Subjects gave their written informed consent for participating in these studies. The project was approved by the local administration of Carlantino, the Health Service of Foggia Province, Italy, and the ethical committee of the IRCCS Burlo-Garofolo of Trieste.

The Friuli Venezia Giulia population represents a collection of six villages covering a total area of 7858 km2 in a hilly part of Friuli-Venezia Giulia (FVG) county located in north-eastern Italy. Recent studies characterized this population as a genetic isolate with high level of genomic homozygosity

and elevated linkage disequilibrium (DOI: 10.1038/ejhg.2012.229; DOI: 10.1038/s41431-019-0551-x). The cohort accounts for 1590 genotyped samples. Participants were randomly selected people 3-92 years of age. Genotyping and phenotypic data for 1590 samples are available. People with age < 18 were excluded from analyses. A written informed consent for participation was obtained from all subjects. The project was approved by the Ethical committee of the IRCCS Burlo-Garofolo."

IRAS (Insulin Resistance Atherosclerosis Study): The Insulin Resistance Atherosclerosis Study (IRAS) was an epidemiologic cohort study designed to examine the relationship between insulin resistance and carotid atherosclerosis across a range of glucose tolerance. Individuals of self-reported Mexican-American ethnicity were recruited in San Antonio, TX and San Luis Valley, CO. Recruitment was balanced across age and glucose tolerance status. Inclusion of IRAS data is limited to 194 normoglycemic individuals with genotype data from the Illumina OmniExpress and Omni 1S arrays and imputation to the 1000 Genomes Integrated Reference Panel (phase I).

IRASFS (Insulin Resistance Atherosclerosis Family Study): The IRASFS was a family study designed to examine the genetic and epidemiologic basis of glucose homeostasis traits and abdominal adiposity. Briefly, self-reported Mexican American pedigrees were recruited in San Antonio, TX and San Luis Valley, CO. Probands with large families were recruited from the initial non-family-based IRAS, which was modestly enriched for impaired glucose tolerance and T2D. Inclusion of IRASFS data is limited to 1040 normoglycemic individuals in 88 pedigrees with genotype data from the Illumina OmniExpress and Omni 1S arrays and imputation to the 1000 Genomes Integrated Reference Panel (phase I).

JUPITER (Justification for the Use of Statins in Primary Prevention: An Intervention Trial Evaluating Rosuvastatin): Genetic analysis was performed in a sub-population from JUPITER (Justification for the Use of statins in Prevention: an Intervention Trial Evaluating Rosuvastatin), an international, randomized, placebo-controlled trial of rosuvastatin (20mg/day) in the primary prevention of cardiovascular disease conducted among apparently healthy men and women with LDL-C < 130 mg/dL and hsCRP ≥ 2 mg/L (1, 2). Individuals with diabetes or triglyceride concentration >500mg/dL were excluded. The present analysis includes only individuals who provided consent for genetic analysis, had successfully collected genotype information, and who had either verified European or verified South African black ancestry.

1. Ridker PM, Danielson E, Fonseca FA, Genest J, Gotto AM Jr, Kastelein JJ, Koenig W, Libby P, Lorenzatti AJ, MacFadyen JG, Nordestgaard BG, Shepherd J, Willerson JT, Glynn RJ; JUPITER Study Group. Rosuvastatin to prevent vascular events in men and women with elevated C-reactive protein. N Engl J Med 2008 Nov 20; 359(21):2195-207

2. Chasman DI, Giulianini F, MacFadyen J, Barratt BJ, Nyberg F, Ridker PM. Genetic determinants of statin-induced low-density lipoprotein cholesterol reduction: the Justification for the Use of Statins in Prevention: an Intervention Trial Evaluating Rosuvastatin (JUPITER) trial. Circ Cardiovasc Genet. 2012 Apr 1;5(2):257-64. doi: 10.1161/CIRCGENETICS.111.961144. Epub 2012 Feb 13. Erratum in: Circ Cardiovasc Genet. 2012 Jun;5(3):e27. PubMed PMID: 22331829.

KORA (Cooperative Health Research in the Augsburg Region): The KORA study is a series of independent population-based epidemiological surveys of participants living in the region of Augsburg, Southern Germany. All survey participants are residents of German nationality identified

through the registration office and were examined in 1994/95 (KORA S3) and 1999/2001 (KORA S4). In the KORA S3 and S4 studies 4,856 and 4,261 subjects have been examined implying response rates of 75% and 67%, respectively. 3,006 subjects participated in a 10-year follow-up examination of S3 in 2004/05 (KORA F3), and 3080 of S4 in 2006/2008 (KORA F4). The age range of the participants was 25 to 74 years at recruitment. Informed consent has been given by all participants. The study has been approved by the local ethics committee. Individuals for genotyping in KORA F3 and KORA F4 were randomly selected and these genotypes are taken for the analysis of the phenotypes in KORA S3 and KORA S4.

LBC1936 (Lothian Birth Cohort 1936): LBC1936 consists of 1091 (548 male) relatively healthy individuals who underwent cognitive and medical testing at a mean age of 69.6 years (SD = 0.8). They were born in 1936, most took part in the Scottish Mental Survey of 1947, and almost all lived independently in the Lothian region of Scotland.[1,2]

1. Deary IJ, Gow AJ, Pattie A, Starr JM. Cohort profile: the Lothian Birth Cohorts of 1921 and 1936. Int J Epidemiol 2012;41:1576-1584.

2. Taylor AM, Pattie A, Deary IJ. Cohort Profile Update: The Lothian Birth Cohorts of 1921 and 1936. Int J Epidemiol 2018;47:1042-1042r

Lifelines (Netherlands Biobank): Lifelines (https://lifelines.nl/) is a multi-disciplinary prospective population-based cohort study using a unique three-generation design to examine the health and health-related behaviors of 165,000 persons living in the North East region of The Netherlands. It employs a broad range of investigative procedures in assessing the biomedical, socio-demographic, behavioral, physical and psychological factors which contribute to the health and disease of the general population, with a special focus on multimorbidity. In addition, the Lifelines project comprises a number of cross-sectional sub-studies, which investigate specific age-related conditions. These include investigations into metabolic and hormonal diseases, including obesity, cardiovascular and renal diseases, pulmonary diseases and allergy, cognitive function and depression, and musculoskeletal conditions. All survey participants are between 18 and 90 years old at the time of enrollment. Recruitment has been going on since the end of 2006, and over 130,000 participants had been included by April 2013. At the baseline examination, the participants in the study were asked to fill in a questionnaire (on paper or online) before the first visit. During the first and second visit, the first or second part of the questionnaire, respectively, are checked for completeness, a number of investigations are conducted, and blood and urine samples are taken. Lifelines is a facility that is open for all researchers. Information on application and data access procedure is summarized on www.lifelines.nl. (Scholtens S, Smidt N, Swertz MA, Bakker SJ, Dotinga A, Vonk JM, et al. Cohort Profile: LifeLines, a three-generation cohort study and biobank. Int J Epidemiol. 2014 Dec 14.)

- LifeLines group authors (genetics): Behrooz Z Alizadeh,[1] H Marike Boezen,[1] Lude Franke,[2] Pim van der Harst,[3] Gerjan Navis,[4] Marianne Rots,[5] Harold Snieder,[1] Morris Swertz,[2] Bruce HR Wolffenbuttel,[6] Cisca Wijmenga.[2]

- [1] Department of Epidemiology, University of Groningen, University Medical Center Groningen, The Netherlands; [2] Department of Genetics, University of Groningen, University Medical Center Groningen, The Netherlands; [3] Department of Cardiology, University of Groningen, University Medical Center Groningen, The Netherlands; [4] Department of Internal Medicine, Division of Nephrology, University of Groningen, University Medical Center Groningen, The Netherlands; [5] Department of Pathology and Medical Biology, University of Groningen, University Medical Center

Groningen, The Netherlands; [6] Department of Endocrinology, University of Groningen, University Medical Center Groningen, The Netherlands.

LLFS (The Long Life Family Study): LLFS is a family-based cohort study, including four clinical centers: Boston University Medical Center in Boston, MA, USA, Columbia University College of Physicians and Surgeons in New York City, NY, USA, the University of Pittsburgh in Pittsburgh PA, USA, and University of Southern Denmark, Denmark. The study characteristics, recruitment, eligibility and enrollment have been previously described (Pedersen et al., 2006, PMID: 17150149; Sebastiani et al., 2009, PMID: 19910380; Newman et al., 2011, PMID: 21258136). In brief, the LLFS was designed to determine genetic, behavioral, and environmental factors related to families of exceptionally healthy, elderly individuals. Phase 1 was conducted between 2006 and 2009 recruiting 4,953 individuals from 539 families. The probands were at least 79 years old in the USA centers, and 90 years old or above in Denmark. The families were selected to participate in the study based on The Family Longevity Selection Score (FLoSS) (Sebastiani et al., 2009, PMID: 19910380), a score generated according to birth-year cohort survival probabilities of the proband and siblings; probands and their families with FLoSS score of 7 or higher, at least one living sibling, and at least one living offspring (minimum family size of 3), who were able to give informed consent and willing to participate were recruited. The individuals were genotyped using ~2.3 million SNPs from the Illumina Omni chip, and then imputed on phased 1000 Genomes with Cosmopolitan data as a reference using MACH and MINIMAC. After excluding participants with 80 years and older, ~3,200 individuals have been included in the analyses for replication.

Loyola GxE (Kingston Gene-by-environment; subset of International Collaborative Study of Hypertension in Blacks (ICSHIB)): The Kingston GxE cohort was obtained from a survey conducted in Kingston, Jamaica as part of a larger project to examine gene by environment interactions in the determination of blood pressure among adults 25-74 years [PMID: 9103091]. The principal criterion for eligibility was a body mass index in either the top or bottom third of BMI for the Jamaican population. Participants were identified principally from the records of the Heart Foundation of Jamaica, a non-governmental organization based in Kingston, which provides low-cost screening services (height and weight, blood pressure, glucose, cholesterol) to the general public. Other participants were identified from among participants in family studies of blood pressure at the Tropical Metabolism Research Unit (TMRU) and from among staff members at the University of the West Indies, Mona.

Loyola SPT (Spanish Town; subset of International Collaborative Study of Hypertension in Blacks (ICSHIB)): Participants were recruited from Spanish Town, a stable, residential urban area neighboring the capital city of Kingston, Jamaica as part of the ICSHIB [PMID: 9103091]. A stratified random sampling scheme was used to recruit adult males and females aged 25–74 years from the general population. Spanish Town was chosen because its demographic make-up was broadly representative of Jamaica as a whole.

NESDA (Netherlands Study of Depression and Anxiety): NESDA is a multi-center study designed to examine the long-term course and consequences of depressive and anxiety disorders (http://www.nesda.nl)[1]. NESDA included both individuals with depressive and/or anxiety disorders and controls without psychiatric conditions. Inclusion criteria were age 18-65 years and self-reported western European ancestry while exclusion criteria were not being fluent in Dutch and having a primary diagnosis of another psychiatric condition (psychotic disorder, obsessive compulsive disorder, bipolar disorder, or severe substance use disorder).

1. Penninx, B.W. et al. The Netherlands Study of Depression and Anxiety (NESDA): rationale, objectives and methods. Int J Methods Psychiatr Res 17, 121-40 (2008).

PREVEND (The Prevention of REnal and Vascular ENd stage Disease study): The PREVEND study is an ongoing prospective study investigating the natural course of increased levels of urinary albumin excretion and its relation to renal and cardiovascular disease. Inhabitants 28 to 75 years of age (n=85,421) in the city of Groningen, The Netherlands, were asked to complete a short questionnaire, 47% responded, and individuals were then selected with a urinary albumin concentration of at least 10 mg/L (n = 7,768) and a randomly selected control group with a urinary albumin concentration less than 10 mg/L (n = 3,395). Details of the protocol have been described elsewhere (Hillege HL et al. Circulation 2002;106:1777-82).

RHS (Ragama Health Study): The Ragama Health Study (RHS) is a population-based study of South Asian men and women aged 35-64yrs living in the Ragama Medical Officer of Health (MOH) area, near Colombo, Sri Lanka.* Consenting adults attended a clinic after a 12-h fast with available health records, and were interviewed by trained personnel to obtain information on medical, sociodemographic, and lifestyle variables. A 10-mL sample of venous blood was obtained from each subject. The concurrent study was performed in two tea plantation estates in the Lindula MOH area, near Nuwara Eliya (180 km from Colombo), to investigate the gene-environment interaction in a community with differing lifestyles (e.g., physical activity and diet). The RHS is a collaborative effort between the Faculty of Medicine, University of Kelaniya and the National Center for Global Health and Medicine, Japan.

1. Dassanayake, A.S. et al. Prevalence and risk factors for non-alcoholic fatty liver disease among adults in an urban Sri Lankan population. J Gastroenterol Hepatol 24, 1284-8 (2009).

SHEEP (Stockholm Heart Epidemiology Project): The SHEEP is a population based case-control study of risk factors for first episode of acute myocardial infarction. The study base comprised all Swedish citizens resident in the Stockholm county 1992-1994 who were 45-70 years of age and were free of previous clinically diagnosed myocardial infarction.

Cases were identified using three different sources: 1) coronary units and internal medicine wards for acute care in all Stockholm hospitals; 2) the National Patient Register; and 3) death certificates. For the present study, only cases who survived at least 28 days were considered (n=1213).

First time incident myocardial infarction cases (n=1213) were identified during a 2-year period (1992-1993) for men and during a 3-year period (1992-1994) for women. Controls (n=1561) were randomly recruited from the study population continuously over time within 2 days of the case occurrence and matched to cases on age (5-years interval), sex and hospital catchment area using computerized registers of the population of Stockholm. Five control candidates were sampled simultaneously to be able to replace potential non-respondent controls. Occasionally, because of late response of the initial control, both the first and alternative controls were considered resulting in the inclusion of more controls than cases. Postal questionnaires covering a wide range of exposure areas including occupational exposures, life style factors, social factors and health related factors were distributed to the participants. Clinical investigations were performed at least three months after myocardial infarction of cases and their matched controls. The investigations included blood samplings under fasting conditions with collection of whole blood for DNA extraction, serum and plasma. A biobank was established containing DNA, serum and plasma.

Exposure information based on both the questionnaire and biological data from the health examination was available for 78% of the male and 67% of the female non-fatal cases; the corresponding figures for their controls were 68% and 64%.

SHIP (Study of Health in Pomerania): The Study of Health In Pomerania (SHIP) is a prospective longitudinal population-based cohort study in Mecklenburg-West Pomerania assessing the prevalence and incidence of common diseases and their risk factors (PMID: 20167617). SHIP encompasses the two independent cohorts SHIP and SHIP-TREND. Participants aged 20 to 79 with German citizenship and principal residency in the study area were recruited from a random sample of residents living in the three local cities, 12 towns as well as 17 randomly selected smaller towns. Individuals were randomly selected stratified by age and sex in proportion to population size of the city, town or small towns, respectively. A total of 4,308 participants were recruited between 1997 and 2001 in the SHIP cohort. Between 2008 and 2012 a total of 4,420 participants were recruited in the SHIP-TREND cohort. Individuals were invited to the SHIP study centre for a computer-assisted personal interviews and extensive physical examinations. The study protocol was approved by the medical ethics committee of the University of Greifswald. Oral and written informed consents was obtained from each of the study participants

Genome-wide SNP-typing was performed using the Affymetrix Genome-Wide Human SNP Array 6.0 or the Illumina Human Omni 2.5 array (SHIP-TREND samples). Array processing was carried out in accordance with the manufacturer's standard recommendations. Genotypes were determined using GenomeStudio Genotyping Module v1.0 (GenCall) for SHIP-TREND and the Birdseed2 clustering algorithm for SHIP. Imputation of genotypes in SHIP and SHIP-TREND was performed with the software IMPUTE v2.2.2 based on 1000 Genomes release March 2012.

SWHS/SMHS (Shanghai Women's Health Study/ Shanghai Men's Health Study): The Shanghai Women's Health Study (SWHS) is an ongoing population-based cohort study of approximately 75,000 women who were aged 40-70 years at study enrollment and resided in in urban Shanghai, China; 56,832 (75.8%) provided a blood samples. Recruitment for the SWHS was initiated in 1997 and completed in 2000. The self-administered questionnaire includes information on demographic characteristics, disease and surgery histories, personal habits (such as cigarette smoking, alcohol consumption, tea drinking, and ginseng use), menstrual history, residential history, occupational history, and family history of cancer.

The Shanghai Men's Health Study (SMHS) is an ongoing population-based cohort study of 61,480 Chinese men who were aged between 40 and 74 years, were free of cancer at enrollment, and lived in urban Shanghai, China; 45,766 (74.4%) provided a blood samples. Recruitment for the SMHS was initiated in 2002 and completed in 2006. The self-administered questionnaire includes information on demographic characteristics, disease and surgery histories, personal habits (such as cigarette smoking, alcohol consumption, tea drinking, and ginseng use), residential history, occupational history, and family history of cancer.

Genotyping and imputation: Genomic DNA was extracted from buffy coats by using a Qiagen DNA purification kit (Valencia, CA) or Puregene DNA purification kit (Minneapolis, MN) according to the manufacturers' instructions and then used for genotyping assays. The GWAS genotyping was performed using the Affymetrix Genome-Wide Human SNP Array 6.0 (Affy6.0) platform or Illumina 660, following manufacturers' protocols. After sample quality control, we exclude SNPs with 1) MAF <0.01; 2) call rate <95%; 2) bad genotyping cluster; and 3) concordance rate <95%

among duplicated QC samples. Genotypes were imputed using the program MACH (http://www.sph.umich.edu/csg/abecasis/MACH/download/), which determines the probable distribution of missing genotypes conditional on a set of known haplotypes, while simultaneously estimating the fine-scale recombination map. Phased autosome SNP data from HapMap Phase II Asians (release 22) were used as the reference. To test for associations between the imputed SNP data with BMI, linear regression (additive model) was used, in which SNPs were represented by the expected allele count, an approach that takes into account the degree of uncertainty of genotype imputation (http://www.sph.umich.edu/csg/abecasis/MACH/download/).

The lipid profiles were measured at Vanderbilt Lipid Laboratory. Total cholesterol, high-density lipoprotein (HDL) cholesterol, and triglycerides (TG) were measured using an ACE Clinical Chemistry System (Alfa Wassermann, Inc, West Caldwell, NJ). Low-density lipoprotein (LDL) cholesterol levels were calculated by using the Friedwald equation. The levels of LDL cholesterol were directly measured using an ACE Clinical Chemistry System for subjects with TG levels ≥ 400 mg/dL. Fasting status was defined as an interval between the last meal and blood draw of 8 hours or longer.

TWINGENE (TwinGene of the Swedish Twin Registry): The aim of the TwinGene project has been to systematically transform the oldest cohorts of the Swedish Twin Registry (STR) into a molecular-genetic resource. Beginning in 2004, about 200 twins were contacted each month until the data collection was completed in 2008. A total of 21 500 twins were contacted where of 12 600 participated. Invitations to the study contained information of the study and its purpose. Along with the invitations consent forms and health questionnaire were sent to the subjects. When the signed consent forms where returned, the subjects were sent blood sampling equipment and asked to contact a local health facility for blood sampling. The study population was recruited among twins participating in the Screening Across the Lifespan Twin Study (SALT) which was a telephone interview study conducted in 1998-2002. Other inclusion criteria were that both twins in the pair had to be alive and living in Sweden. Subjects were excluded from the study if they preciously declined participation in future studies or if they had been enrolled in other STR DNA sampling projects. The subjects were asked to make an appointment for a health check-up at their local health-care facility on the morning Monday to Thursday and not the day before a national holiday, this to ensure that the sample would reach the KI biobank the following morning by overnight mail. The subjects were instructed to fast from 20.00 the previous night. By venipuncture a total of 50 ml of blood was drawn from each subject. Tubes with serum and blood for biobanking as well as for clinical chemistry tests were sent to KI by overnight mail. One 7ml EDTA tube of whole blood is stored in -80°C while a second 7ml EDTA tube of blood is used for DNA extraction using Puregene extraction kit (Gentra systems, Minneapolis, USA). After excluding subjects in which the DNA concentration in the stock-solution was below 20ng/µl as well as subset of 302 female monozygous twin pairs participating in a previous genome wide effort DNA from 9896 individual subjects was sent to SNP&SEQ Technology Platform Uppsala, Sweden for genome wide genotyping with Illumina OmniExpress bead chip (all available dizygous twins + one twin from each available MZ twin pair).

YFS (The Cardiovascular Risk in Young Finns Study): The YFS is a population-based follow up-study started in 1980. The main aim of the YFS is to determine the contribution made by childhood lifestyle, biological and psychological measures to the risk of cardiovascular diseases in adulthood. In 1980, over 3,500 children and adolescents all around Finland participated in the baseline study. The follow-up studies have been conducted mainly with 3-year intervals. The latest 30-year follow-up study was conducted in 2010-11 (ages 33-49 years) with 2,063 participants. The study was approved by the local ethics committees (University Hospitals of Helsinki, Turku, Tampere, Kuopio

and Oulu) and was conducted following the guidelines of the Declaration of Helsinki. All participants gave their written informed consent.

STAGE 1 STUDY ACKNOWLEDGMENTS

Infrastructure for the CHARGE Consortium is supported in part by the National Heart, Lung, and Blood Institute grant R01HL105756. Infrastructure for the Gene-Lifestyle Working Group is supported by the National Heart, Lung, and Blood Institute grant R01HL118305.

AGES (Age Gene/Environment Susceptibility Reykjavik Study): This study has been funded by NIH contract N01-AG012100, HSSN271201200022C, the NIA Intramural Research Program, an Intramural Research Program Award (ZIAEY000401) from the National Eye Institute, an award from the National Institute on Deafness and Other Communication Disorders (NIDCD) Division of Scientific Programs (IAA Y2-DC_1004-02), Hjartavernd (the Icelandic Heart Association), and the Althingi (the Icelandic Parliament). The study is approved by the Icelandic National Bioethics Committee, VSN: 00-063. The researchers are indebted to the participants for their willingness to participate in the study.

ARIC (Atherosclerosis Risk in Communities) Study: The ARIC study has been funded in whole or in part with Federal funds from the National Heart, Lung, and Blood Institute, National Institutes of Health, Department of Health and Human Services, under Contract nos. (75N92022D00001, 75N92022D00002, 75N92022D00003, 75N92022D00004, 75N92022D00005). The authors thank the staff and participants of the ARIC study for their important contributions. Funding was also supported by R01HL087641 and R01HL086694; National Human Genome Research Institute contract U01HG004402; and National Institutes of Health contract HHSN268200625226C. Infrastructure was partly supported by Grant Number UL1RR025005, a component of the National Institutes of Health and NIH Roadmap for Medical Research.

Baependi Heart Study (Brazil): The Baependi Heart Study was supported by Fundação de Amparo a Pesquisa do Estado de São Paulo (FAPESP) (Grant 2013/17368-0), Coordenação de Aperfeiçoamento de Pessoal de Nível Superior (CAPES) and Hospital Samaritano Society (Grant 25000.180.664/2011-35), through Ministry of Health to Support Program Institutional Development of the Unified Health System (SUS-PROADI).

CARDIA (Coronary Artery Risk Development in Young Adults): The Coronary Artery Risk Development in Young Adults Study (CARDIA) is conducted and supported by the National Heart, Lung, and Blood Institute (NHLBI) in collaboration with the University of Alabama at Birmingham (HHSN268201800005I & HHSN268201800007I), Northwestern University (HHSN268201800003I), University of Minnesota (HHSN268201800006I), and Kaiser Foundation Research Institute (HHSN268201800004I). Genotyping was funded as part of the NHLBI Candidate-gene Association Resource (N01-HC-65226) and the NHGRI Gene Environment Association Studies (GENEVA) (U01-HG004729, U01-HG04424, and U01-HG004446). This manuscript has been reviewed by CARDIA for scientific content.

CHS (Cardiovascular Health Study): This Cardiovascular Health Study (CHS) research was supported by NHLBI contracts HHSN268201200036C, HHSN268200800007C, HHSN268200960009C, HHSN268201800001C N01HC55222, N01HC85079, N01HC85080,

N01HC85081, N01HC85082, N01HC85083, N01HC85086, 75N92021D00006; and NHLBI grants U01HL080295, U01HL130114, R01HL087652, R01HL105756, R01HL103612, R01HL085251, and R01HL120393 with additional contribution from the National Institute of Neurological Disorders and Stroke (NINDS). Additional support was provided through R01AG023629 from the National Institute on Aging (NIA). A full list of principal CHS investigators and institutions can be found at CHS-NHLBI.org. The provision of genotyping data was supported in part by the National Center for Advancing Translational Sciences, CTSI grant UL1TR001881, and the National Institute of Diabetes and Digestive and Kidney Disease Diabetes Research Center (DRC) grant DK063491 to the Southern California Diabetes Endocrinology Research Center. The content is solely the responsibility of the authors and does not necessarily represent the official views of the National Institutes of Health.

CROATIA-Korcula: We would like to acknowledge the staff of several institutions in Croatia that supported the field work, including but not limited to The University of Split and Zagreb Medical Schools and the Croatian Institute for Public Health. We would like to acknowledge the invaluable contributions of the recruitment team in Korcula, the administrative teams in Croatia and Edinburgh and the participants. The SNP genotyping for the CROATIA-Korcula cohort was performed in Helmholtz Zentrum München, Neuherberg, Germany. CROATIA-Korcula (CRKorcula) was funded by the Medical Research Council UK, The Croatian Ministry of Science, Education and Sports (grant 216-1080315-0302), the European Union framework program 6 EUROSPAN project (contract no. LSHG-CT-2006-018947), the Croatian National Centre of Research Excellence in Personalized Healthcare (grant number KK.01.1.1.01.0010), and the Centre of Competence in Molecular Diagnostics (KK.01.2.2.03.0006). JEH is now Director of Genomics Research, Center for Population Genomics. Boston VA, and JM is currently an employee of Genomics PLC, but both completed this work at the Medical Research Council Human Genetics Unit at the University of Edinburgh.

CROATIA-Vis: We would like to acknowledge the staff of several institutions in Croatia that supported the field work, including but not limited to The University of Split and Zagreb Medical Schools, the Institute for Anthropological Research in Zagreb and Croatian Institute for Public Health. The SNP genotyping for the CROATIA-Vis cohort was performed in the core genotyping laboratory of the Wellcome Trust Clinical Research Facility at the Western General Hospital, Edinburgh, Scotland. CROATIA-Vis (CR-Vis) was funded by the Medical Research Council UK, The Croatian Ministry of Science, Education and Sports (grant 216-1080315-0302), and the European Union framework program 6 EUROSPAN project (contract no. LSHG-CT-2006-018947).

ERF (Erasmus Rucphen Family study): The ERF study as a part of EUROSPAN (European Special Populations Research Network) was supported by European Commission FP6 STRP grant number 018947 (LSHG-CT-2006-01947) and also received funding from the European Community's Seventh Framework Programme (FP7/2007-2013)/grant agreement HEALTH-F4-2007-201413 by the European Commission under the programme "Quality of Life and Management of the Living Resources" of 5th Framework Programme (no. QLG2-CT-2002-01254). High-throughput analysis of the ERF data was supported by joint grant from Netherlands Organisation for Scientific Research and the Russian Foundation for Basic Research (NWO-RFBR 047.017.043). We are grateful to all study participants and their relatives, general practitioners and neurologists for their contributions and to P. Veraart for her help in genealogy, J. Vergeer for the supervision of the laboratory work, P. Snijders for his help in data collection and E.M. van Leeuwen for genetic imputation.

FamHS (Family Heart Study): The FamHS is funded by R01HL118305 and R01HL117078 NHLBI grants, and 5R01DK07568102 and 5R01DK089256 NIDDK grant.

FHS (Framingham Heart Study): This research was conducted in part using data and resources from the Framingham Heart Study of the National Heart Lung and Blood Institute of the National Institutes of Health and Boston University School of Medicine. The analyses reflect intellectual input and resource development from the Framingham Heart Study investigators participating in the SNP Health Association Resource (SHARe) project. This work was partially supported by the National Heart, Lung and Blood Institute's Framingham Heart Study (Contract Nos. N01-HC-25195 and HHSN268201500001I) and its contract with Affymetrix, Inc for genotyping services (Contract No. N02-HL-6-4278). A portion of this research utilized the Linux Cluster for Genetic Analysis (LinGA-II) funded by the Robert Dawson Evans Endowment of the Department of Medicine at Boston University School of Medicine and Boston Medical Center. This research was partially supported by grant R01-DK089256 from the National Institute of Diabetes and Digestive and Kidney Diseases (MPIs: Ingrid B. Borecki, L. Adrienne Cupples, Kari North).

GENOA (Genetic Epidemiology Network of Arteriopathy): Support for GENOA was provided by the National Heart, Lung and Blood Institute (HL119443, HL118305, HL054464, HL054457, HL054481, HL071917 and HL087660) of the National Institutes of Health. Genotyping was performed at the Mayo Clinic (Stephen T. Turner, MD, Mariza de Andrade PhD, Julie Cunningham, PhD). We thank Eric Boerwinkle, PhD and Megan L. Grove from the Human Genetics Center and Institute of Molecular Medicine and Division of Epidemiology, University of Texas Health Science Center, Houston, Texas, USA for their help with genotyping. We would also like to thank the families that participated in the GENOA study.

GenSalt (Genetic Epidemiology Network of Salt Sensitivity): The Genetic Epidemiology Network of Salt Sensitivity is supported by research grants (U01HL072507, R01HL087263, and R01HL090682) from the National Heart, Lung, and Blood Institute, National Institutes of Health, Bethesda, MD.

GS:SFHS: Generation Scotland received core support from the Chief Scientist Office of the Scottish Government Health Directorates [CZD/16/6] and the Scottish Funding Council [HR03006] and is currently supported by the Wellcome Trust [216767/Z/19/Z]. Genotyping of the GS:SFHS samples was carried out by the Genetics Core Laboratory at the Edinburgh Clinical Research Facility, University of Edinburgh, Scotland and was funded by the Medical Research Council UK and the Wellcome Trust (Wellcome Trust Strategic Award "STratifying Resilience and Depression Longitudinally" (STRADL) Reference 104036/Z/14/Z). Ethical approval for the GS:SFHS study was obtained from the Tayside Committee on Medical Research Ethics (on behalf of the National Health Service).

We are grateful to all the families who took part, the general practitioners and the Scottish School of Primary Care for their help in recruiting them, and the whole Generation Scotland team, which includes interviewers, computer and laboratory technicians, clerical workers, research scientists, volunteers, managers, receptionists, healthcare assistants and nurses.

CH was supported by an MRC Human Genetics Unit programme grant 'Quantitative traits in health and disease' (U. MC_UU_00007/10).

GOLDN (Genetics of Diet and Lipid Lowering Network): Support for the genome-wide association studies in GOLDN was provided by the National Heart, Lung, and Blood Institute

grant U01HL072524-04 and R01HL091357.

HANDLS (Healthy Aging in Neighborhoods of Diversity across the Life Span): The Healthy Aging in Neighborhoods of Diversity across the Life Span (HANDLS) study was supported by the Intramural Research Program of the NIH, National Institute on Aging and the National Center on Minority Health and Health Disparities (project # Z01-AG000513 and human subjects protocol number 09-AG-N248). Data analyses for the HANDLS study utilized the high-performance computational resources of the Biowulf Linux cluster at the National Institutes of Health, Bethesda, MD. (http://biowulf.nih.gov; http://hpc.nih.gov)).

Health ABC (Health, Aging, and Body Composition): Health ABC was funded by the National Institutes of Aging. This research was supported by NIA contracts N01AG62101, N01AG62103, and N01AG62106. The GWAS was funded by NIA grant 1R01AG032098-01A1 to Wake Forest University Health Sciences and genotyping services were provided by the Center for Inherited Disease Research (CIDR). CIDR is fully funded through a federal contract from the National Institutes of Health to The Johns Hopkins University, contract number HHSN268200782096C. This research was supported in part by the Intramural Research Program of the NIH, National Institute on Aging.

HERITAGE (Health, Risk Factors, Exercise Training and Genetics): The HERITAGE Family Study was supported by National Heart, Lung, and Blood Institute grant HL-45670.

HyperGEN (Hypertension Genetic Epidemiology Network): The hypertension network is funded by cooperative agreements (U10) with NHLBI: HL54471, HL54472, HL54473, HL54495, HL54496, HL54497, HL54509, HL54515, and 2 R01 HL55673-12. The study involves: University of Utah: (Network Coordinating Center, Field Center, and Molecular Genetics Lab); Univ. of Alabama at Birmingham: (Field Center and Echo Coordinating and Analysis Center); Medical College of Wisconsin: (Echo Genotyping Lab); Boston University: (Field Center); University of Minnesota: (Field Center and Biochemistry Lab); University of North Carolina: (Field Center); Washington University: (Data Coordinating Center); Weil Cornell Medical College: (Echo Reading Center); National Heart, Lung, & Blood Institute. For a complete list of HyperGEN Investigators: http://www.biostat.wustl.edu/hypergen/Acknowledge.html

JHS (Jackson Heart Study): The Jackson Heart Study is supported by contracts HSN268201300046C, HHSN268201300047C, HHSN268201300048C, HHSN268201300049C, HHSN268201300050C from the National Heart, Lung, and Blood Institute on Minority Health and Health Disparities. The authors acknowledge the Jackson Heart Study team institutions (University of Mississippi Medical Center, Jackson State University and Tougaloo College) and participants for their long-term commitment that continues to improve our understanding of the genetic epidemiology of cardiovascular and other chronic diseases among African Americans.

MESA (Multi-Ethic Study of Atherosclerosis) and the **MESA SHARe** projects are conducted and supported by the National Heart, Lung, and Blood Institute (NHLBI) in collaboration with MESA investigators. Support for MESA is provided by contracts 75N92020D00001, HHSN268201500003I, N01-HC-95159, 75N92020D00005, N01-HC-95160, 75N92020D00002, N01-HC-95161, 75N92020D00003, N01-HC-95162, 75N92020D00006, N01-HC-95163, 75N92020D00004, N01-HC-95164, 75N92020D00007, N01-HC-95165, N01-HC-95166, N01-HC-95167, N01-HC-95168, N01-HC-95169, UL1-TR-000040, UL1-TR-001079, UL1-TR-001420. Funding for SHARe genotyping was provided by NHLBI Contract N02-HL-64278. Genotyping was performed at Affymetrix (Santa Clara, California, USA) and the Broad Institute of Harvard and MIT (Boston, Massachusetts, USA) using the Affymetrix Genome-Wide Human SNP Array 6.0. Also supported in

part by the National Center for Advancing Translational Sciences, CTSI grant UL1TR001881, and the National Institute of Diabetes and Digestive and Kidney Disease Diabetes Research Center (DRC) grant DK063491 to the Southern California Diabetes Endocrinology Research Center. Infrastructure for the CHARGE Consortium is supported in part by the National Heart, Lung, and Blood Institute (NHLBI) grant R01HL105756.

NEO (The Netherlands Epidemiology of Obesity study): The authors of the NEO study thank all individuals who participated in the Netherlands Epidemiology in Obesity study, all participating general practitioners for inviting eligible participants and all research nurses for collection of the data. We thank the NEO study group, Petra Noordijk, Pat van Beelen and Ingeborg de Jonge for the coordination, lab and data management of the NEO study. The genotyping in the NEO study was supported by the Centre National de Génotypage (Paris, France), headed by Jean-Francois Deleuze. The NEO study is supported by the participating Departments, the Division and the Board of Directors of the Leiden University Medical Center, and by the Leiden University, Research Profile Area Vascular and Regenerative Medicine. Dennis Mook-Kanamori is supported by Dutch Science Organization (ZonMW-VENI Grant 916.14.023).

Pelotas Birth Cohort Study (The 1982 Pelotas Birth Cohort Study, Brazil): The 1982 Pelotas Birth Cohort Study is conducted by the Postgraduate Program in Epidemiology at Universidade Federal de Pelotas with the collaboration of the Brazilian Public Health Association (ABRASCO). From 2004 to 2013, the Wellcome Trust supported the study. The International Development Research Center, World Health Organization, Overseas Development Administration, European Union, National Support Program for Centers of Excellence (PRONEX), the Brazilian National Research Council (CNPq), and the Brazilian Ministry of Health supported previous phases of the study.

Genotyping of 1982 Pelotas Birth Cohort Study participants was supported by the Department of Science and Technology (DECIT, Ministry of Health) and National Fund for Scientific and Technological Development (FNDCT, Ministry of Science and Technology), Funding of Studies and Projects (FINEP, Ministry of Science and Technology, Brazil), Coordination of Improvement of Higher Education Personnel (CAPES, Ministry of Education, Brazil).

RS (Rotterdam Study): The Rotterdam Study is supported by the Erasmus MC University Medical Center and Erasmus University Rotterdam; The Netherlands Organisation for Scientific Research (NWO); The Netherlands Organisation for Health Research and Development (ZonMw); the Research Institute for Diseases in the Elderly (RIDE); The Netherlands Genomics Initiative (NGI); the Ministry of Education, Culture and Science; the Ministry of Health, Welfare and Sports; the European Commission (DG XII); and the Municipality of Rotterdam. The contribution of inhabitants, general practitioners and pharmacists of the Ommoord district to the Rotterdam Study is gratefully acknowledged. The contributions to the Management Team specifically and the Rotterdam Study at large of the following persons are pivotal to the daily operations of the study and highly appreciated: Jacobus Lubbe, Gabriëlle Bakker, Eric Neeleman, Jeannette Vergeer, Anneke Korving, Pauline van Wijngaarden, Jolande Verkroost – van Heemst, Silvan Licher, and Isabel van der Velpen.

The generation and management of GWAS genotype data for the Rotterdam Study was executed by the Human Genotyping Facility of the Genetic Laboratory of the Department of Internal Medicine, Erasmus MC, Rotterdam, The Netherlands. The GWAS datasets are supported by the Netherlands Organisation of Scientific Research NWO Investments (nr. 175.010.2005.011, 911-03-012), the

Genetic Laboratory of the Department of Internal Medicine, Erasmus MC, the Research Institute for Diseases in the Elderly (014-93-015; RIDE2), the Netherlands Genomics Initiative (NGI)/Netherlands Organisation for Scientific Research (NWO) Netherlands Consortium for Healthy Aging (NCHA), project nr. 050-060-810. We thank Pascal Arp, Mila Jhamai, Marijn Verkerk, Lizbeth Herrera, Marjolein Peters and Carolina Medina-Gomez for their help in creating the GWAS database, and Karol Estrada, Yurii Aulchenko and Carolina Medina-Gomez for the creation and analysis of imputed data.

SCES (Singapore Chinese Eye Study), SiMES (Singapore Malay Eye Study), SINDI (Singapore Indian Eye Study): The Singapore Malay Eye Study (SiMES), the Singapore Indian Eye Study (SINDI), and the Singapore Chinese Eye Study (SCES) are supported by the National Medical Research Council (NMRC), Singapore (grants 0796/2003, 1176/2008, 1149/2008, STaR/0003/2008, 1249/2010, CG/SERI/2010, CIRG/1371/2013, and CIRG/1417/2015), and Biomedical Research Council (BMRC), Singapore (08/1/35/19/550 and 09/1/35/19/616). Ching-Yu Cheng is supported by an award from NMRC (CSA/033/2012). The Singapore Tissue Network and the Genome Institute of Singapore, Agency for Science, Technology and Research, Singapore provided services for tissue archival and genotyping, respectively.

SCHS-CHD (Singapore Chinese Health Study - Coronary Heart Disease): The Singapore Chinese Health Study is supported by the National Institutes of Health, USA (RO1 CA144034 and UM1 CA182876), the nested case-control study of myocardial infarction by the Singapore National Medical Research Council (NMRC 1270/2010) and genotyping by the HUJ-CREATE Programme of the National Research Foundation, Singapore (Project Number 370062002).

SP2 (Singapore Prospective Study Program): The SP2 study is supported by individual research and clinical scientist award schemes from the National Medical Research Council (NMRC) and the Biomedical Research Council (BMRC) of Singapore, the Singapore Ministry of Health, National University of Singapore and National University Health System, Singapore. The Genome Institute of Singapore, Agency for Science, Technology and Research, Singapore provided services for genotyping.

WGHS (Women's Genome Health Study): The WGHS is supported by the National Heart, Lung, and Blood Institute (HL043851 and HL080467) and the National Cancer Institute (CA047988 and UM1CA182913), with collaborative scientific support and funding for genotyping provided by Amgen.

STAGE 2 STUDY ACKNOWLEDGMENTS

Infrastructure for the CHARGE Consortium is supported in part by the National Heart, Lung, and Blood Institute grant R01HL105756. Infrastructure for the Gene-Lifestyle Working Group is supported by the National Heart, Lung, and Blood Institute grant R01HL118305.

AA-DHS (African American Diabetes Heart Study): The investigators acknowledge the cooperation of our Diabetes Heart Study (DHS) and AA-DHS participants. This work was supported by NIH R01 DK071891, R01 HL092301, R01 AG058921, and the General Clinical Research Center of Wake Forest School of Medicine M01-RR-07122.

BES (Beijing Eye Study): The Beijing Eye Study has been funded by the National Natural Science Foundation of China (grant 81170890, 81570835) and the Beijing Municipal of Health Reform and Development Project (grant 2019-4).

BRIGHT (British Genetics of Hypertension): This work was supported by the Medical Research Council of Great Britain (grant number G9521010D) and the British Heart Foundation (grant number PG/02/128). The BRIGHT study is extremely grateful to all the patients who participated in the study and the BRIGHT nursing team. This work forms part of the research program of the National Institutes of Health Research (NIHR Cardiovascular Biomedical Research) Cardiovascular Biomedical Unit at Barts and The London, QMUL.

DESIR (Data from an Epidemiological Study on the Insulin Resistance): The DESIR study has been funded by INSERM contracts with Caisse nationale de l'assurance Maladie des Travailleurs Salariés (CNAMTS), Lilly, Novartis Pharma, and Sanofi-Aventis; INSERM (Réseaux en Santé Publique, Interactions entre les déterminants de la santé, Cohortes Santé TGIR 2008); the Association Diabète Risque Vasculaire; the Fédération Française de Cardiologie; La Fondation de France; Association de Langue Française pour l'Etude du Diabète et des Maladies Métaboliques (ALFEDIAM)/Société Francophone de Diabétologie (SFD); l'Office National Interprofessionnel des Vins (ONIVINS); Ardix Medical; Bayer Diagnostics; Becton Dickinson; Cardionics; Merck Santé; Novo Nordisk; Pierre Fabre; Roche; Topcon.

The DESIR Study Group. INSERM U1018: B. Balkau, P. Ducimetière, E. Eschwège; INSERM U367: F. Alhenc-Gelas; CHU D'Angers: Y Gallois, A. Girault; Centre de Recherche des Cordeliers, INSERM U1138, Bichat Hospital: F. Fumeron, M. Marre, R. Roussel; CHU de Rennes: F. Bonnet; CNRS UMR8090, Lille: A. Bonnefond, S. Cauchi, P. Froguel; Centres d'Examens de Santé: Alençon, Angers, Blois, Caen, Chateauroux, Chartres, Cholet, Le Mans, Orléans, Tours; Institut de Recherche Médecine Générale: J. Cogneau; General practitioners of the region; Institut inter-régional pour la Santé: C. Born, E. Caces, M. Cailleau, O Lantieri, J.G. Moreau.

DFTJ (Dongfeng-Tongji Cohort Study): This work was supported by grants from the Foundation of National Key Program of Research and Development of China (2016YFC0900800), the Programme of Introducing Talents of Discipline, the grants from the National Natural Science Foundation (grant NSFC-81473051, 81522040 and 81230069), and the Program for the New Century Excellent Talents in University (NCET-11-0169).

DHS (Diabetes Heart Study): The authors thank the investigators, staff, and participants of the DHS for their valuable contributions. This study was supported by the National Institutes of Health through R01 HL067348, R01 HL092301, and R01 AG058921.

DR's EXTRA (Dose-Responses to Exercise Training): The study was supported by grants from Ministry of Education and Culture of Finland (722 and 627; 2004-2010); Academy of Finland (102318, 104943, 123885, 211119); European Commission FP6 Integrated Project (EXGENESIS), LSHM-CT-2004-005272; City of Kuopio; Juho Vainio Foundation; Finnish Diabetes Association; Finnish Foundation for Cardiovascular Research; Kuopio University Hospital; Päivikki and Sakari Sohlberg Foundation; Social Insurance Institution of Finland 4/26/2010.

EGCUT (Estonian Genome Center - University of Tartu (Estonian Biobank)): This study was funded by EU H2020 grant 692145, 676550, 654248, Estonian Research Council Grant IUT20-60, IUT24-6, PUT1660, and European Union through the European Regional Development Fund (Project No. 2014-2020.4.01.15-0012), GENTRANSMED end Project No. 2014-2020.4.01.16-0125, NIH – GIANT and NIH-BMI Grant 2R01DK075787-06A1.

EPIC (European Prospective Investigation into Cancer and Nutrition)-Norfolk: The EPIC-Norfolk study (DOI 10.22025/2019.10.105.00004) has received funding from the Medical Research Council (MR/N003284/1, MC-PC_13048 and MC-UU_12015/1). We are grateful to all the participants who have been part of the project and to the many members of the study teams at the University of Cambridge who have enabled this research.

The EPIC-InterAct Case-Cohort Study: We thank all EPIC participants and staff for their contribution to the study. The InterAct study received funding from the European Union (Integrated Project LSHM-CT-2006-037197 in the Framework Programme 6 of the European Community). We thank staff from the Technical, Field Epidemiology and Data Functional Group Teams of the Medical Research Council Epidemiology Unit in Cambridge, UK, for carrying out sample preparation, DNA provision and quality control, genotyping and data handling work. We specifically thank Sarah Dawson for coordinating the sample provision for biomarker measurements, Abigail Britten for coordinating DNA sample provision and genotyping of candidate markers, Nicola Kerrison, Chris Gillson and Abigail Britten for data provision and genotyping quality control, Matt Sims for writing the technical laboratory specification for the intermediate pathway biomarker measurements and for overseeing the laboratory work.

GeneSTAR (Genetic Studies of Atherosclerosis Risk): GeneSTAR was supported by National Institutes of Health grants from the National Heart, Lung, and Blood Institute (HL49762, HL58625, HL071025, U01 HL72518, HL087698, HL092165, HL099747, and K23HL105897), National Institute of Nursing Research (NR0224103), National Institute of Neurological Disorders and Stroke (NS062059), and by grants from the National Center for Research Resources to the Johns Hopkins General Clinical Research Center (M01-RR000052) and the Johns Hopkins Institute for Clinical & Translational Research (UL1 RR 025005).

GLACIER (Gene x Lifestyle Interactions and Complex Traits Involved in Elevated Disease Risk): We thank the participants, health professionals and data managers involved in the Västerbotten Intervention Programme. We are also grateful to the staff of the Northern Sweden Biobank for preparing materials and to K Enqvist and T Johansson (Västerbottens County Council, Umeå, Sweden) for DNA preparation. The Västerbotten Intervention Programme is finance by Västerbotten County Council. The current study was supported by Novo Nordisk (PWF), the Swedish Research Council (PWF), the Swedish Heart Lung Foundation (PWF), the European Research Council (PWF), and the Skåne Health Authority (PWF). This study was also supported by the Swedish Research Council, Strategic Research Area Exodiab (Dnr 2009-1039), the Swedish

Foundation for Strategic Research (Dnr IRC15-0067) and the Swedish Research Council, Linnaeus grant (Dnr 349-2006-237).

GRAPHIC (Genetic Regulation of Arterial Pressure of Humans in the Community): The GRAPHIC Study was funded by the British Heart Foundation (BHF/RG/2000004). This work falls under the portfolio of research supported by the NIHR Leicester Cardiovascular Biomedical Research Unit. CPN and NJS are funded by the BHF and NJS is a NIHR Senior Investigator.

HCHS/SOL (Hispanic Community Health Study/ Study of Latinos): The baseline examination of HCHS/SOL was supported by contracts from the National Heart, Lung, and Blood Institute (NHLBI) to the University of North Carolina (N01-HC65233), University of Miami (N01-HC65234), Albert Einstein College of Medicine (N01-HC65235), Northwestern University (N01-HC65236), and San Diego State University (N01-HC65237). The National Institute on Minority Health and Health Disparities, National Institute on Deafness and Other Communication Disorders, National Institute of Dental and Craniofacial Research (NIDCR), National Institute of Diabetes and Digestive and Kidney Diseases (NIDDK), National Institute of Neurological Disorders and Stroke, and NIH Office of Dietary Supplements additionally contributed funding to HCHS/SOL. The Genetic Analysis Center at the University of Washington was supported by NHLBI and NIDCR contracts (HHSN268201300005C AM03 and MOD03). Additional analysis support was provided by 1R01DK101855-01 and 13GRNT16490017. Genotyping was also supported by National Center for Advancing Translational Sciences UL1TR000124 and NIDDK DK063491 to the Southern California Diabetes Endocrinology Research Center. This research was also supported in part by the Intramural Research Program of the NIDDK, contract no. HHSB268201200054C, and Illumina.

HRS (Health & Retirement Study): HRS is supported by the National Institute on Aging (NIA U01AG009740 and R03 AG046389). Genotyping was funded separately by NIA (RC2 AG036495, RC4 AG039029). Our genotyping was conducted by the NIH Center for Inherited Disease Research (CIDR) at Johns Hopkins University. Genotyping quality control and final preparation of the data were performed by the Genetics Coordinating Center at the University of Washington.

HyperGEN-AXIOM (Hypertension Genetic Epidemiology Network): The study was support by the National Institutes of Health, the National Heart, Lung, Blood Institute grant HL086718.

INGI-CARL (Italian Network Genetic Isolates): This study was partially supported by Regione FVG (L.26.2008) and Italian Ministry of Health (GR-2011-02349604).

INGI-FVG (Italian Network Genetic Isolates): This study was partially supported by Regione FVG (L.26.2008) and Italian Ministry of Health (GR-2011-02349604).

IRAS (Insulin Resistance Atherosclerosis Study): The IRAS is supported by the National Heart Lung Institute (HL047887, HL047889, HL047890, and HL47902). Genotyping and analysis for this study was supported by the GUARDIAN Consortium with grant support from the National Institute of Diabetes and Digestive and Kidney Diseases (NIDDK; DK085175 and DK118062) and in part by UL1TR000124 (CTSI) and DK063491 (DRC). The authors thank study investigators, staff, and participants for their valuable contributions.

IRASFS (Insulin Resistance Atherosclerosis Family Study): The IRASFS is supported by the National Heart Lung and Blood Institute (HL060944, HL061019, and HL060919). Genotyping and analysis for this study was supported by the GUARDIAN Consortium with grant support from the

National Institute of Diabetes and Digestive and Kidney Diseases (NIDDK; DK085175 and DK118062) and in part by UL1TR000124 (CTSI) and DK063491 (DRC). The authors thank study investigators, staff, and participants for their valuable contributions.

JUPITER (Justification for the Use of Statins in Primary Prevention: An Intervention Trial Evaluating Rosuvastatin): Support for genotype data collection and collaborative genetic analysis in JUPITER was provided by Astra-Zeneca.

KORA (Cooperative Health Research in the Augsburg Region): The KORA study was initiated and financed by the Helmholtz Zentrum München – German Research Center for Environmental Health, which is funded by the German Federal Ministry of Education and Research (BMBF) and by the State of Bavaria. Furthermore, KORA research was supported within the Munich Center of Health Sciences (MC-Health), Ludwig-Maximilians-Universität, as part of LMUinnovativ.

LBC1936 (Lothian Birth Cohort 1936): We thank the LBC1936 cohort participants and team members who contributed to these studies. Phenotype collection was supported by Age UK (The Disconnected Mind project). Genotyping was funded by the BBSRC (BB/F019394/1). The work was undertaken by The University of Edinburgh Centre for Cognitive Ageing and Cognitive Epidemiology, part of the cross council Lifelong Health and Wellbeing Initiative (MR/K026992/1). Funding from the BBSRC and Medical Research Council (MRC) is gratefully acknowledged. We gratefully acknowledge the contribution of Lothian Birth Cohort co-author Professor John M. Starr, who died prior to the publication of this manuscript.

LifeLines (Netherlands Biobank): The Lifelines Cohort Study, and generation and management of GWAS genotype data for the Lifelines Cohort Study is supported by the Netherlands Organization of Scientific Research NWO (grant 175.010.2007.006), the Economic Structure Enhancing Fund (FES) of the Dutch government, the Ministry of Economic Affairs, the Ministry of Education, Culture and Science, the Ministry for Health, Welfare and Sports, the Northern Netherlands Collaboration of Provinces (SNN), the Province of Groningen, University Medical Center Groningen, the University of Groningen, Dutch Kidney Foundation and Dutch Diabetes Research Foundation.

The authors wish to acknowledge the services of the Lifelines Cohort Study, the contributing research centers delivering data to Lifelines, and all the study participants.

LLFS (The Long Life Family Study): The study is supported by the National Institute on Aging (NIA) grant U01AG023746, U01AG023712, U01AG023749, U01AG023755, U01AG023744, and U19AG063893.

Loyola GxE (Kingston Gene-by-environment; subset of International Collaborative Study of Hypertension in Blacks (ICSHIB)): The Loyola GxE project was supported by NIH Grant R01HL53353.

Loyola SPT (Spanish Town; subset of International Collaborative Study of Hypertension in Blacks (ICSHIB)): The Loyola SPT project was supported by NIH Grant R01HL53353.

NESDA (Netherlands Study of Depression and Anxiety): Funding was obtained from the Netherlands Organization for Scientific Research (Geestkracht program grant 10-000-1002); the Center for Medical Systems Biology (CSMB, NWO Genomics), Biobanking and Biomolecular Resources Research Infrastructure (BBMRI-NL), VU University's Institutes for Health and Care Research (EMGO+) and Neuroscience Campus Amsterdam, University Medical Center Groningen,

Leiden University Medical Center, National Institutes of Health (NIH, R01D0042157-01A, MH081802, Grand Opportunity grants 1RC2 MH089951 and 1RC2 MH089995). Part of the genotyping and analyses were funded by the Genetic Association Information Network (GAIN) of the Foundation for the National Institutes of Health.Computing was supported by BiG Grid, the Dutch e-Science Grid, which is financially supported by NWO.

PREVEND (The Prevention of REnal and Vascular ENd stage Disease study): PREVEND genetics is supported by the Dutch Kidney Foundation (Grant E033), the EU project grant GENECURE (FP-6 LSHM CT 2006 037697), the National Institutes of Health (grant 2R01LM010098), The Netherlands organization for health research and development (NWO-Groot grant 175.010.2007.006, NWO VENI grant 916.761.70, ZonMw grant 90.700.441).

RHS (Ragama Health Study): The RHS was supported by the Grant of National Center for Global Health and Medicine (NCGM).

SHEEP (Stockholm Heart Epidemiology Project): This study was supported by grants from the Swedish Research Council for Health, Working Life and Welfare (http://www.forte.se/en/), the Stockholm County Council (http://www.sll.se/om-landstinget/Information-in-English1/), the Swedish Research Council (http://www.vr.se/inenglish.4.12fff4451215cbd83e4800015152.html), the Swedish Heart and Lung Foundation (https://www.hjart-lungfonden.se/HLF/Om-Hjart-lungfonden/About-HLF/), and the Cardiovascular Programme at Karolinska Institutet (http://ki.se/en/mmk/cardiovascular-research-networks).

SHIP (Study of Health in Pomerania): SHIP is part of the Community Medicine Research net of the University of Greifswald, Germany, which is funded by the Federal Ministry of Education and Research (grants no. 01ZZ9603, 01ZZ0103, and 01ZZ0403), the Ministry of Cultural Affairs as well as the Social Ministry of the Federal State of Mecklenburg-West Pomerania, and the network 'Greifswald Approach to Individualized Medicine (GANI_MED)' funded by the Federal Ministry of Education and Research (grant 03IS2061A). Genome-wide data were supported by the Federal Ministry of Education and Research (grant no. 03ZIK012) and a joint grant from Siemens Healthcare, Erlangen, Germany and the Federal State of Mecklenburg- West Pomerania. The University of Greifswald is a member of the 'Center of Knowledge Interchange' program of the Siemens AG.

SWHS/SMHS (Shanghai Women's Health Study/ Shanghai Men's Health Study): We thank all the individuals who took part in these studies and all the researchers who have enabled this work to be carried out. The Shanghai Women's Health Study and the Shanghai Men's Health Study are supported by research grants UM1CA182910 and UM1CA173640 from the U.S. National Cancer Institute, respectively.

TWINGENE (TwinGene of the Swedish Twin Registry): The Swedish Twin Registry is financially supported by Karolinska Institutet. TwinGene project received funding from the Swedish Research Council (M-2005-1112), GenomEUtwin (EU/QLRT-2001-01254; QLG2-CT-2002-01254), NIH DK U01-066134, The Swedish Foundation for Strategic Research (SSF) and the Heart and Lung foundation no. 20070481

YFS (The Cardiovascular Risk in Young Finns Study): The Young Finns Study has been financially supported by the Academy of Finland: grants 286284, 134309 (Eye), 126925, 121584, 124282, 129378 (Salve), 117787 (Gendi), and 41071 (Skidi); the Social Insurance Institution of

Finland; Kuopio, Tampere and Turku University Hospital Medical Funds (grant X51001); Juho Vainio Foundation; Paavo Nurmi Foundation; Finnish Foundation for Cardiovascular Research ; Finnish Cultural Foundation; Tampere Tuberculosis Foundation; Emil Aaltonen Foundation; Yrjö Jahnsson Foundation; Signe and Ane Gyllenberg Foundation; and Diabetes Research Foundation of Finnish Diabetes Association.

The expert technical assistance in the statistical analyses by Leo-Pekka Lyytikäinen and Irina Lisinen is gratefully acknowledged.

Figure S1: Flow Chart of Study Design

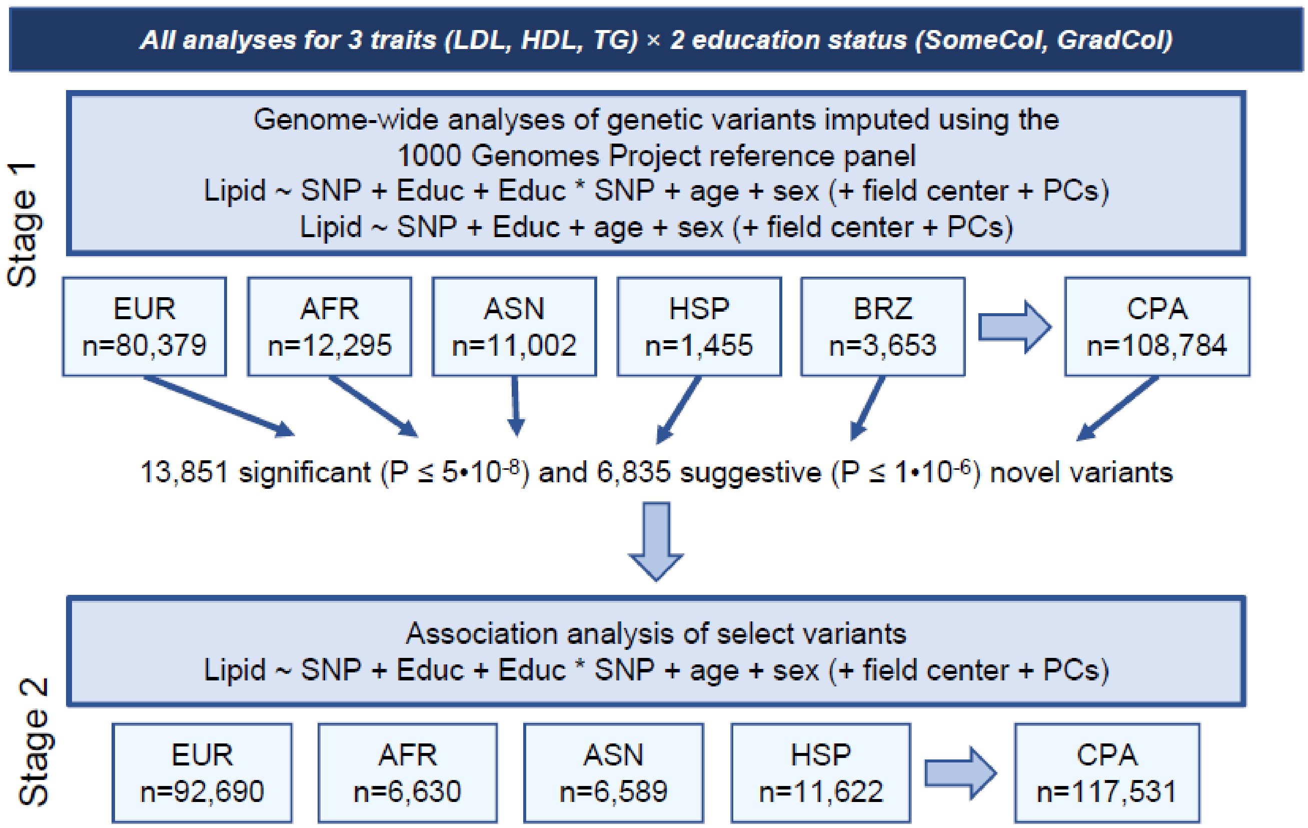

Figure S2A: QQ Plots of African-Ancestry Cohorts.

AFR LDL GradCol M1: DF >20

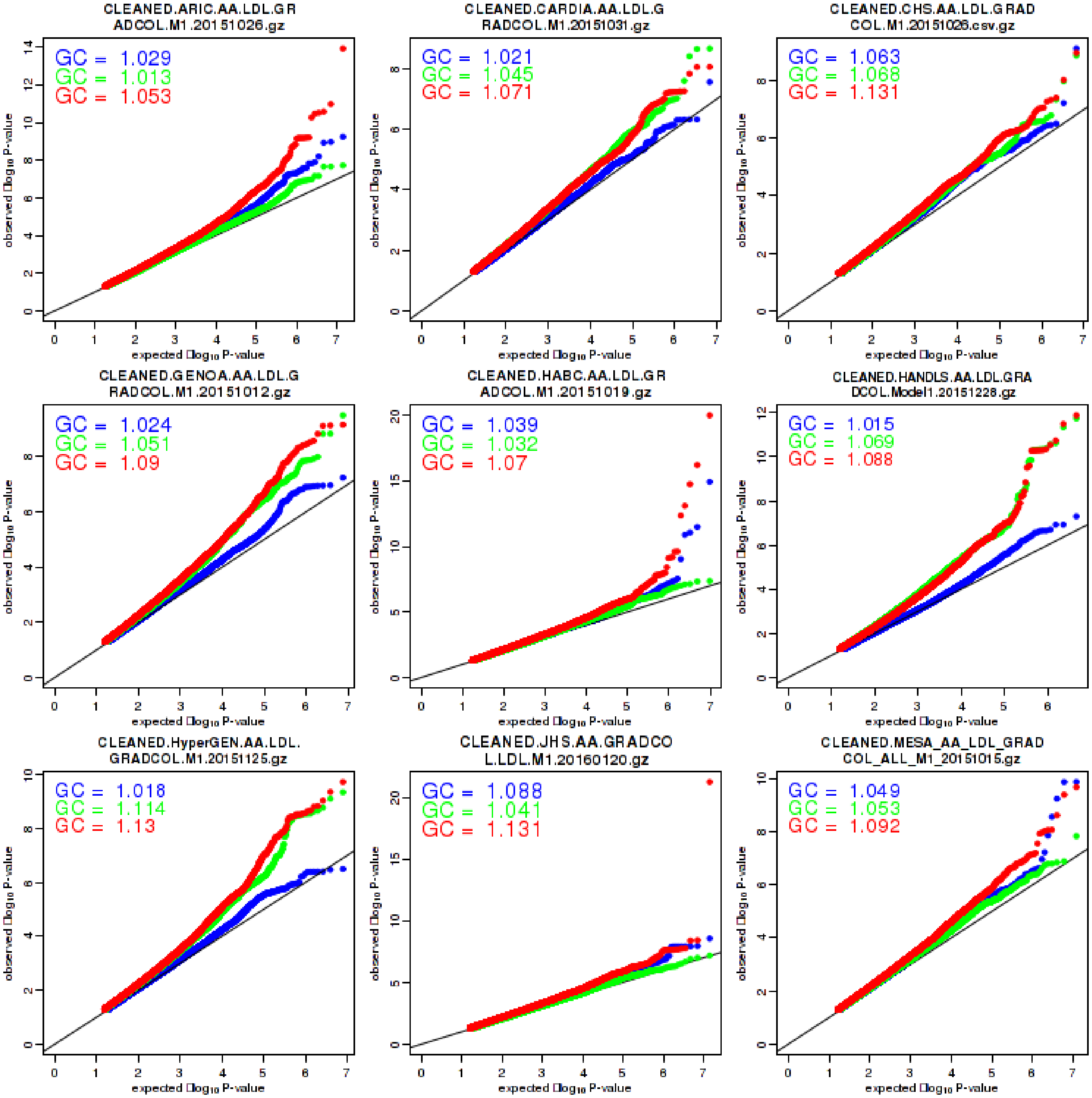

AFR LDL SomeCol M1: DF >20

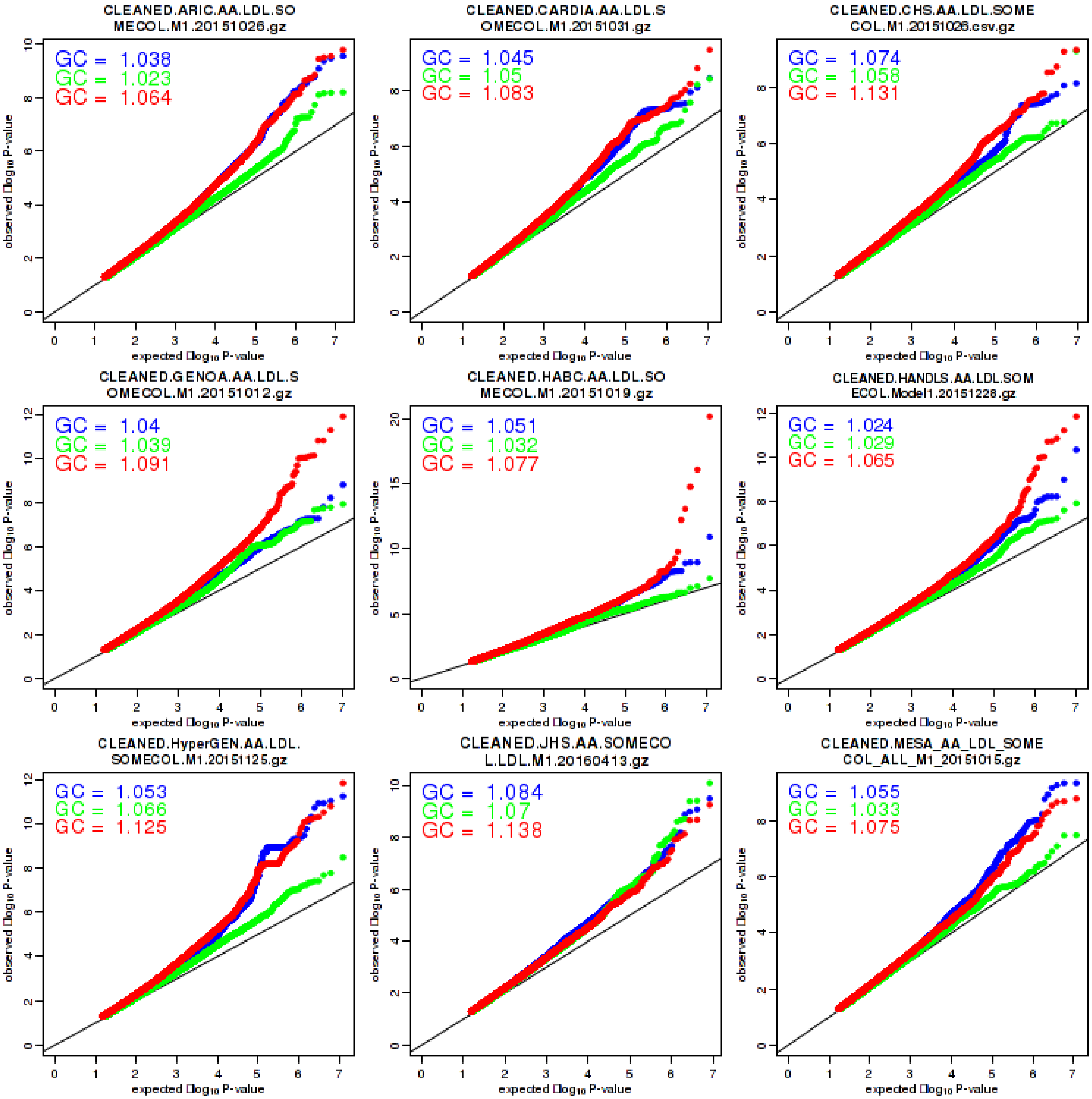

AFR HDL GradCol M1: DF >20

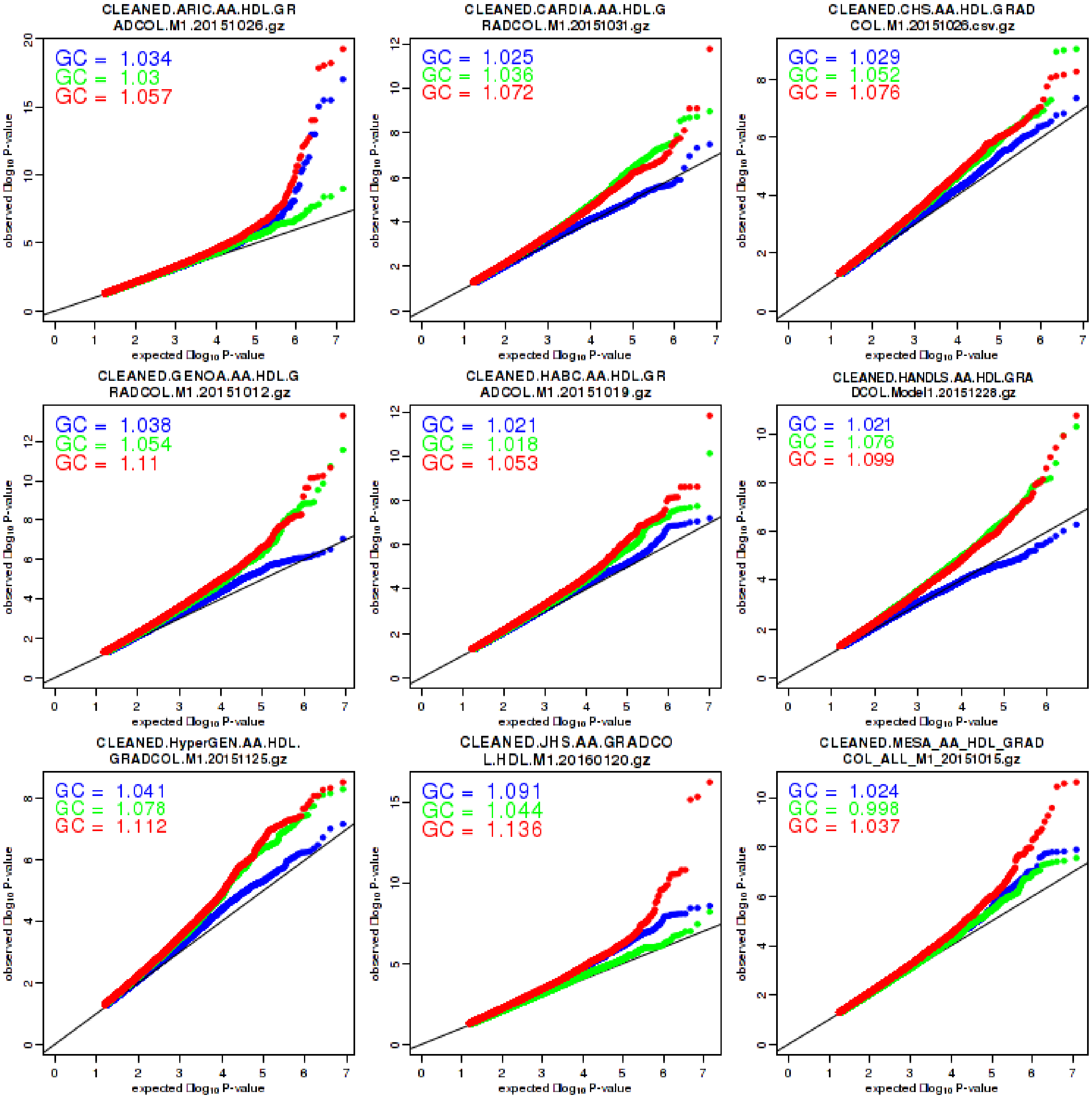

AFR HDL SomeCol M1: DF >20

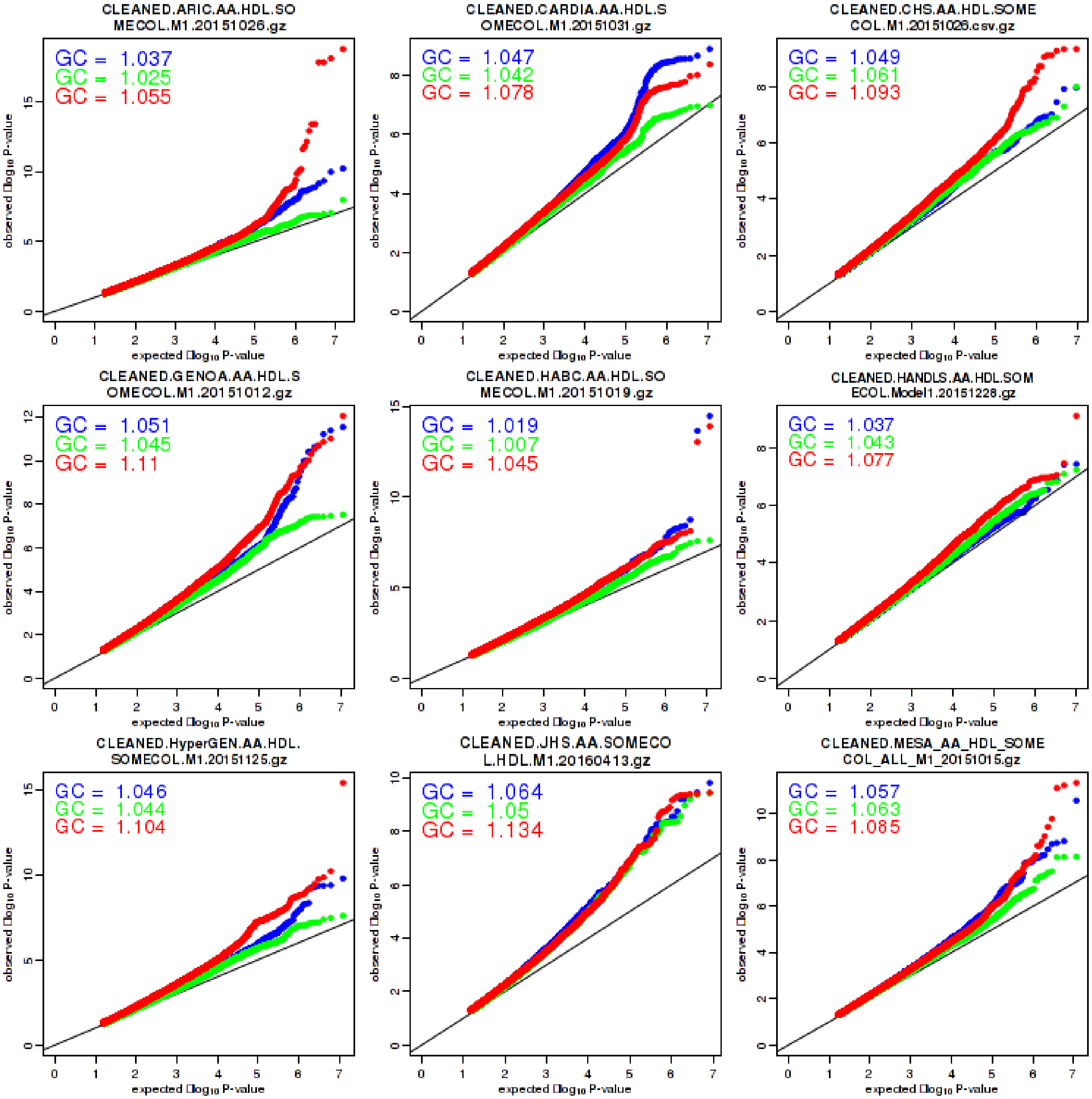

AFR TG GradCol M1: DF >20

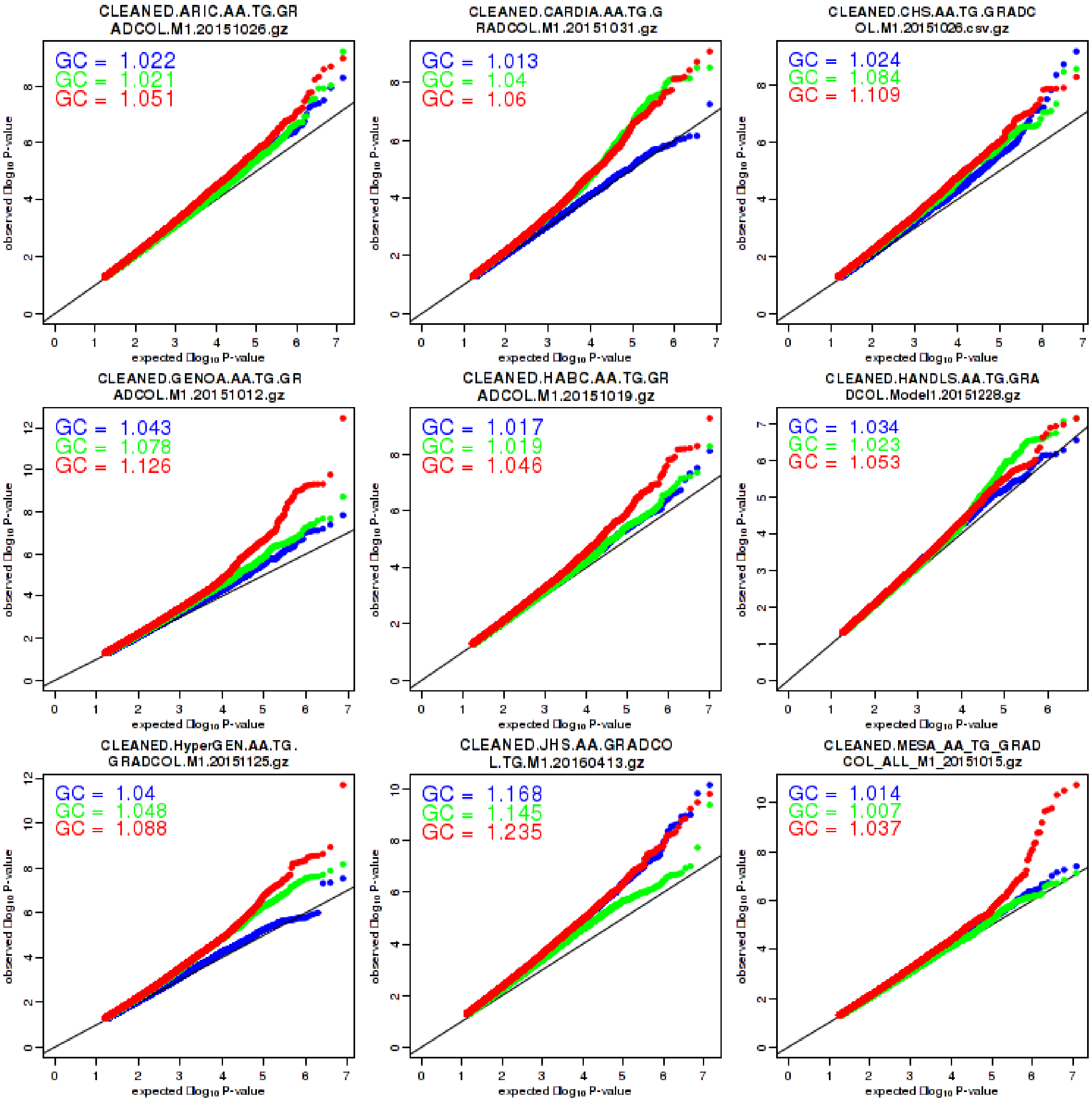

AFR TG SomeCol M1: DF >20

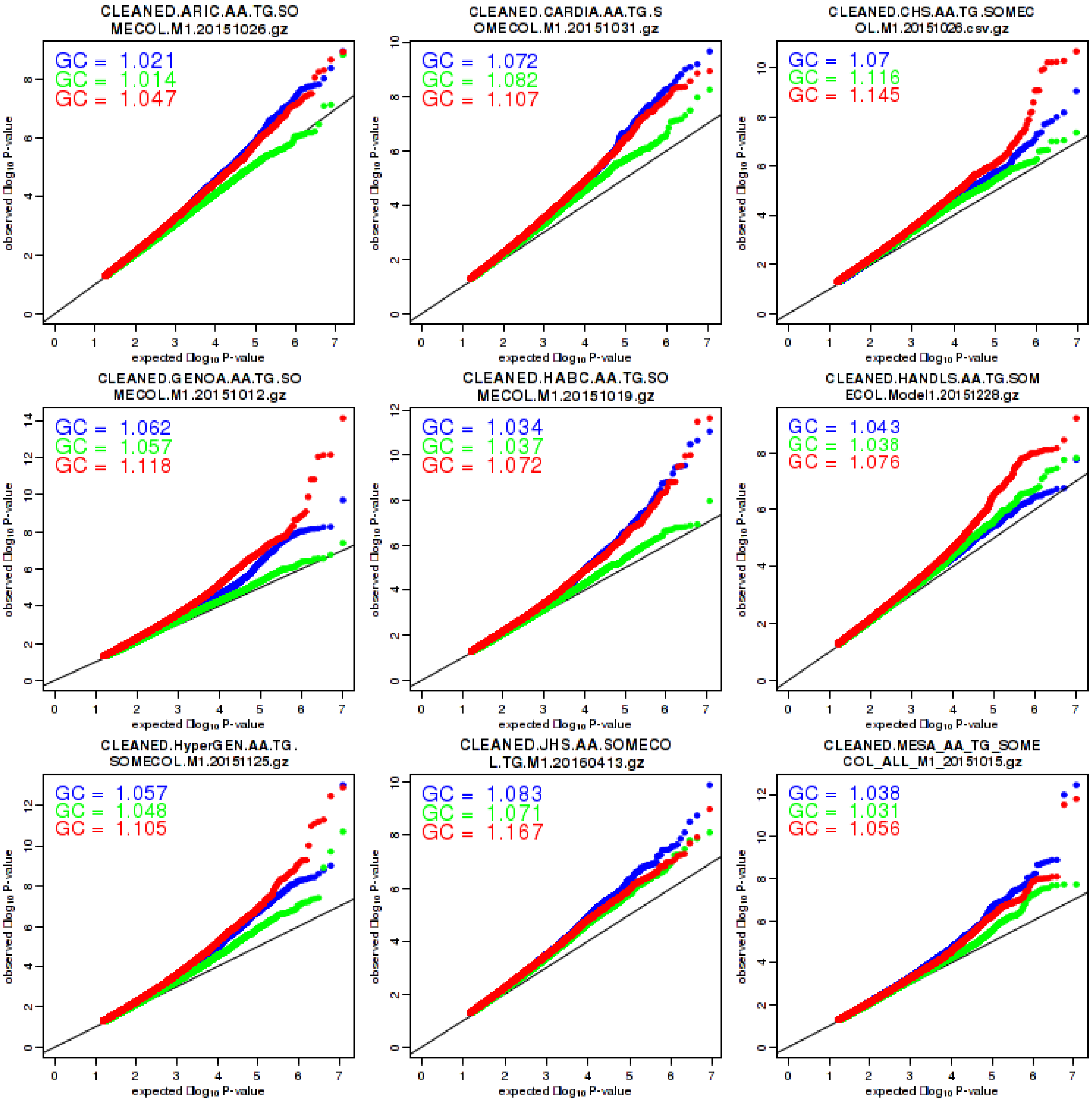

Figure S2B: QQ Plots of Asian-Ancestry Cohorts

<u>**EAS LDL GradCol M1: DF >20**</u>

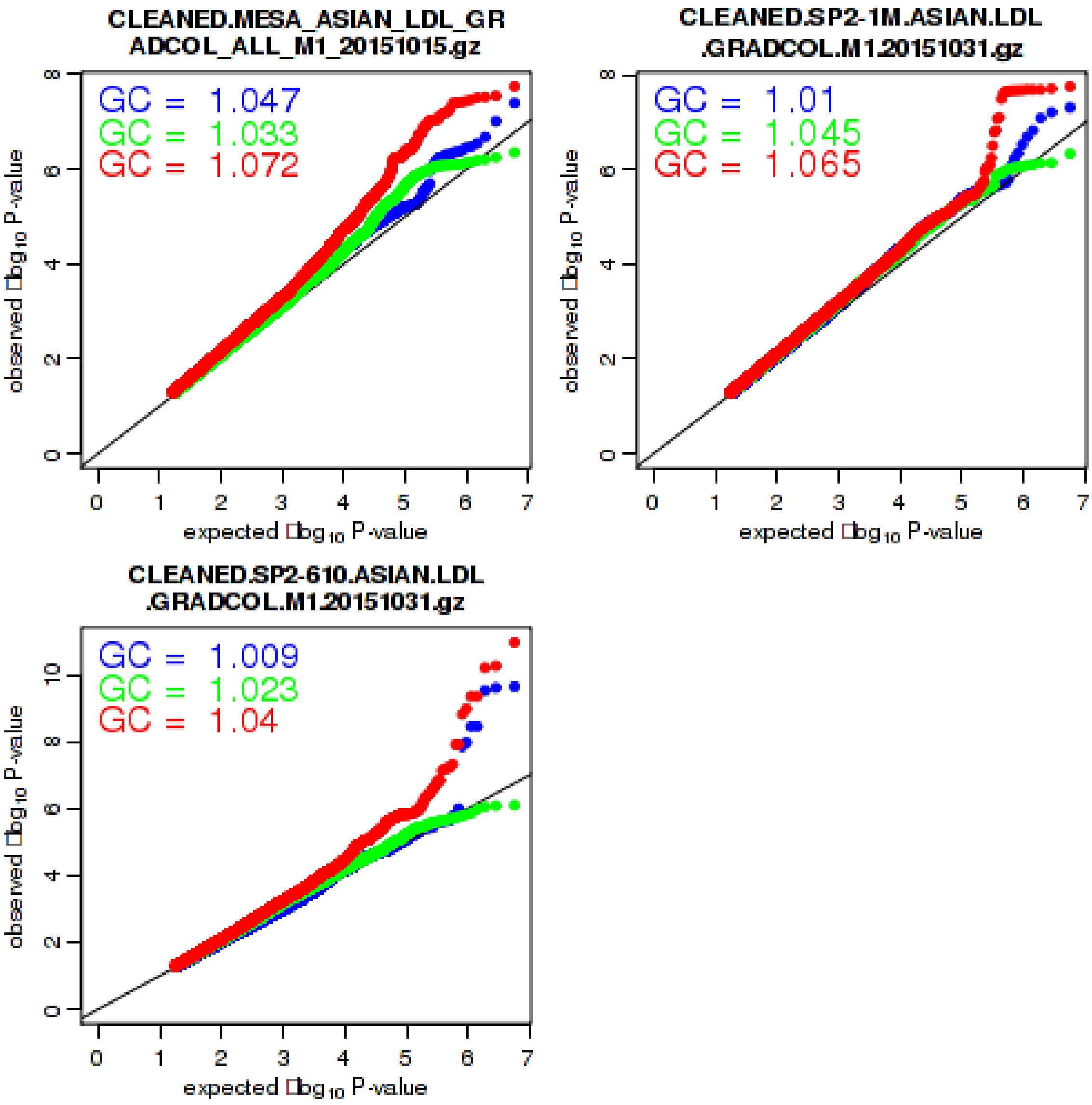

EAS LDL SomeCol M1: DF >20

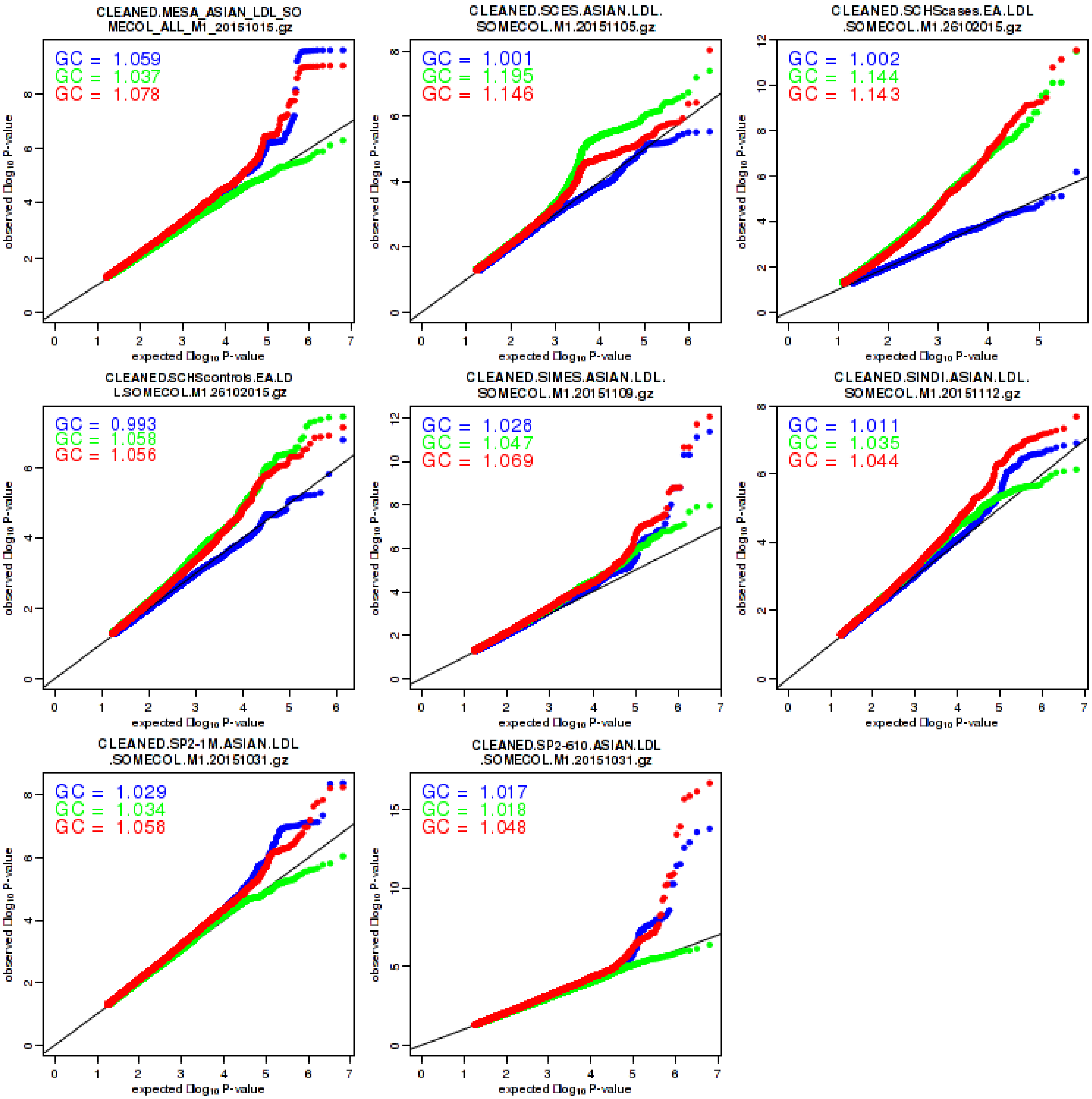

EAS HDL GradCol M1: DF >20

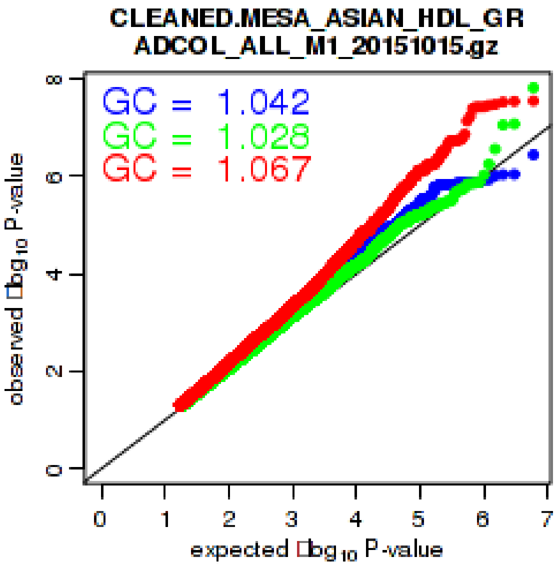

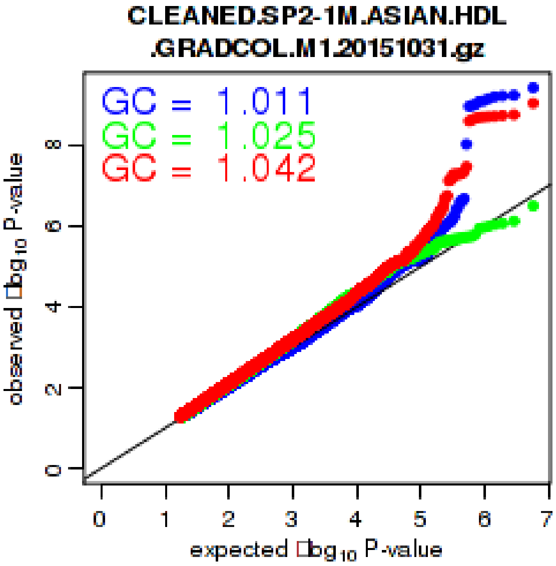

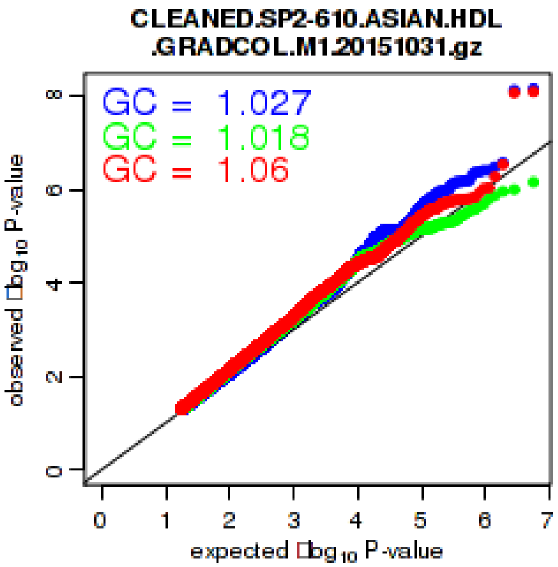

EAS HDL SomeCol M1: DF >20

CLEANED.MESA_ASIAN_HDL_SO
MECOL_ALL_M1_20151015.gz

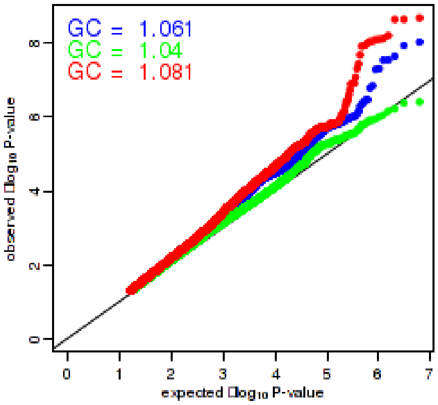

CLEANED.SCES.ASIAN.HDL.
SOMECOL.M1.20151105.gz

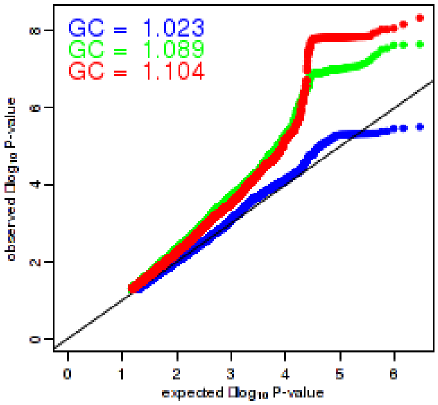

CLEANED.SCHScases.EA.HDL
.SOMECOL.M1.26102015.gz

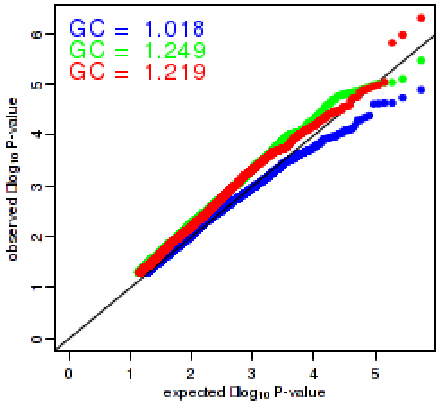

CLEANED.SCHScontrols.EA.HD
L.SOMECOL.M1.26102015.gz

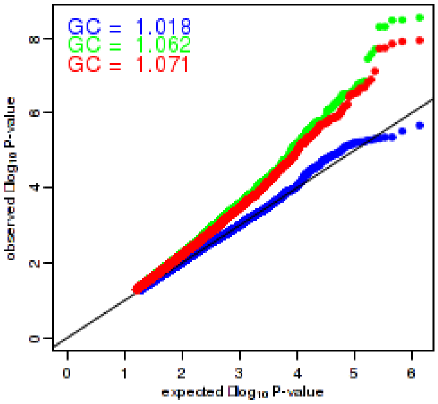

CLEANED.SIMES.ASIAN.HDL.
SOMECOL.M1.20151109.gz

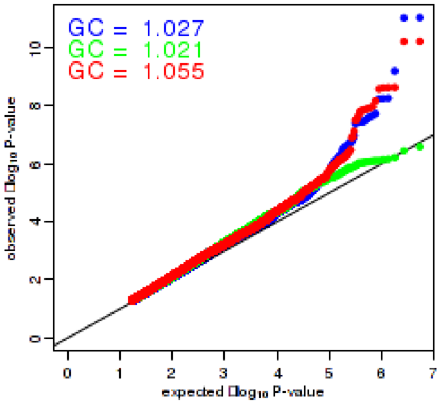

CLEANED.SINDI.ASIAN.HDL.
SOMECOL.M1.20151112.gz

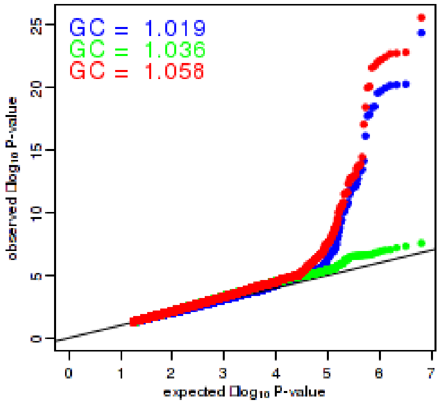

CLEANED.SP2-1M.ASIAN.HDL
.SOMECOL.M1.20151031.gz

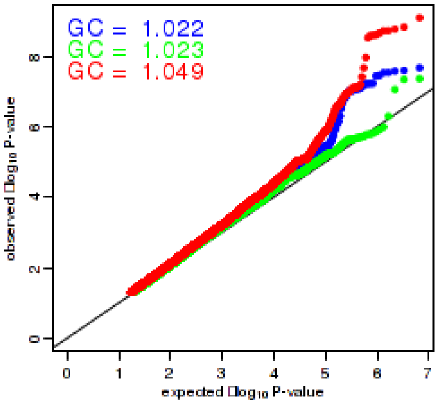

CLEANED.SP2-610.ASIAN.HDL
.SOMECOL.M1.20151031.gz

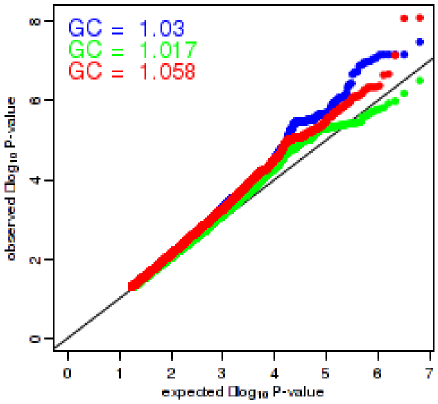

EAS TG GradCol M1: DF >20

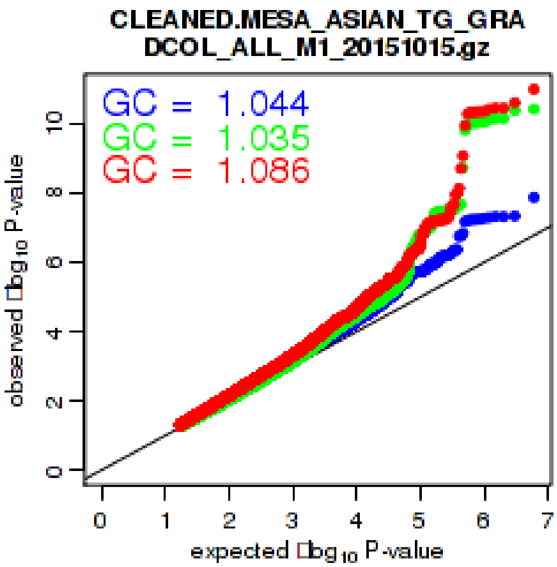

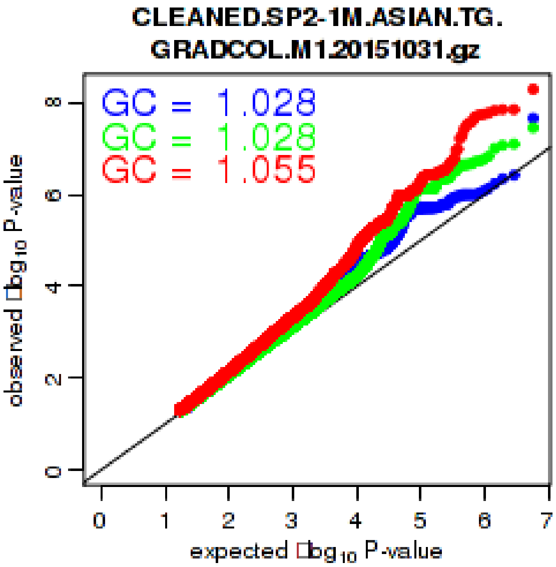

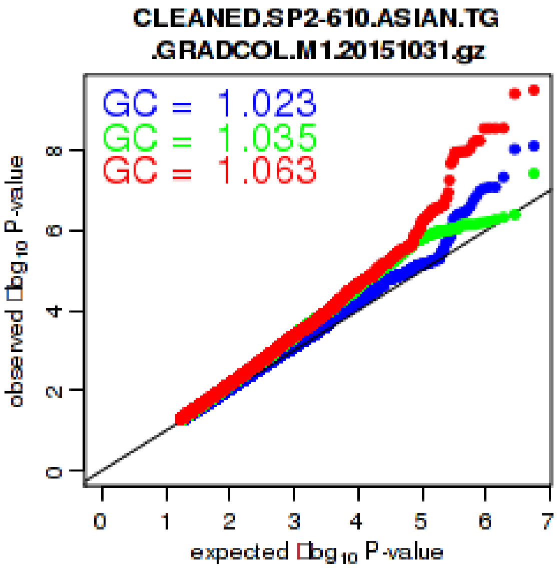

EAS TG SomeCol M1: DF >20

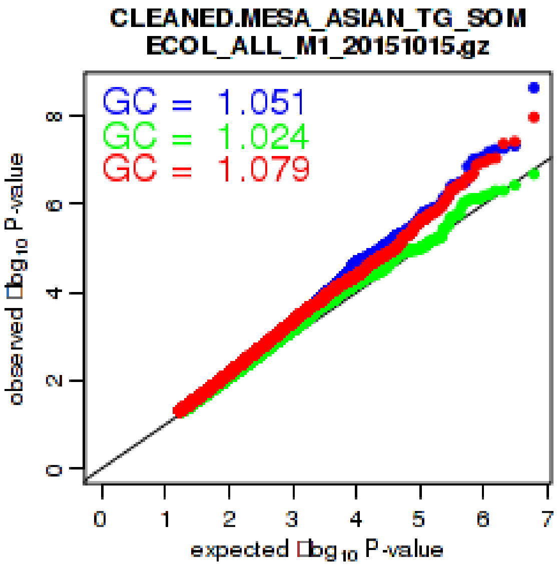

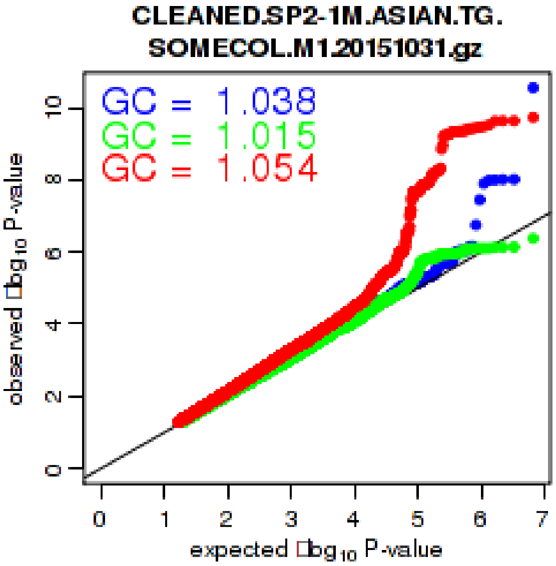

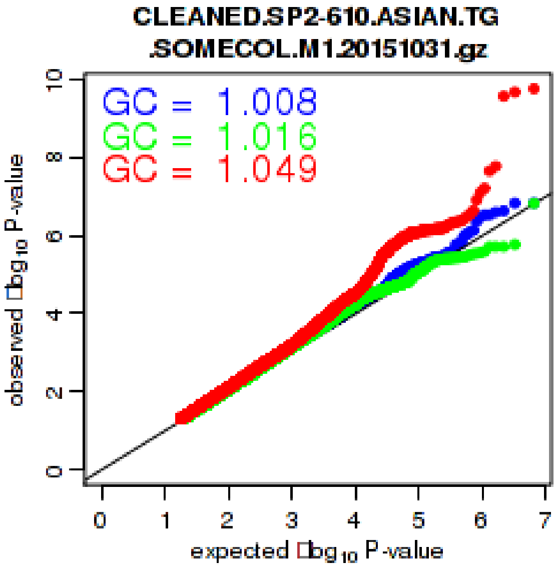

Figure S2C: QQ Plots of Brazilian-Ancestry Cohorts.

<u>BRZ LDL GradCol M1: DF >20</u>

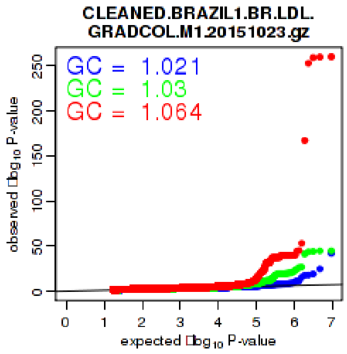

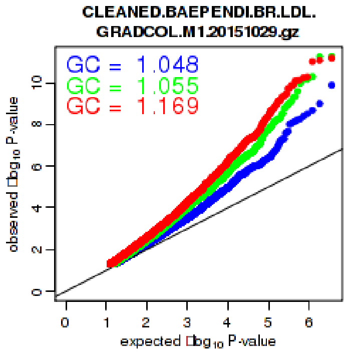

<u>BRZ LDL SomeCol M1: DF >20</u>

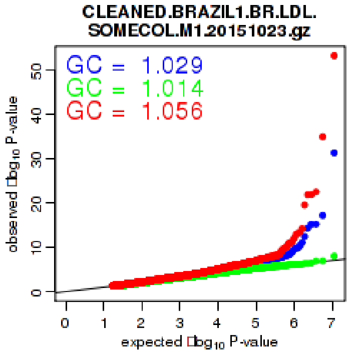

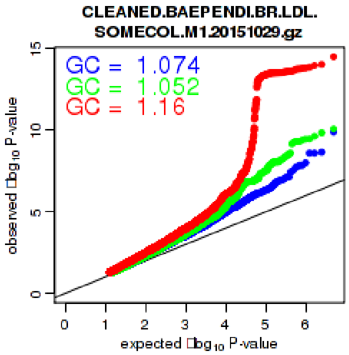

BRZ HDL GradCol M1: DF >20

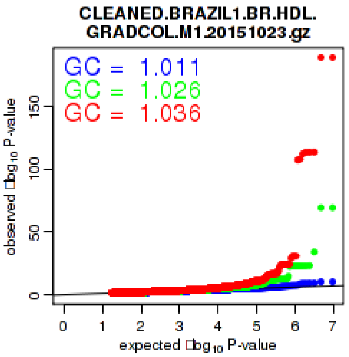

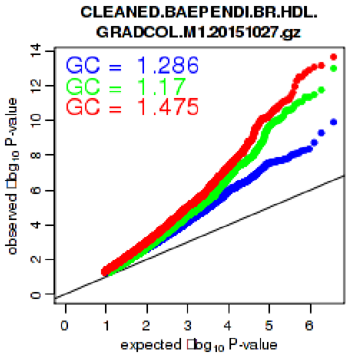

BRZ HDL SomeCol M1: DF >20

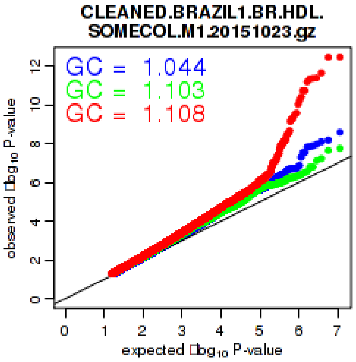

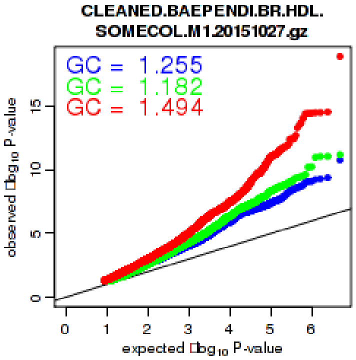

BRZ TG GradCol M1: DF >20

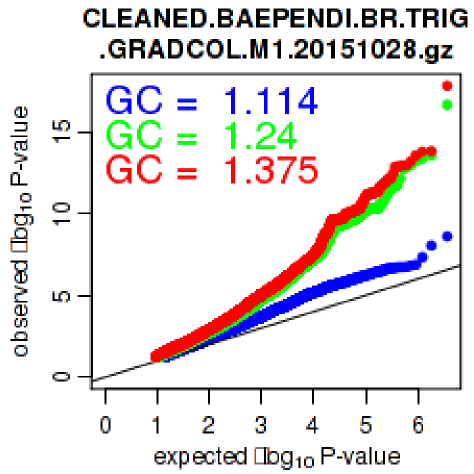

BRZ TG SomeCol M1: DF >20

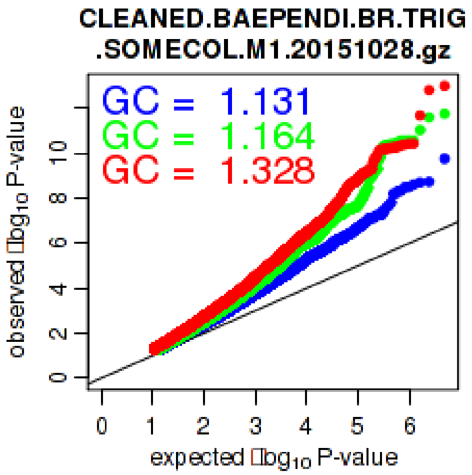

Figure S2D: QQ Plots of European-Ancestry Cohorts.

EUR LDL GradCol M1: DF >20

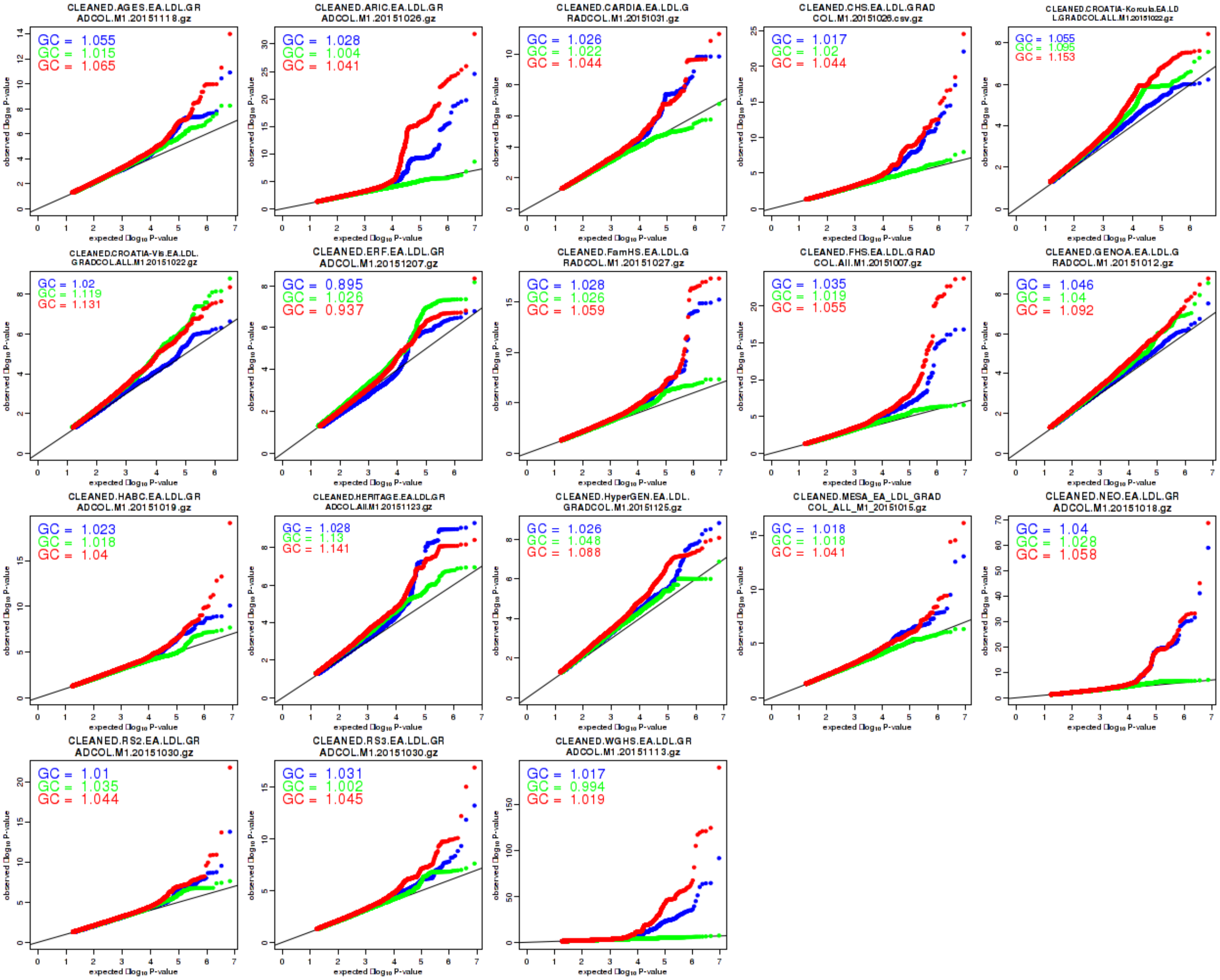

EUR LDL SomeCol M1: DF >20

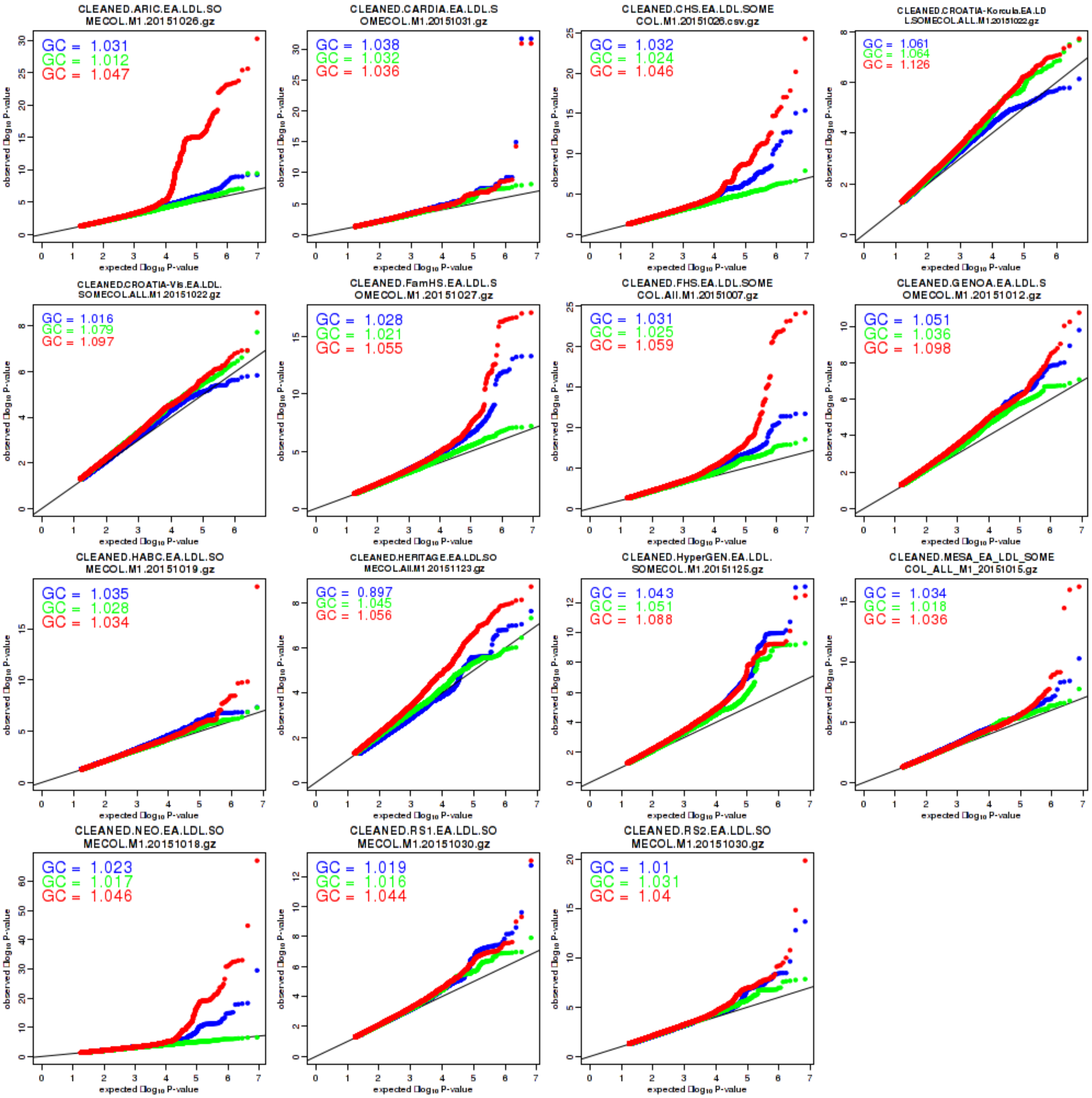

<u>**EUR HDL GradCol M1: DF >20**</u>

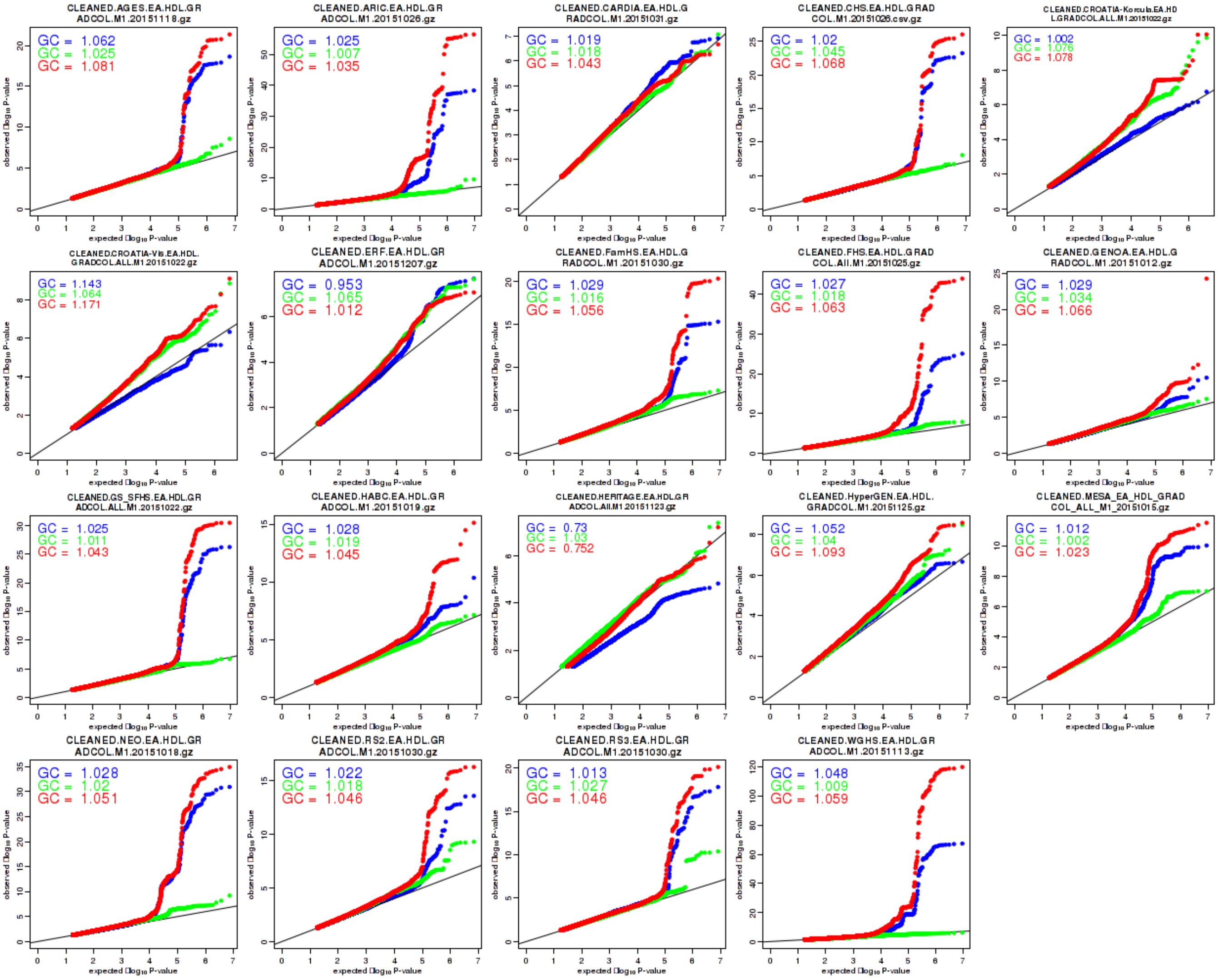

EUR HDL SomeCol M1: DF >20

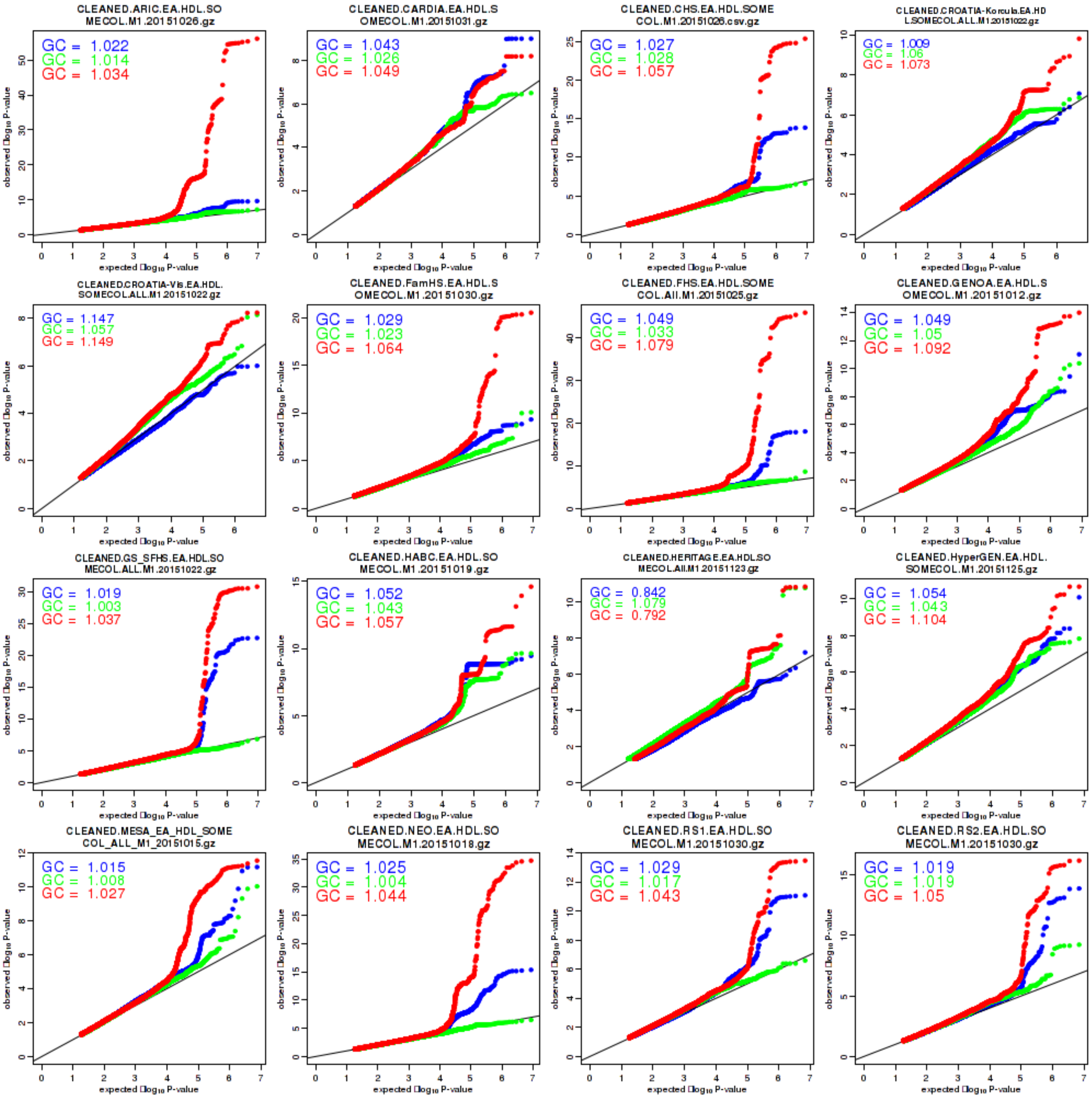

EUR TG GradCol M1: DF >20

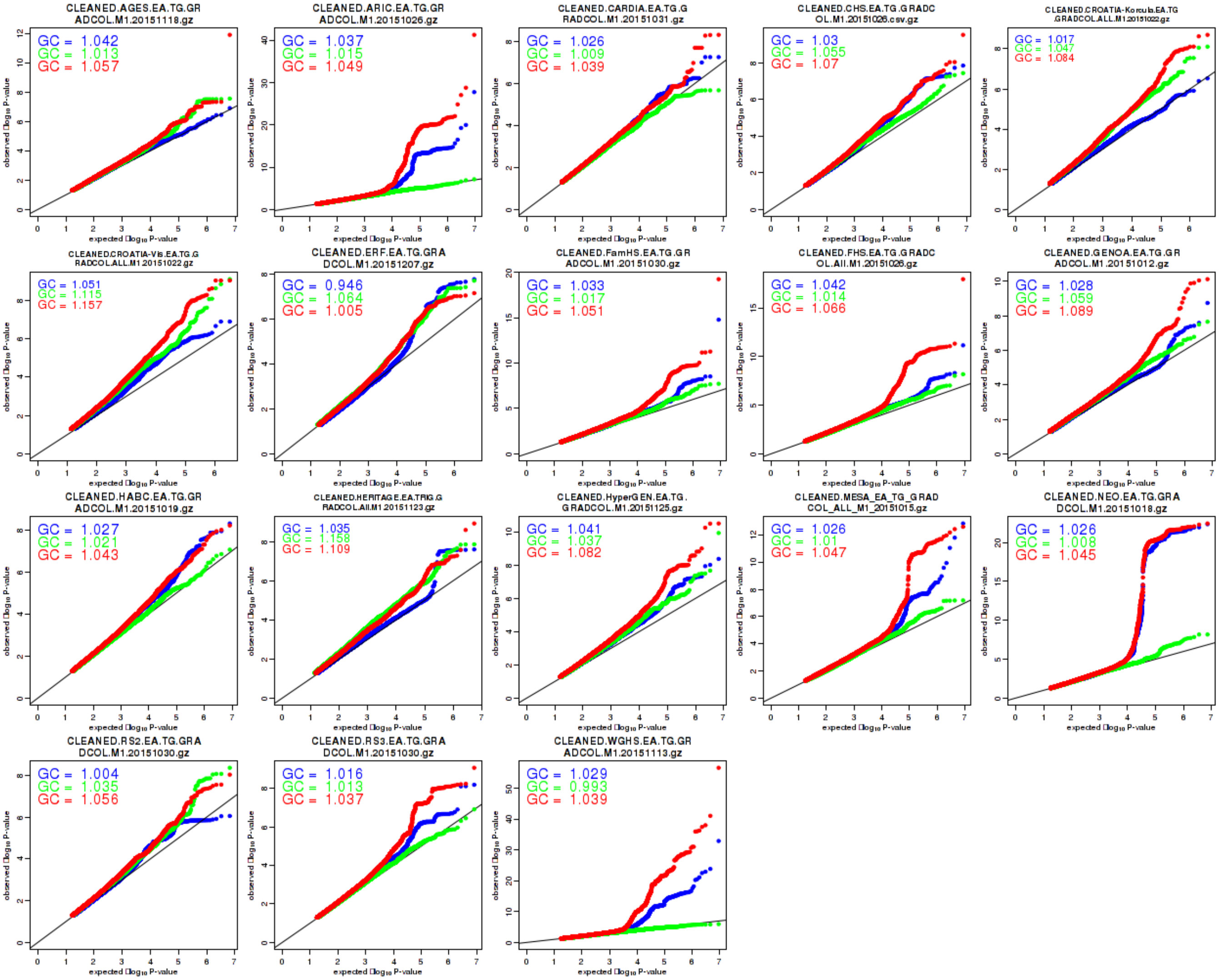

EUR TG SomeCol M1: DF >20

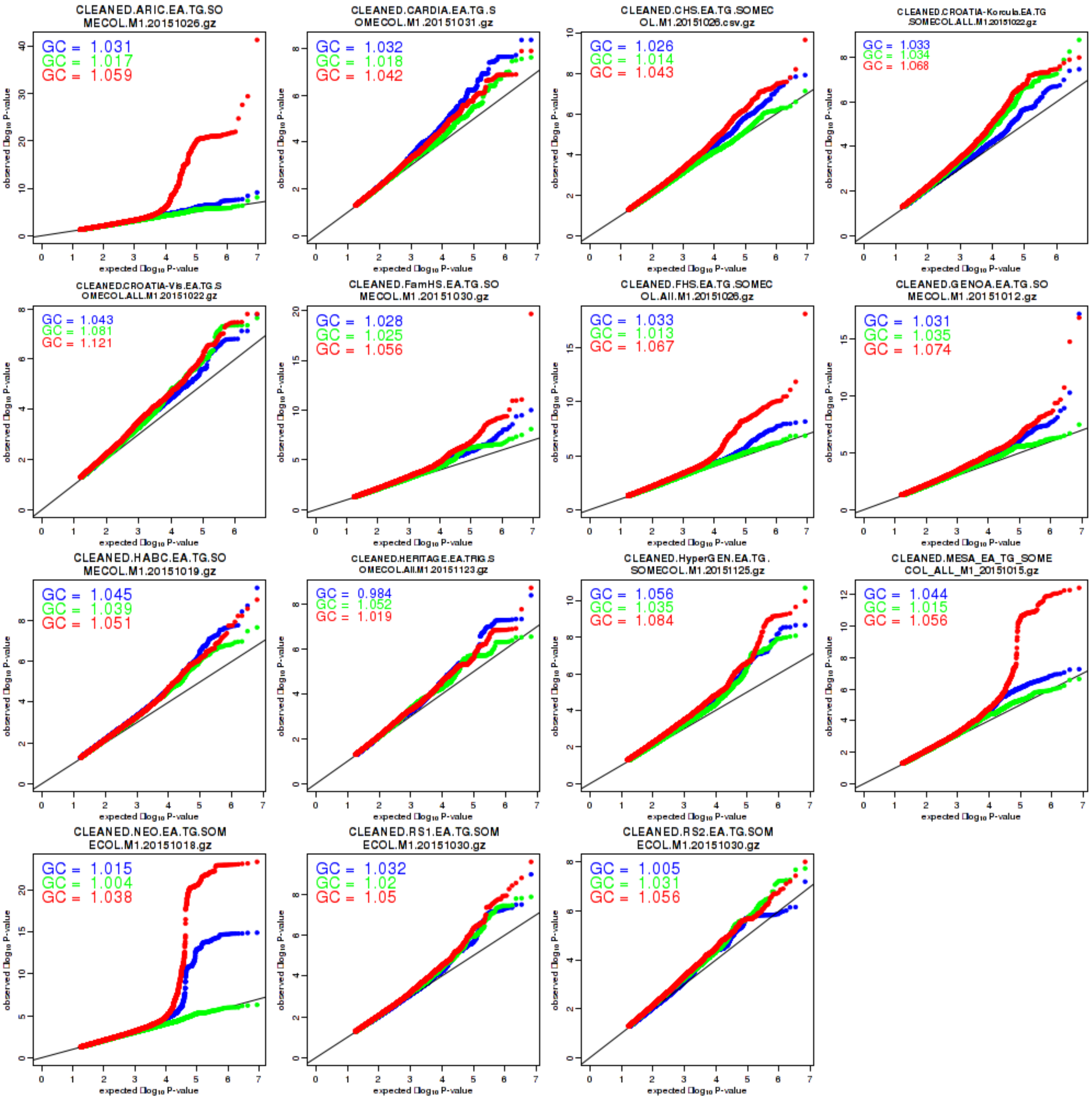

Figure S2E: QQ Plots of Hispanic-Ancestry Cohorts.

HIS LDL GradCol M1: DF >20

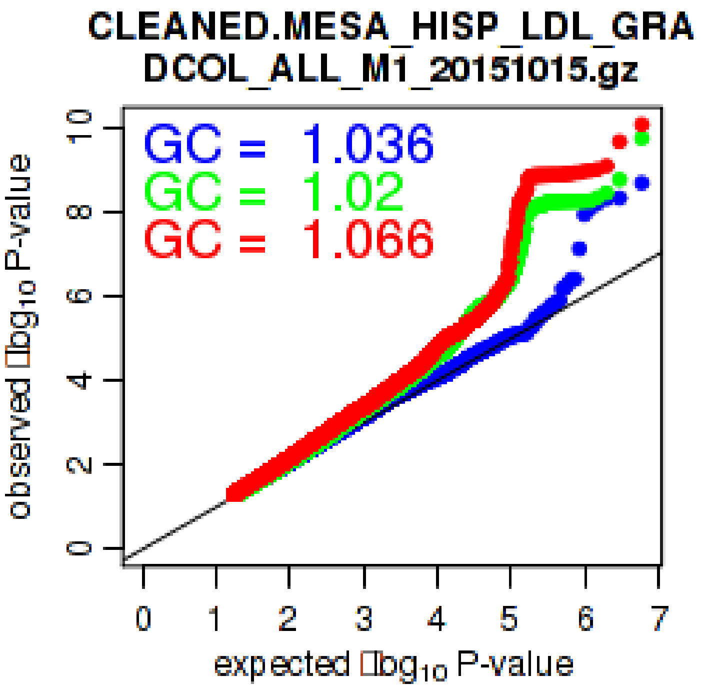

HIS LDL SomeCol M1: DF >20

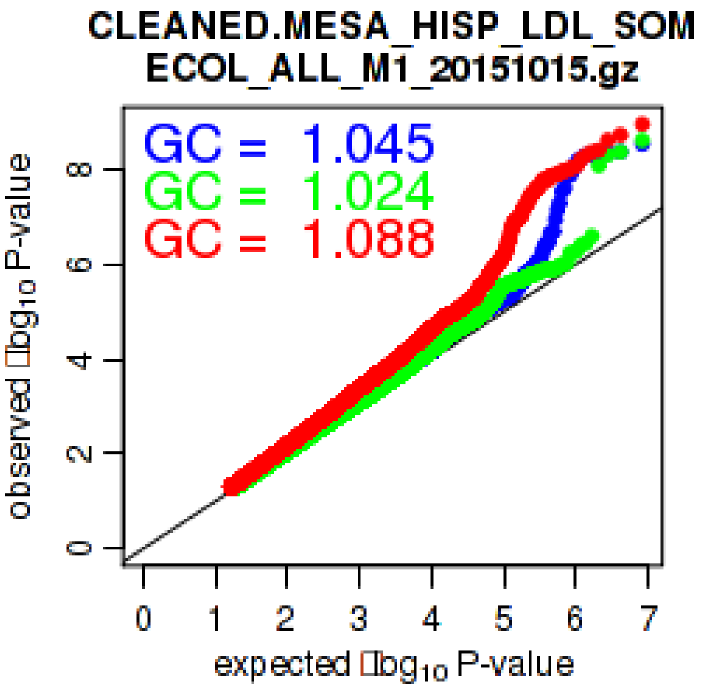

HIS HDL GradCol M1: DF >20

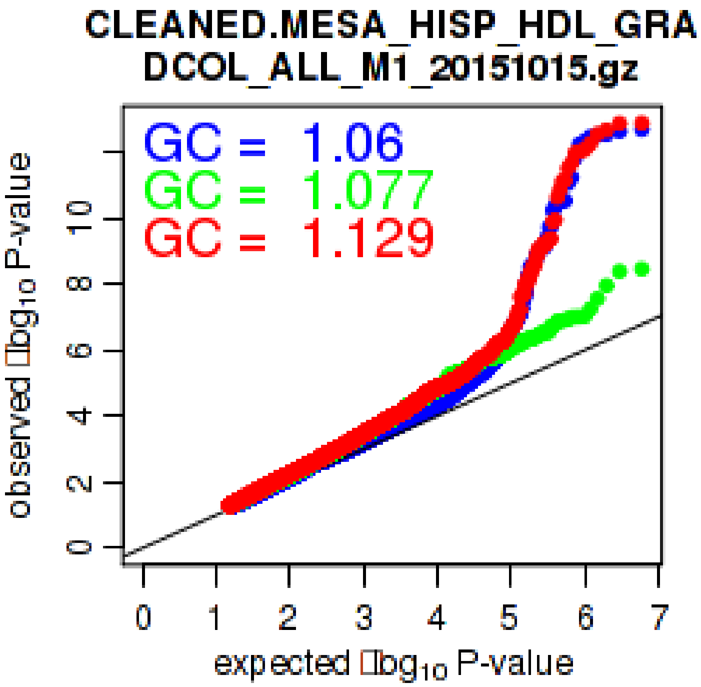

HIS HDL SomeCol M1: DF >20

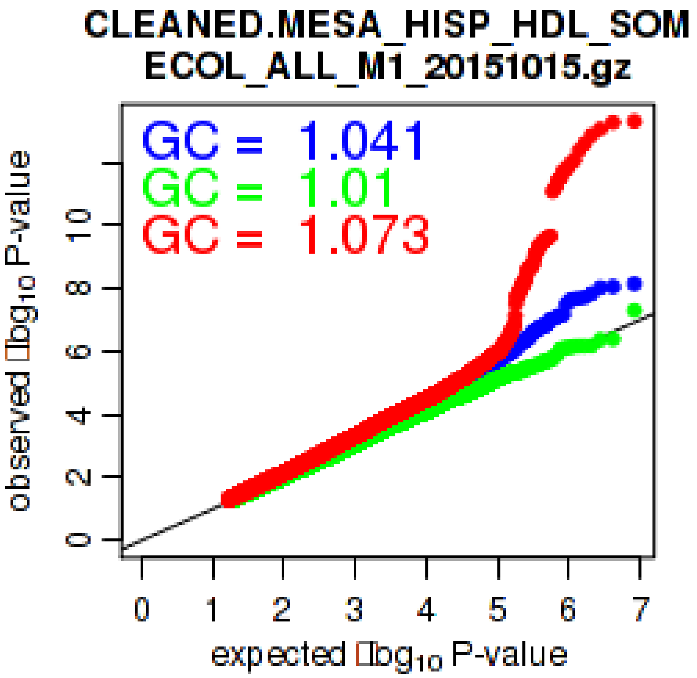

HIS TG GradCol M1: DF >20

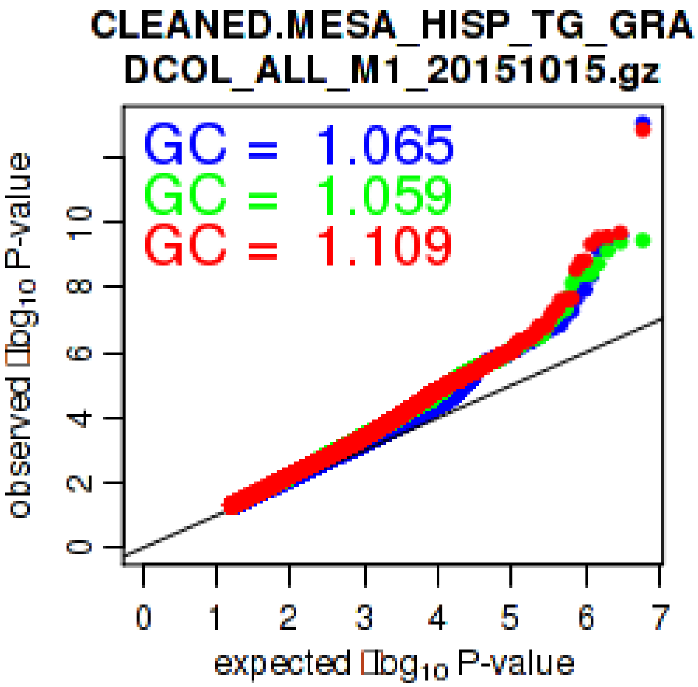

HIS TG SomeCol M1: DF >20

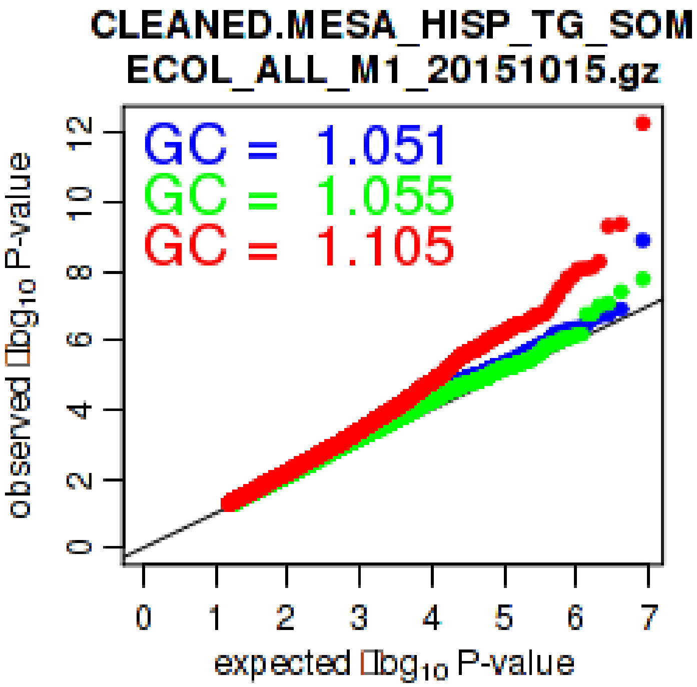

Figure S3A: Manhattan plots of the combined analyses of Stages 1 and 2 using the 2DF for LDL and GradCol by ancestry.

The -log10(P) of each SNV was plotted at the chromosomal location of each variant. P-values are based on the meta-analysis combining Stages 1 and 2 analysis and the Stage 1 analysis for the remaining 18.8 million variants.

African Ancestry

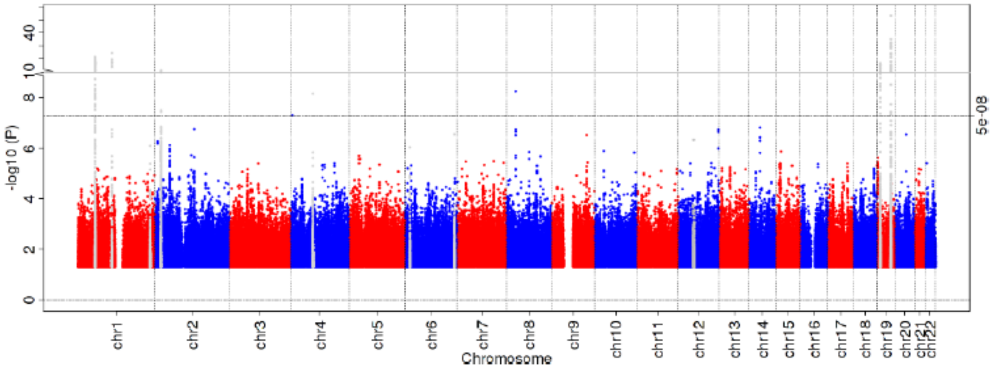

Asian Ancestry

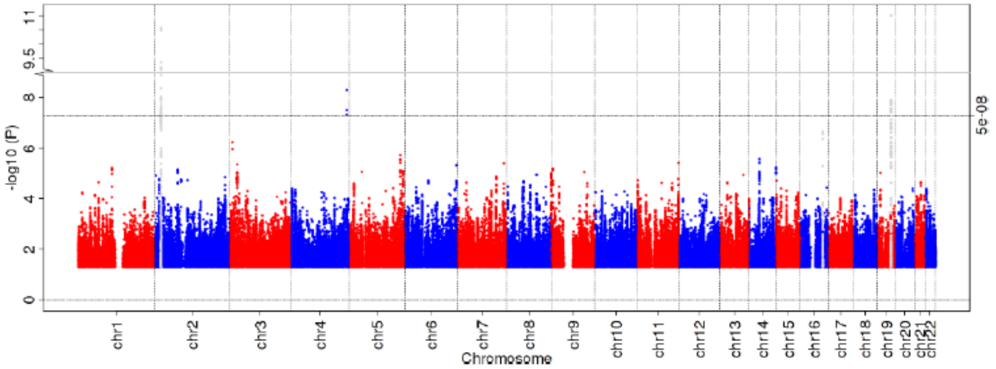

Brazilian Ancestry

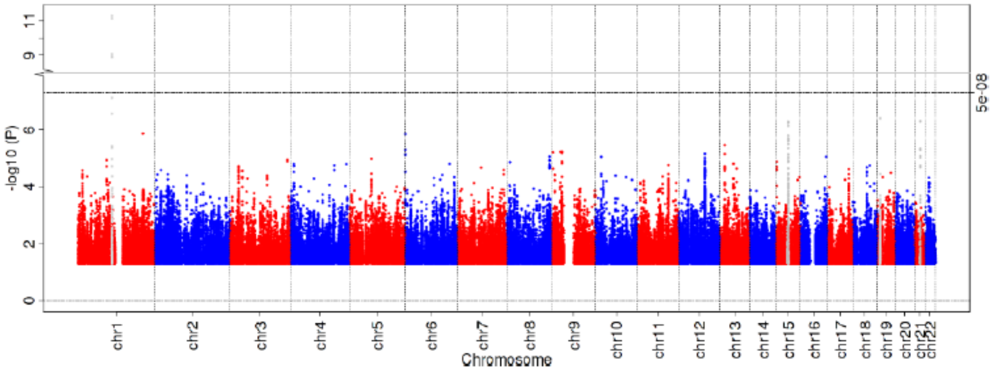

European Ancestry

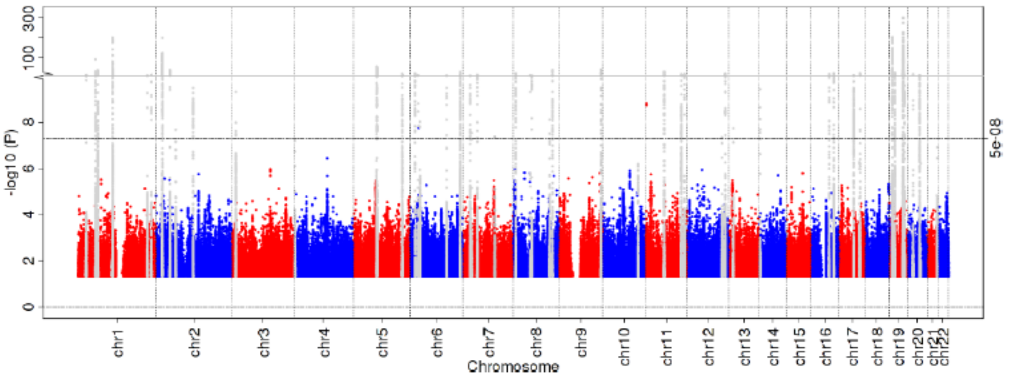

Hispanic Ancestry

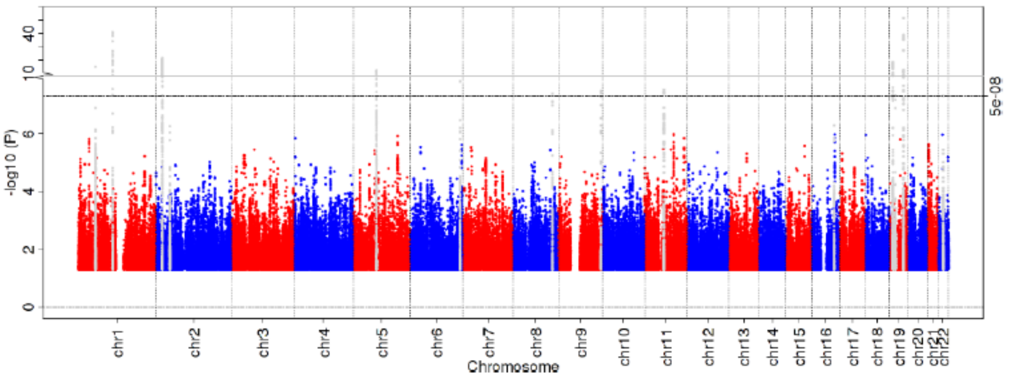

Cross-Population Analysis

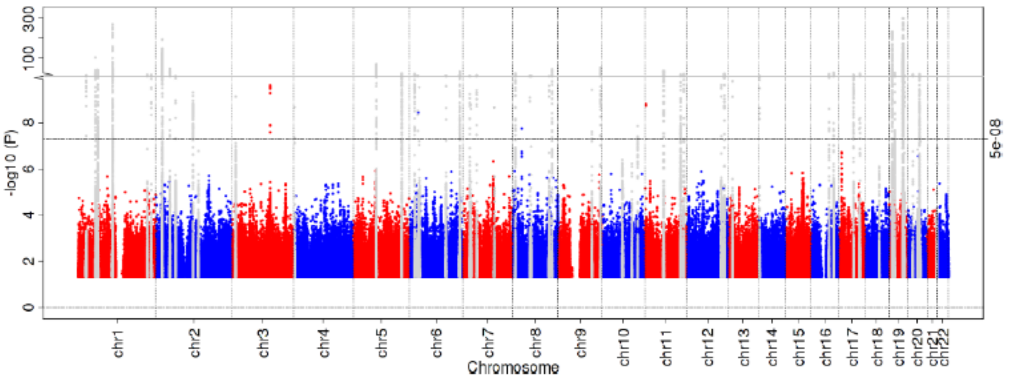

Figure S3B: Manhattan plots of the combined analyses of Stages 1 and 2 using the 2DF for LDL and SomeCol by ancestry.

The -log10(P) of each SNV was plotted at the chromosomal location of each variant. P-values are based on the meta-analysis combining Stages 1 and 2 analysis and the Stage 1 analysis for the remaining 18.8 million variants.

African Ancestry

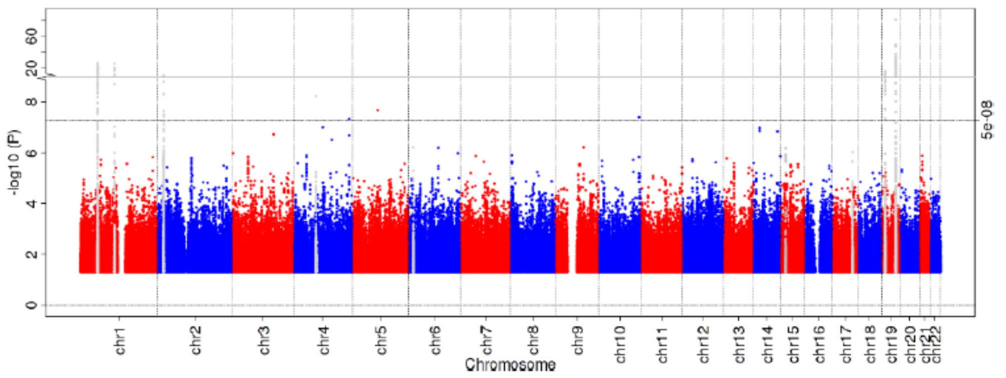

Asian Ancestry

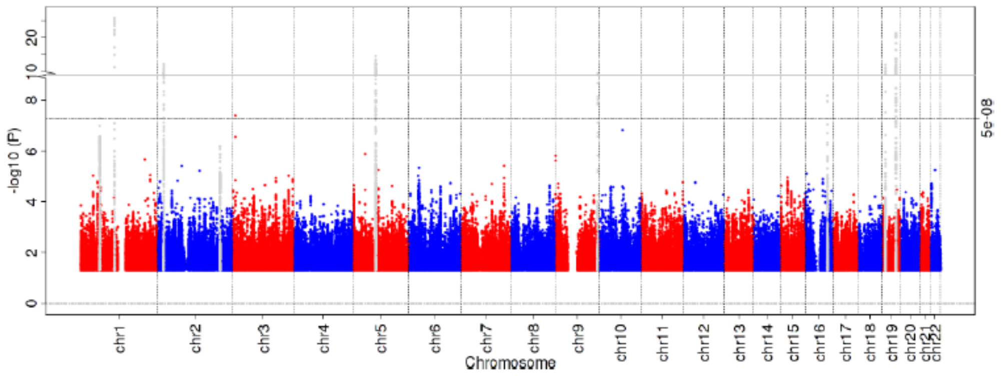

Brazilian Ancestry

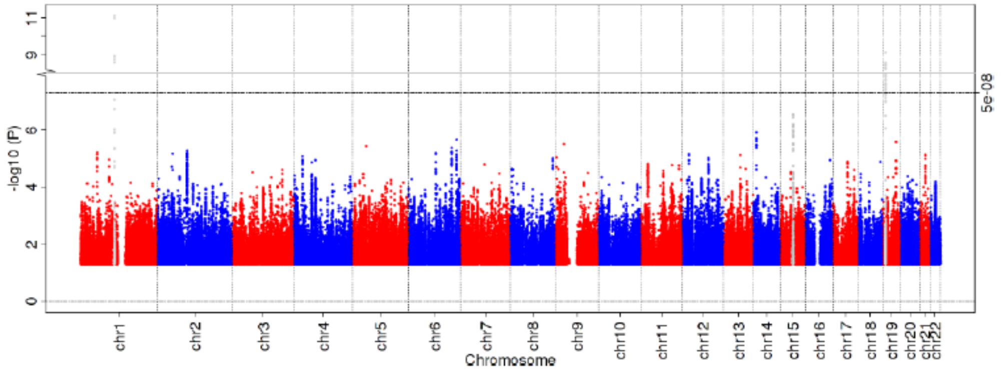

European Ancestry

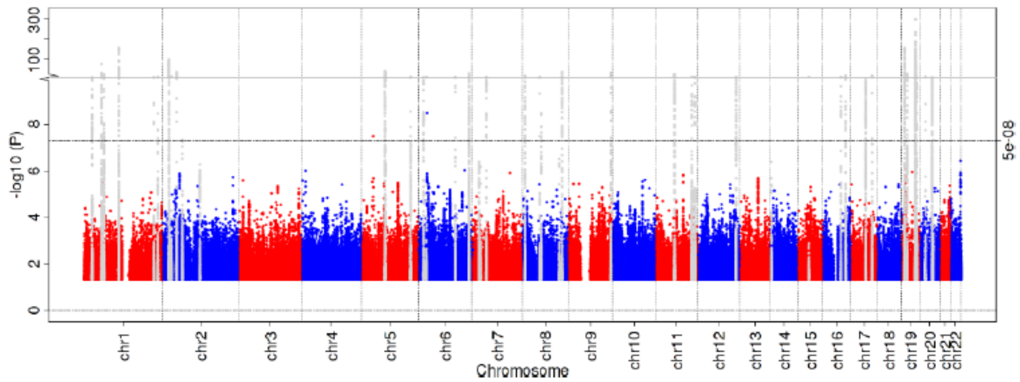

Hispanic Ancestry

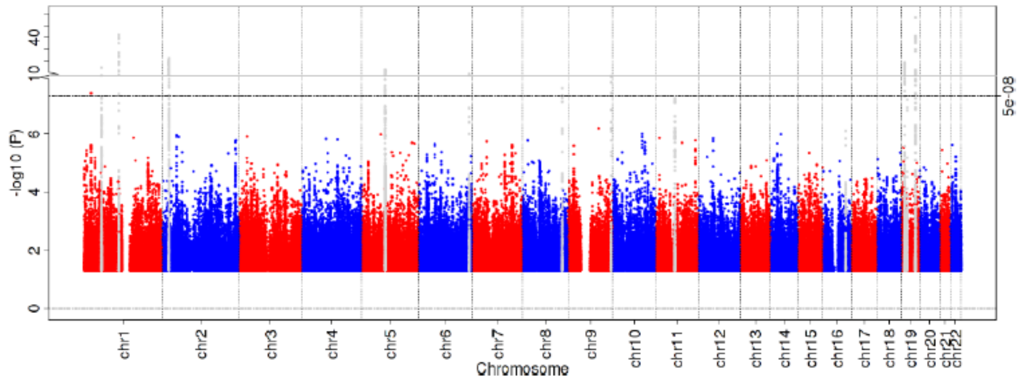

Cross-Population Analysis

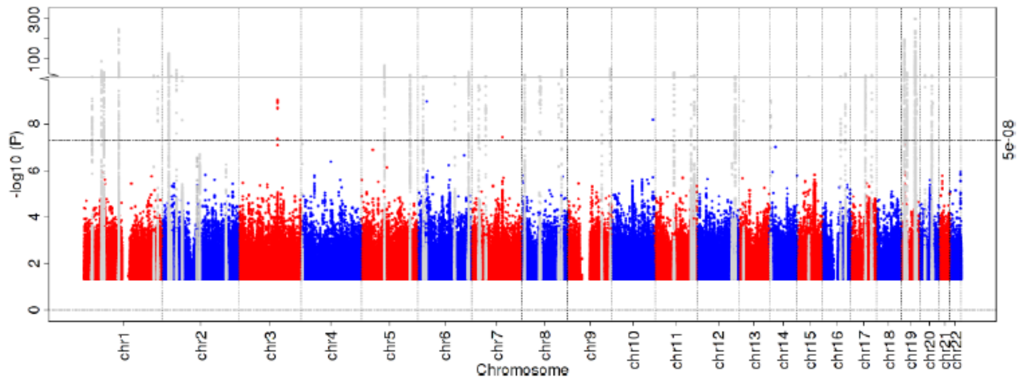

Figure S3C: Manhattan plots of the combined analyses of Stages 1 and 2 using the 2DF for HDL and GradCol by ancestry.

The -log10(P) of each SNV was plotted at the chromosomal location of each variant. P-values are based on the meta-analysis combining Stages 1 and 2 analysis and the Stage 1 analysis for the remaining 18.8 million variants.

<u>African Ancestry</u>

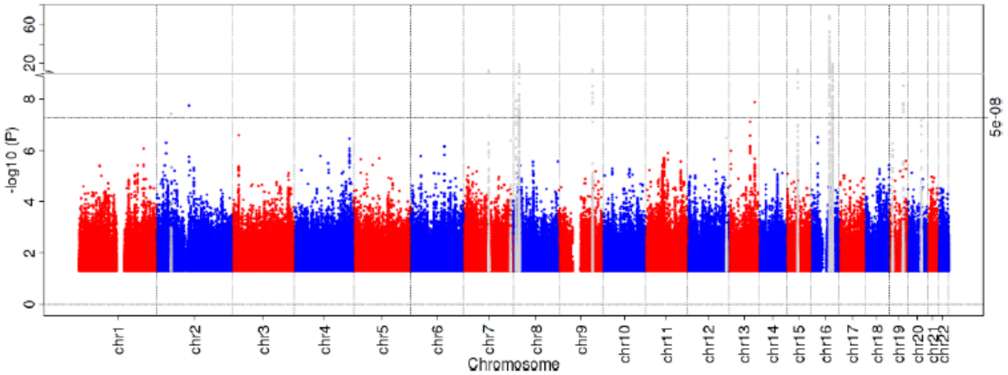

<u>Asian Ancestry</u>

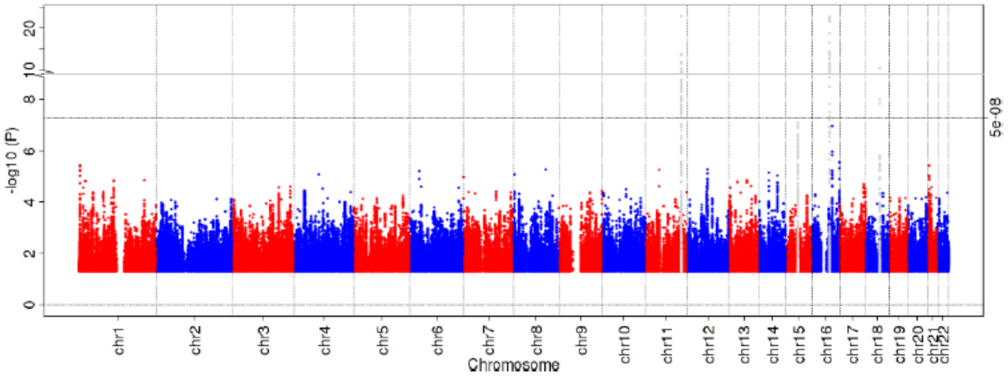

<u>Brazilian Ancestry</u>

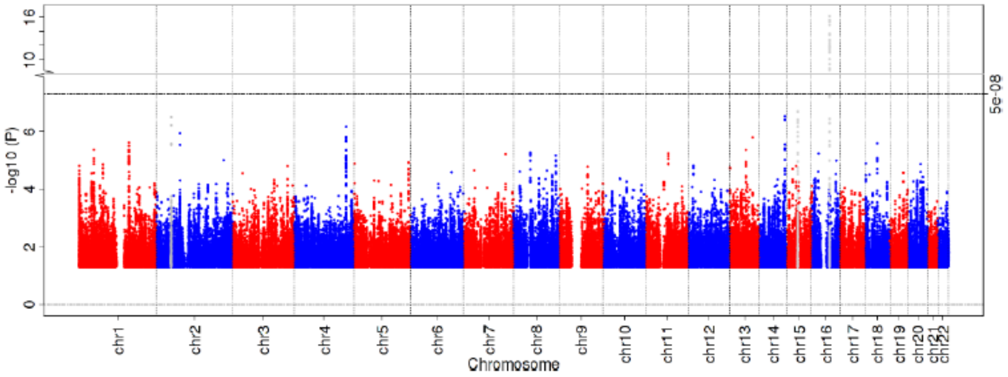

European Ancestry

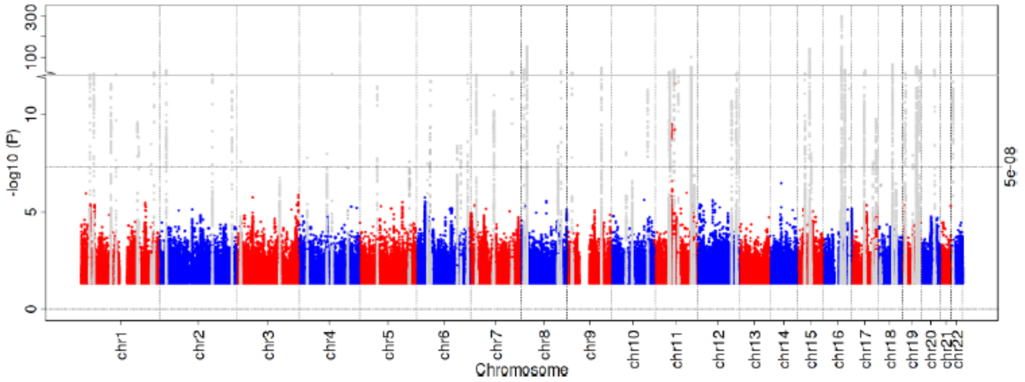

Hispanic Ancestry

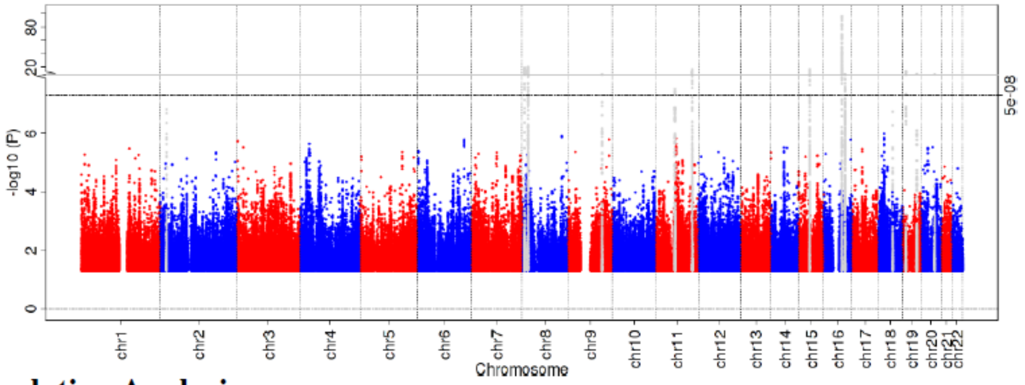

Cross-Population Analysis

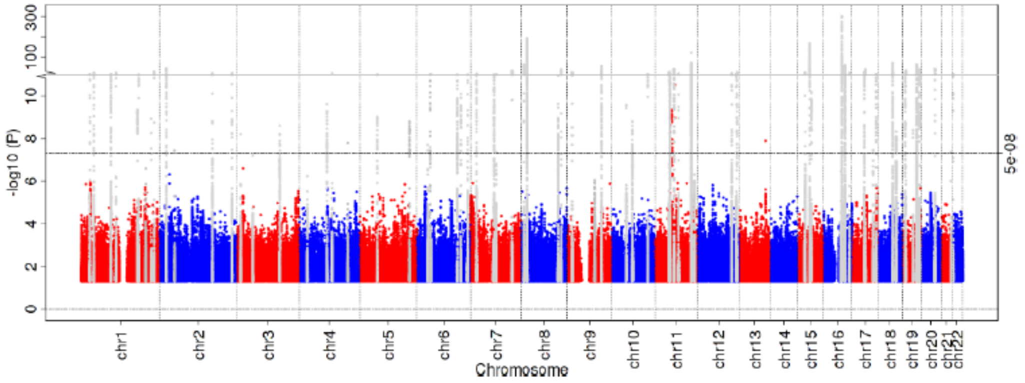

Figure S3D: Manhattan plots of the combined analyses of Stages 1 and 2 using the 2DF for HDL and SomeCol by ancestry.

The -log10(P) of each SNV was plotted at the chromosomal location of each variant. P-values are based on the meta-analysis combining Stages 1 and 2 analysis and the Stage 1 analysis for the remaining 18.8 million variants.

African Ancestry

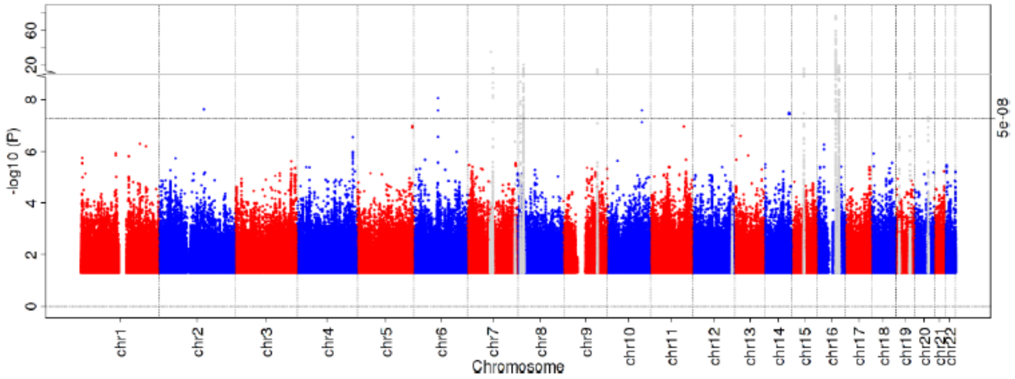

Asian Ancestry

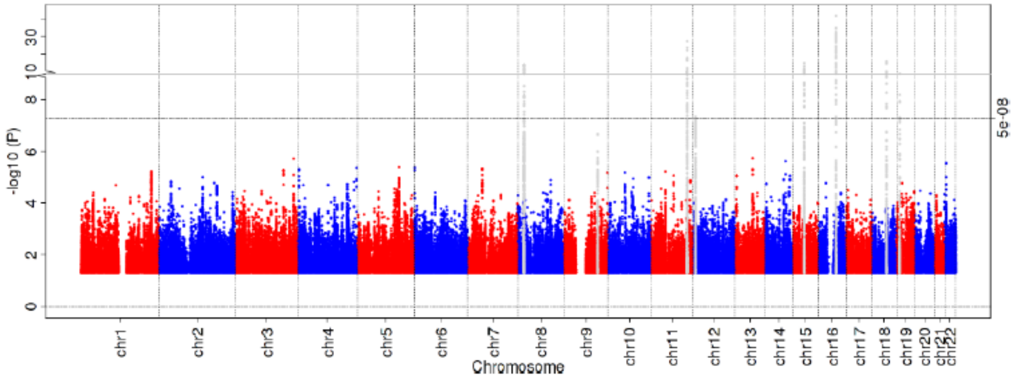

Brazilian Ancestry

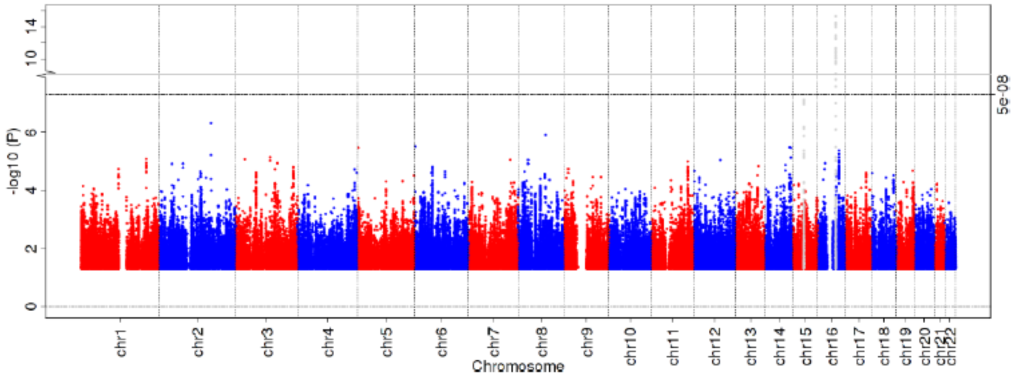

European Ancestry

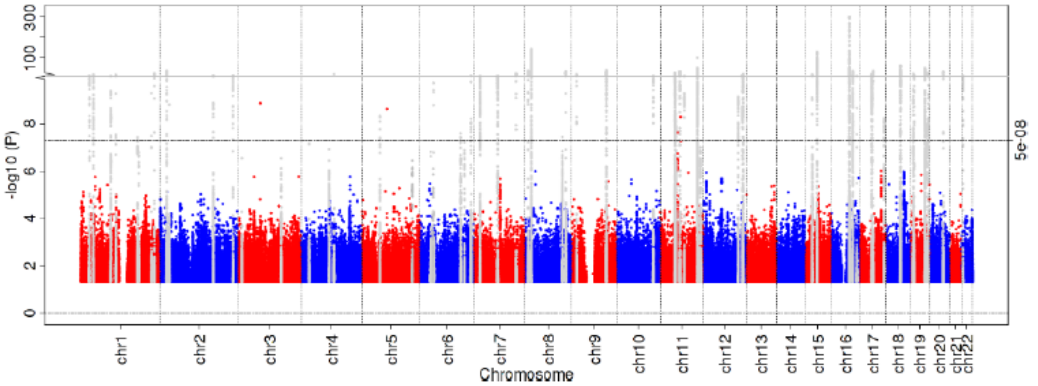

Hispanic Ancestry

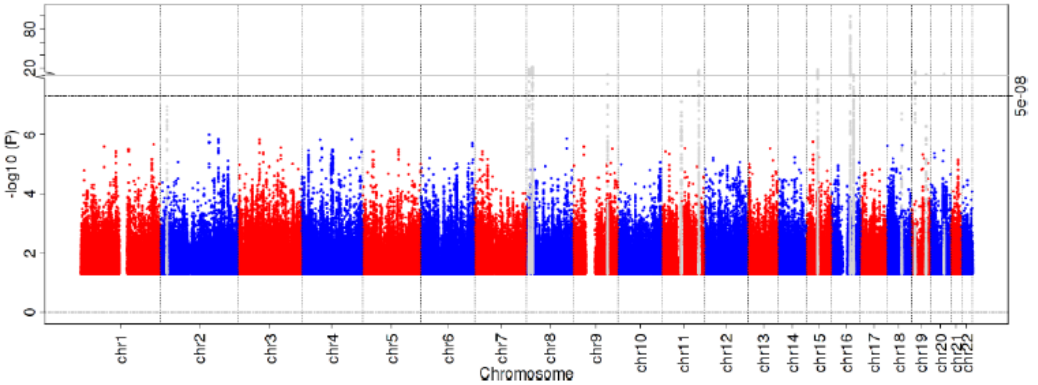

Cross-Population Analysis

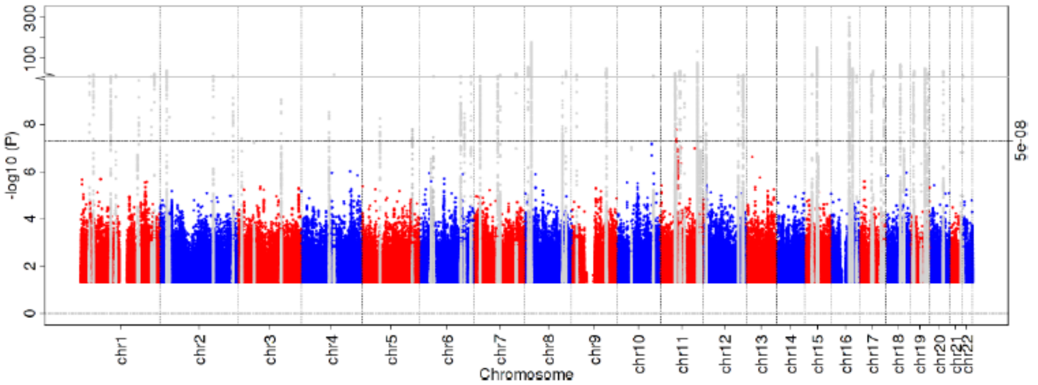

Figure S3E: Manhattan plots of the combined analyses of Stages 1 and 2 using the 2DF for TG and GradCol by ancestry.

The -log10(P) of each SNV was plotted at the chromosomal location of each variant. P-values are based on the meta-analysis combining Stages 1 and 2 analysis and the Stage 1 analysis for the remaining 18.8 million variants.

African Ancestry

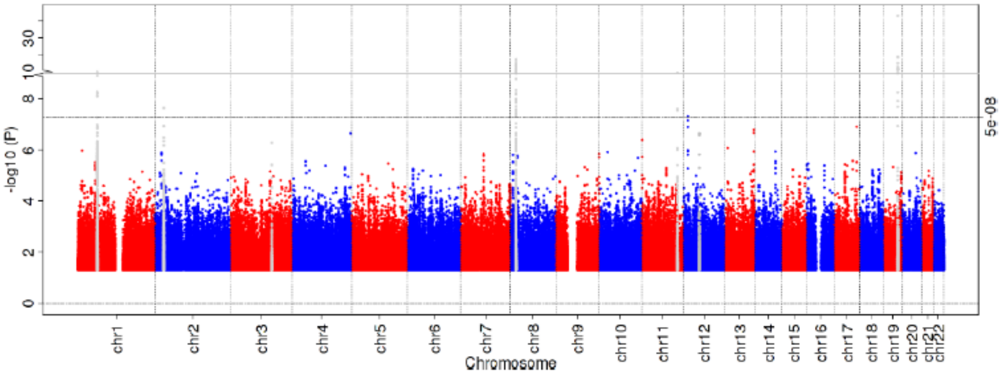

Asian Ancestry

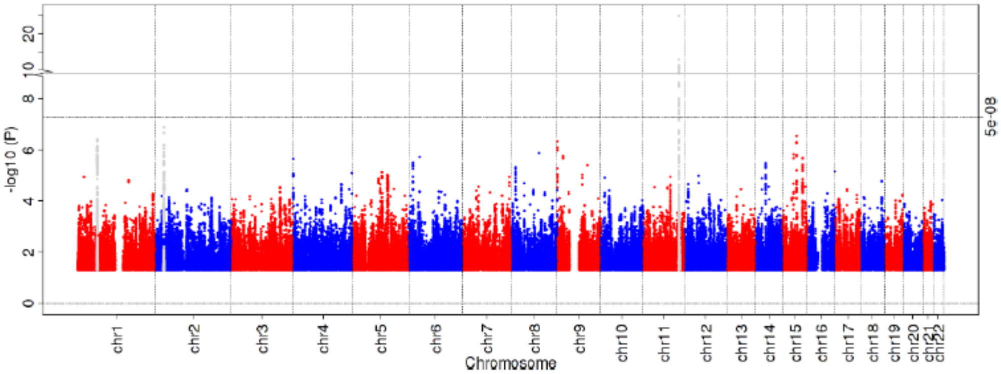

European Ancestry

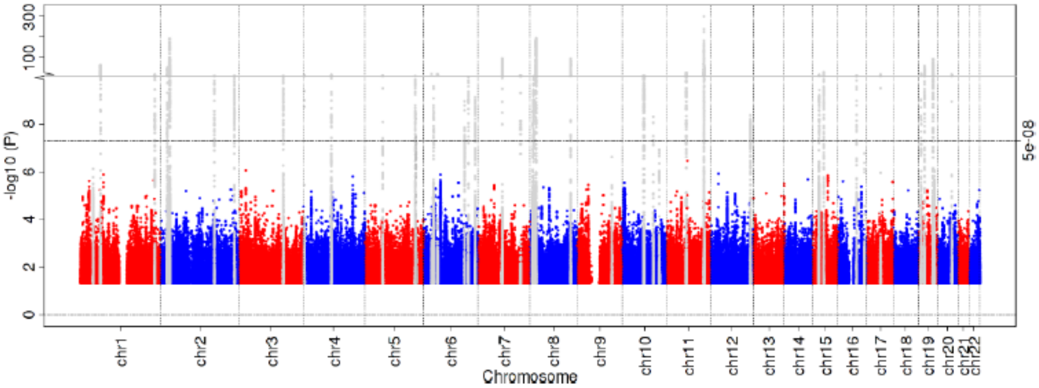

Hispanic Ancestry

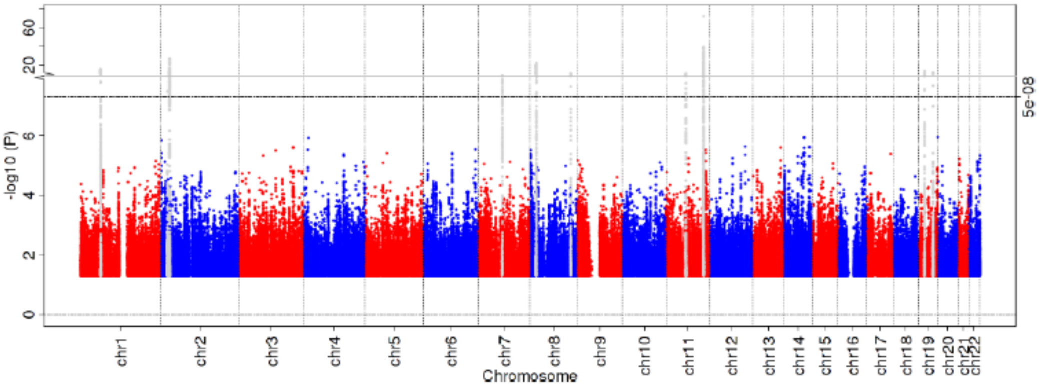

Cross-Population Analysis

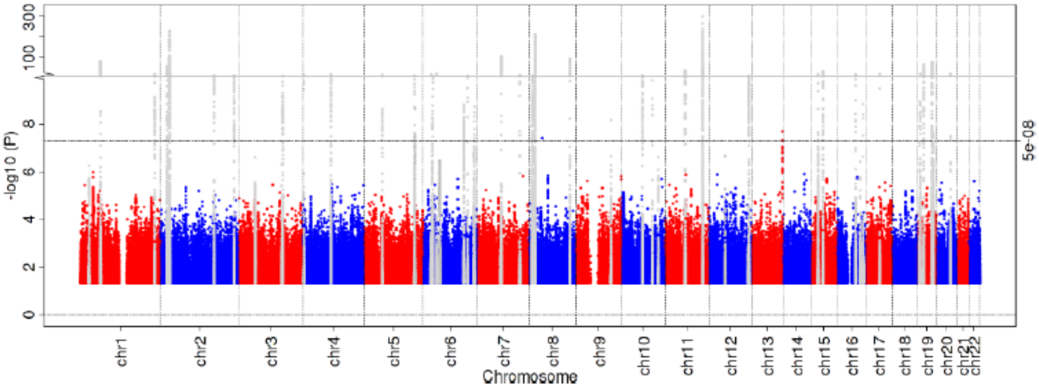

Figure S3F: Manhattan plots of the combined analyses of Stages 1 and 2 using the 2DF for TG and SomeCol by ancestry.

The -log10(P) of each SNV was plotted at the chromosomal location of each variant. P-values are based on the meta-analysis combining Stages 1 and 2 analysis and the Stage 1 analysis for the remaining 18.8 million variants.

African Ancestry

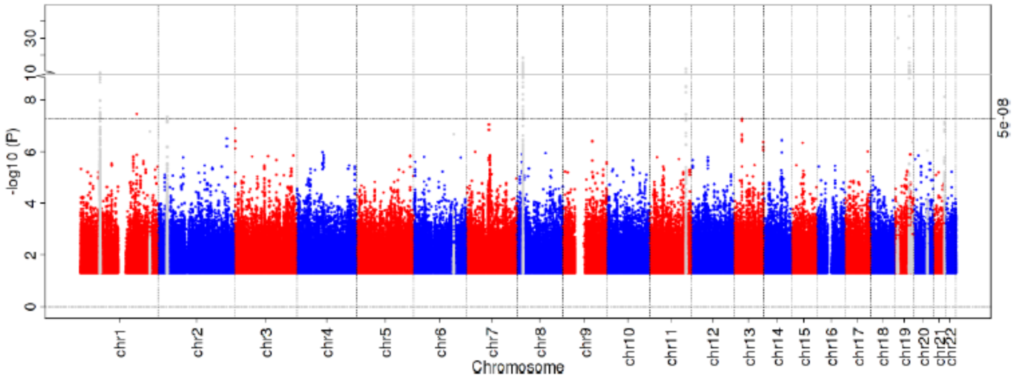

Asian Ancestry

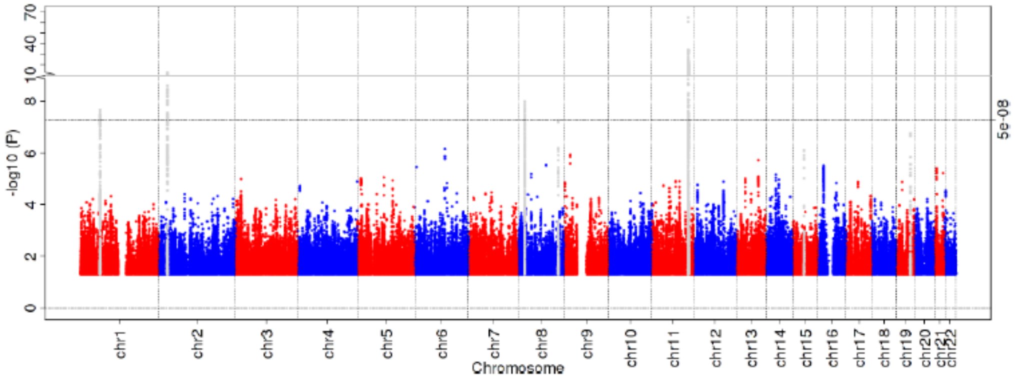

European Ancestry

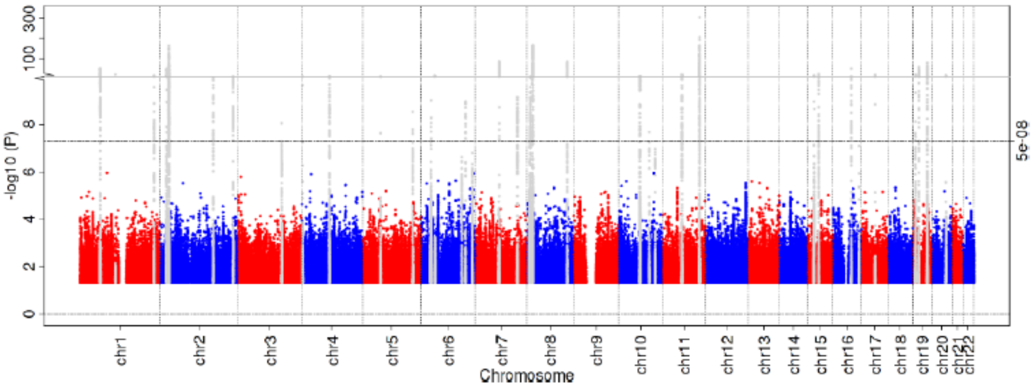

Hispanic Ancestry

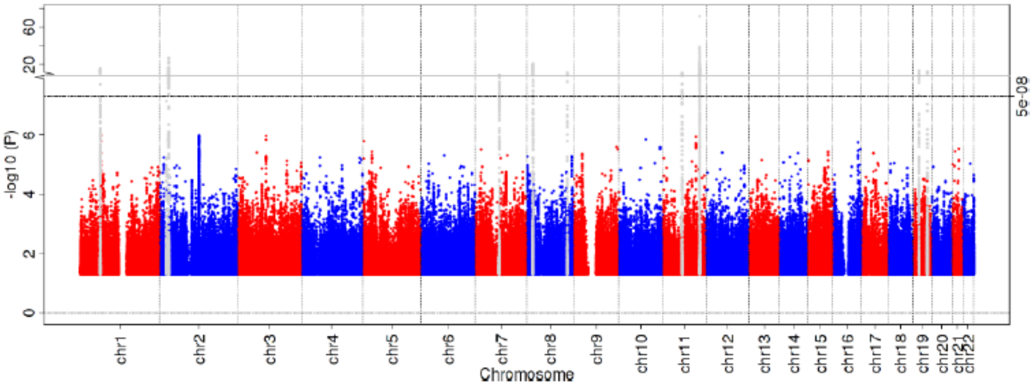

Cross-Population Analysis

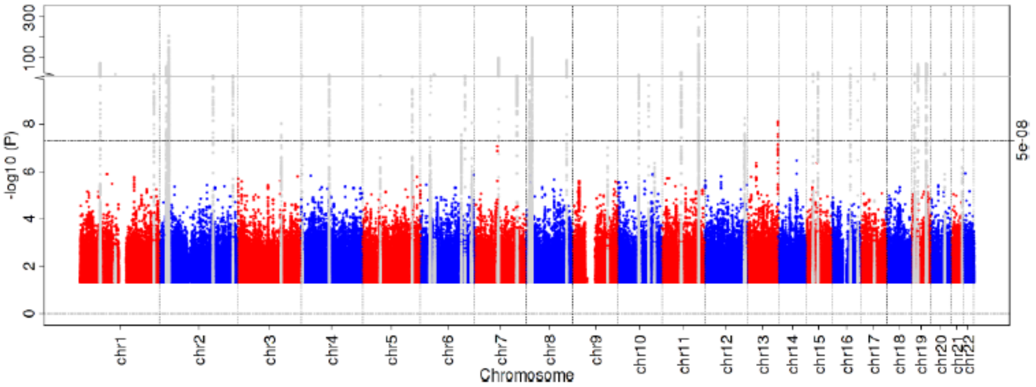

Figure S4: LocusZoom plots for 18 newly identified loci (Table 1).

Each locus has at least one lipid trait reaching genome-wide significance with at least one lipid trait in any ancestry group. LD substructure is not available for six insertion/deletion index variants.

Locus 1: HDL-GradCol in African Ancestry (2DF).

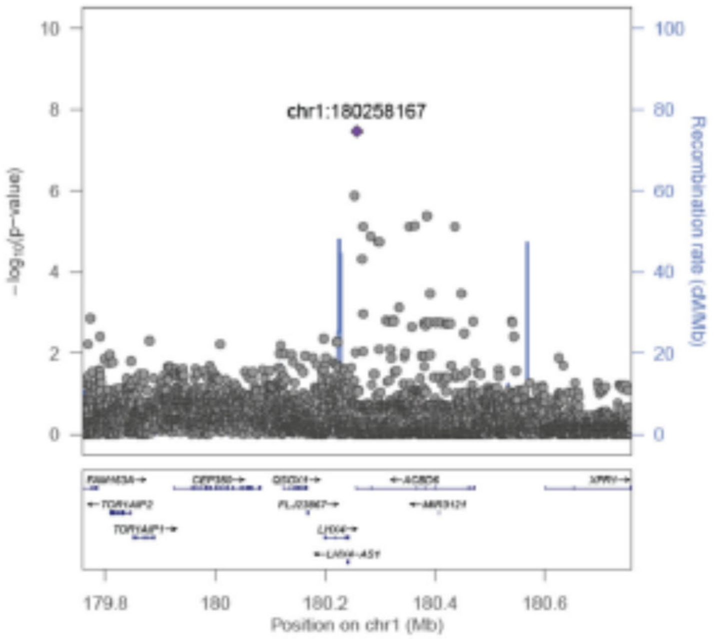

Locus 2: TG-SomeCol in African Ancestry (2DF).

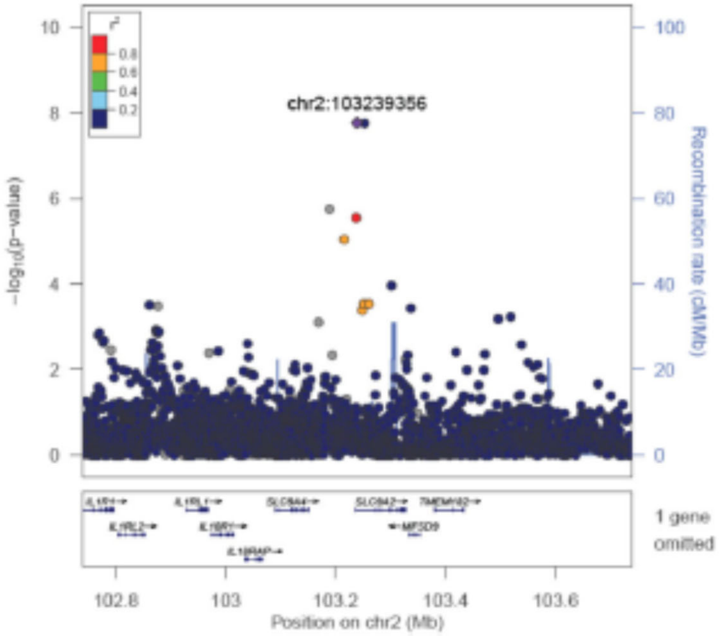

Locus 3: LDL-SomeCol in Asian Ancestry (2DF).

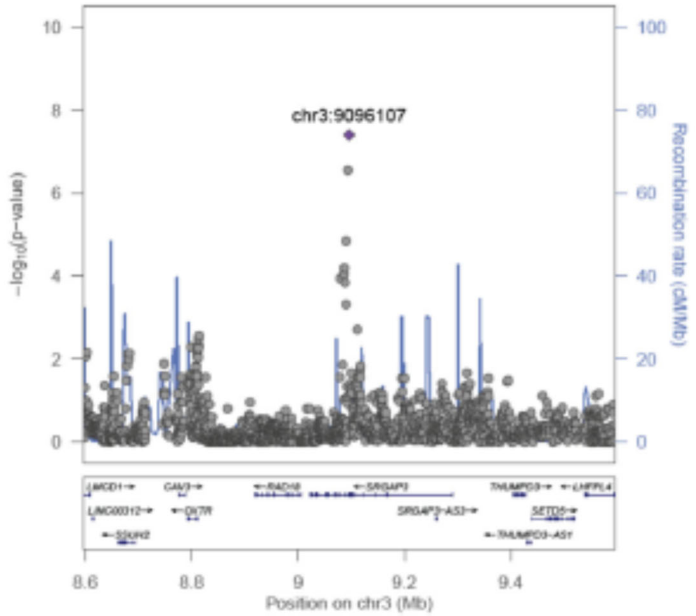

Locus 4: HDL-SomeCol in European Ancestry (left – interaction; right – 2DF).

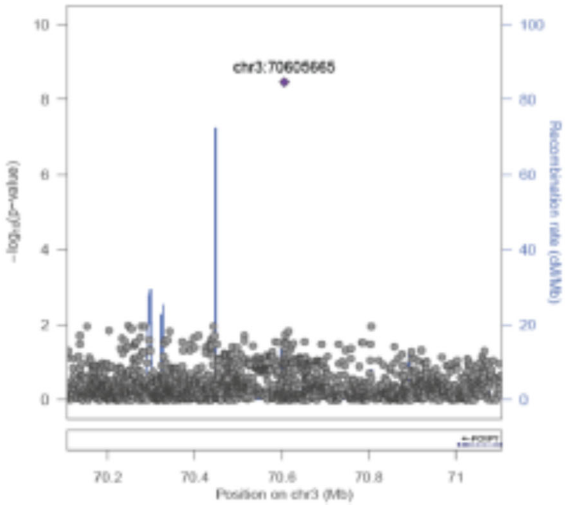

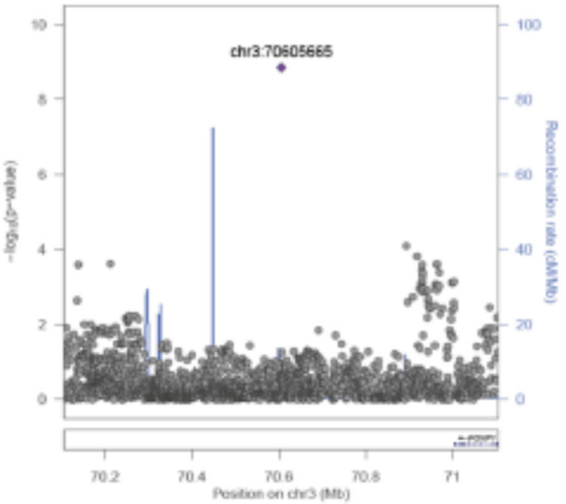

Locus 5: LDL-SomeCol in African Ancestry (2DF).

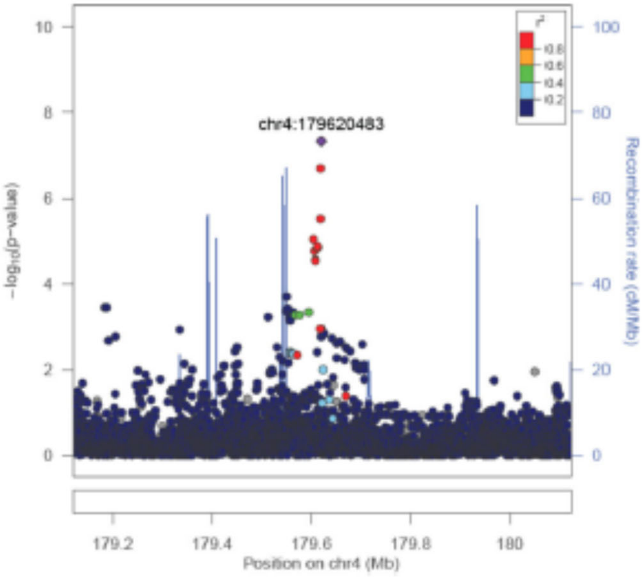

Locus 6: HDL-GradCol in Trans-Ancestry (left – interaction; right – 2DF).

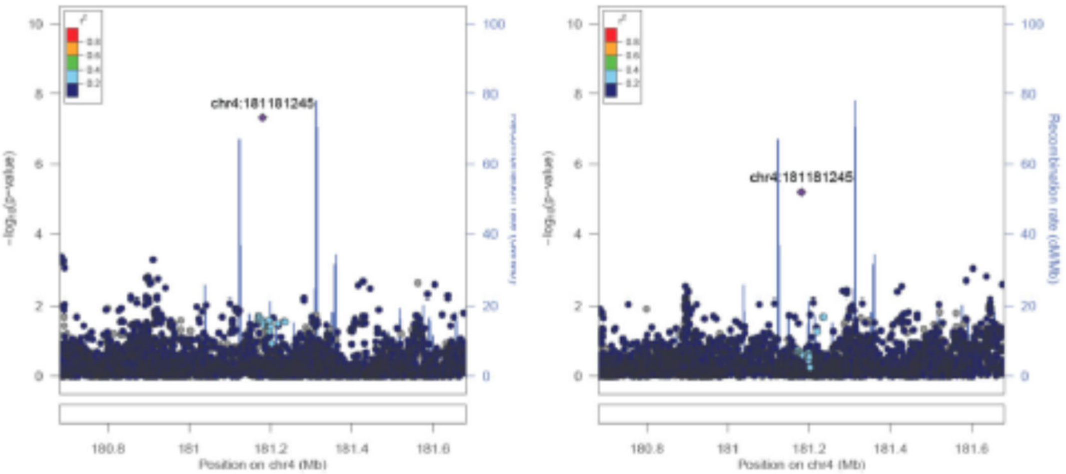

Locus 7: LDL-GradCol in Asian Ancestry (2DF).

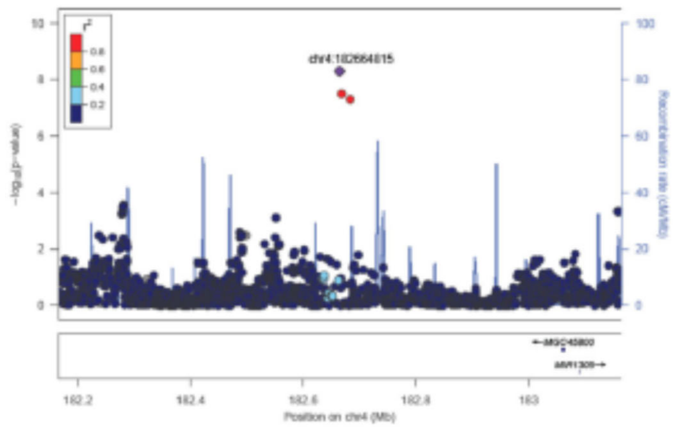

Locus 8: LDL-SomeCol in European Ancestry (2DF).

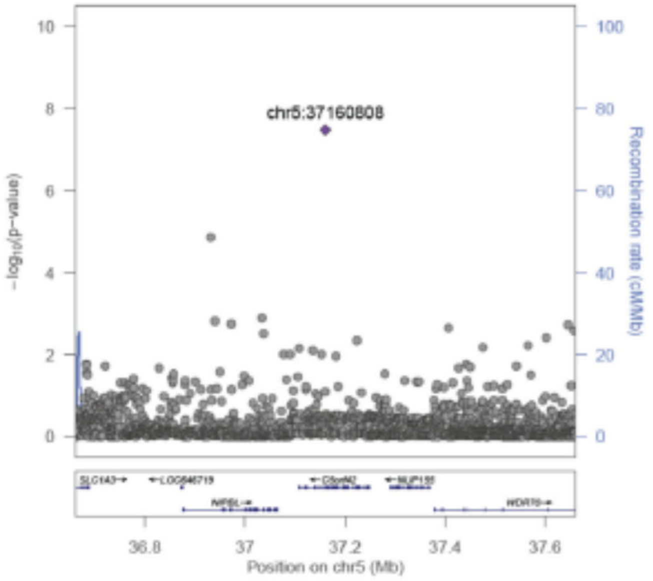

Locus 9: LDL-SomeCol in African Ancestry (2DF).

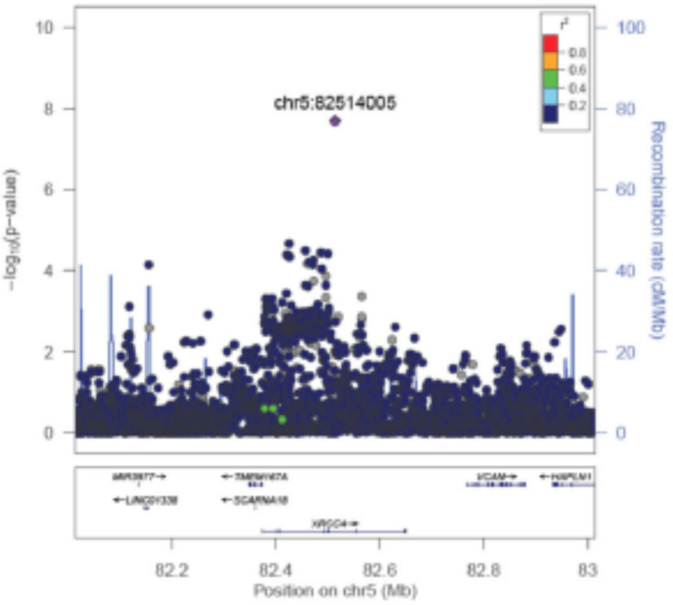

Locus 10: HDL-SomeCol in African Ancestry (2DF).

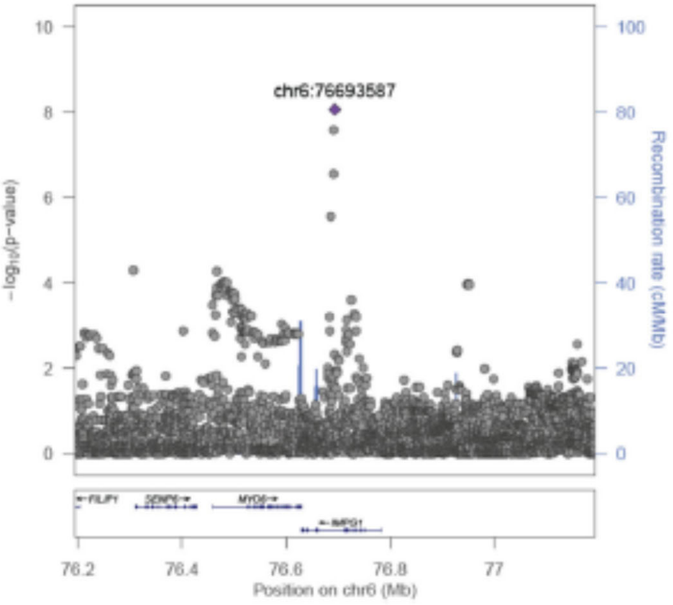

Locus 11: LDL-SomeCol in Trans-Ancestry (2DF).

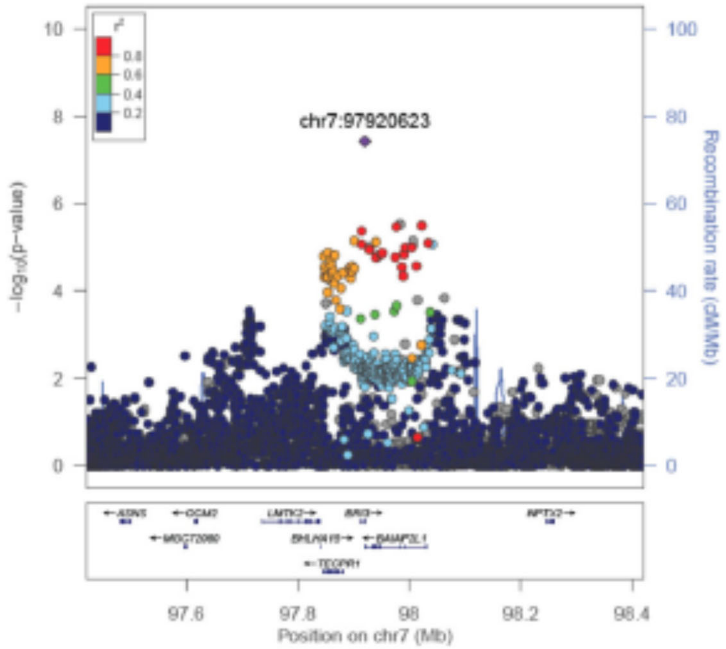

Locus 12: LDL-GradCol in African Ancestry (left – interaction; right – 2DF).

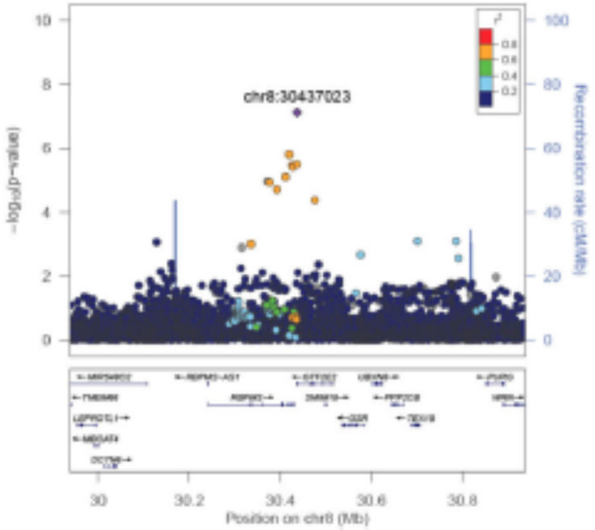

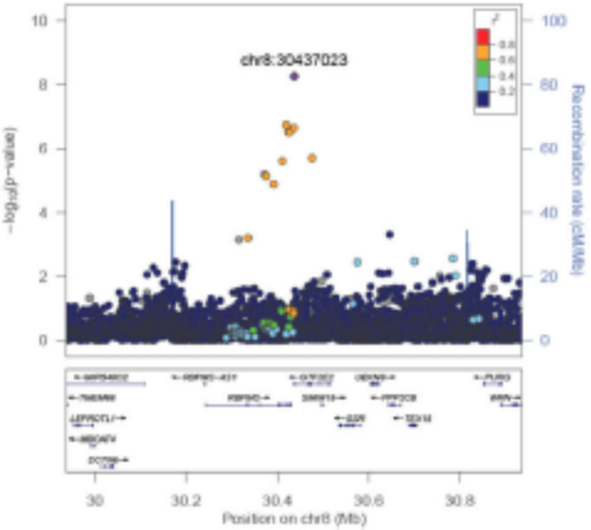

Locus 13: HDL-SomeCol in African Ancestry (2DF).

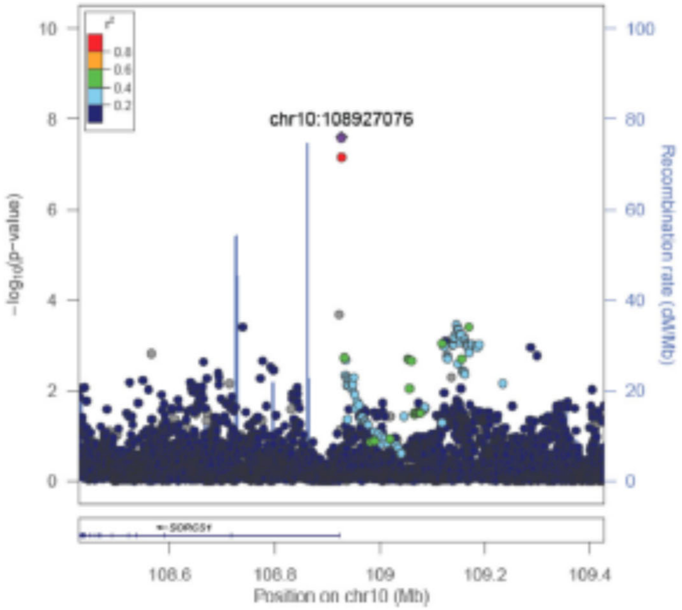

Locus 14: LDL-SomeCol in Trans-Ancestry (2DF).

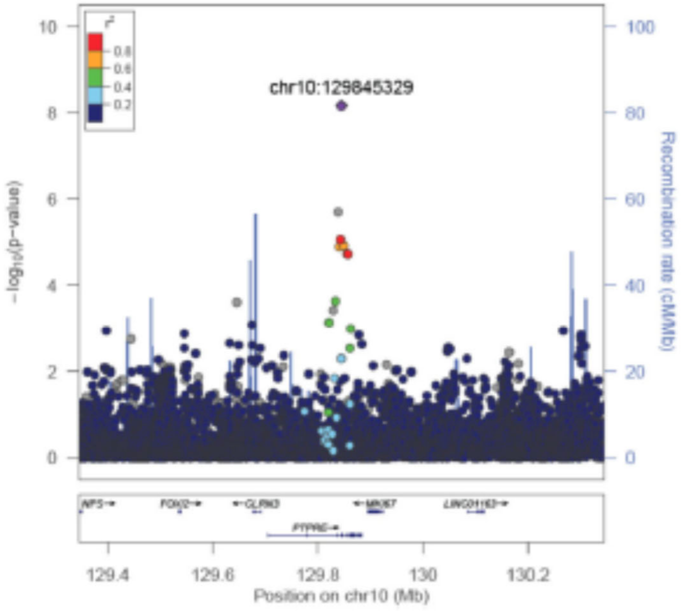

Locus 15: LDL-GradCol in Trans-Ancestry (left – interaction; right – 2DF).

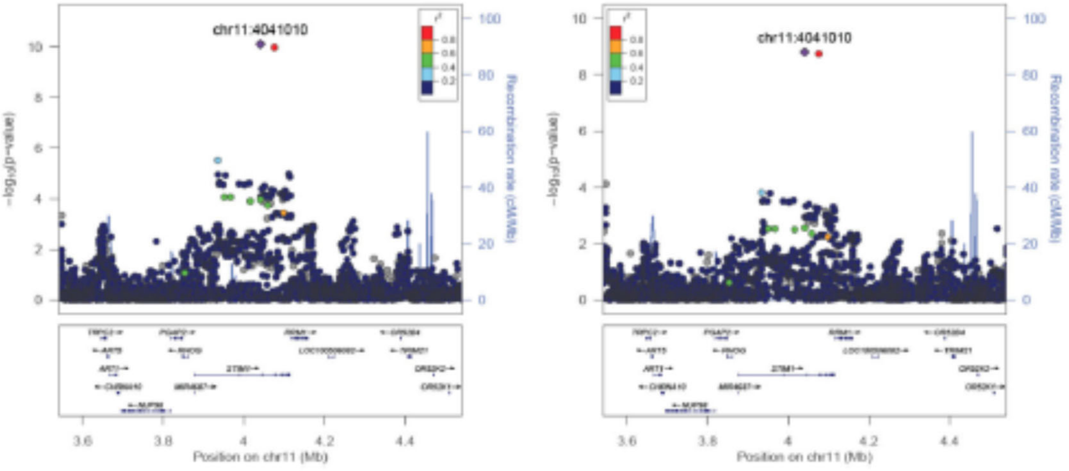

Locus 16: HDL-GradCol in European Ancestry (2DF).

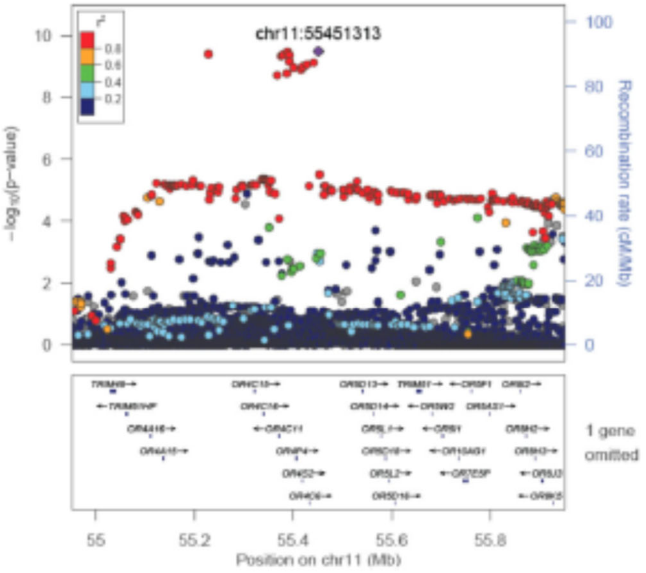

Locus 17: TG-GradCol in African Ancestry (2DF).

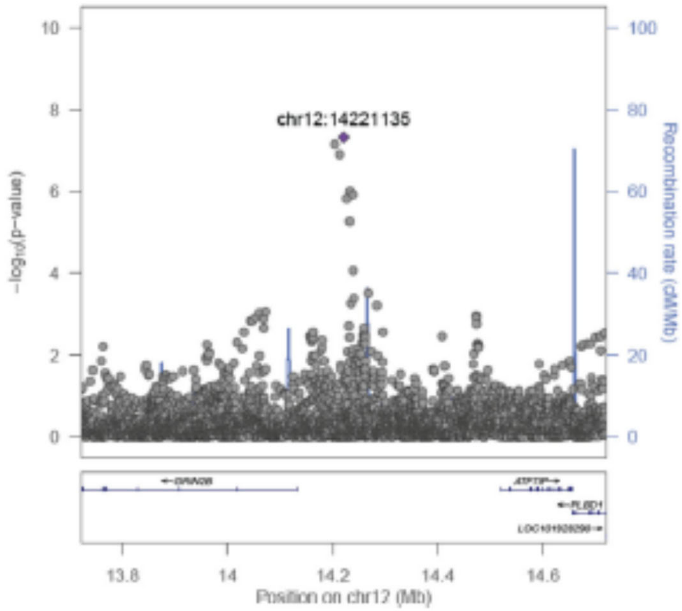

Locus 18: HDL-GradCol in African Ancestry (2DF).

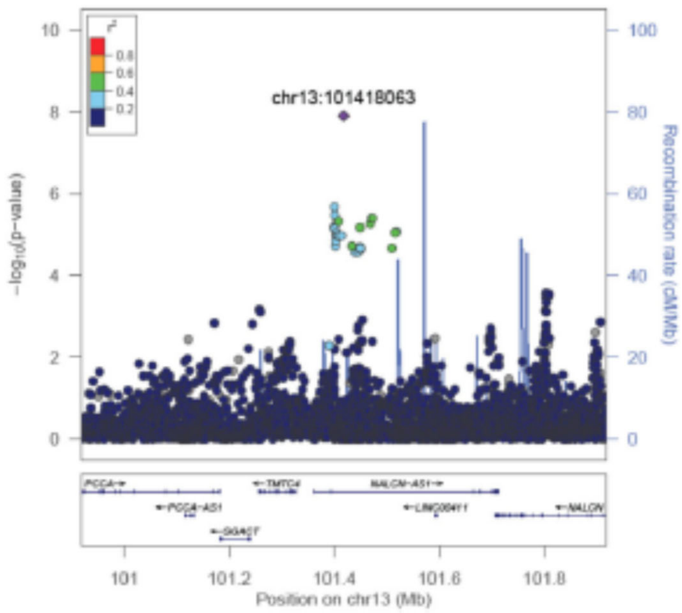

Supplement: Supplementary file 1 [file DataSheet1.PDF]
